# The effect of oligonucleotide microarray data pre-processing on the analysis of patient-cohort studies

**DOI:** 10.1186/1471-2105-7-105

**Published:** 2006-03-02

**Authors:** Roel GW Verhaak, Frank JT Staal, Peter JM Valk, Bob Lowenberg, Marcel JT Reinders, Dick de Ridder

**Affiliations:** 1 Department of Hematology, Erasmus Medical Center, Rotterdam, The Netherlands; 2 Department of Immunology, Erasmus Medical Center, Rotterdam, The Netherlands; 3 Information and Communication Theory Group, Faculty of Electrical Engineering, Mathematics and Computer Science, Delft University of Technology, Delft, the Netherlands

## Abstract

**Background:**

Intensity values measured by Affymetrix microarrays have to be both normalized, to be able to compare different microarrays by removing non-biological variation, and summarized, generating the final probe set expression values. Various pre-processing techniques, such as dChip, GCRMA, RMA and MAS have been developed for this purpose. This study assesses the effect of applying different pre-processing methods on the results of analyses of large Affymetrix datasets. By focusing on practical applications of microarray-based research, this study provides insight into the relevance of pre-processing procedures to biology-oriented researchers.

**Results:**

Using two publicly available datasets, i.e., gene-expression data of 285 patients with Acute Myeloid Leukemia (AML, Affymetrix HG-U133A GeneChip) and 42 samples of tumor tissue of the embryonal central nervous system (CNS, Affymetrix HuGeneFL GeneChip), we tested the effect of the four pre-processing strategies mentioned above, on (1) expression level measurements, (2) detection of differential expression, (3) cluster analysis and (4) classification of samples. In most cases, the effect of pre-processing is relatively small compared to other choices made in an analysis for the AML dataset, but has a more profound effect on the outcome of the CNS dataset. Analyses on individual probe sets, such as testing for differential expression, are affected most; supervised, multivariate analyses such as classification are far less sensitive to pre-processing.

**Conclusion:**

Using two experimental datasets, we show that the choice of pre-processing method is of relatively minor influence on the final analysis outcome of large microarray studies whereas it can have important effects on the results of a smaller study. The data source (platform, tissue homogeneity, RNA quality) is potentially of bigger importance than the choice of pre-processing method.

## Background

The analysis of gene expression data generated by microarrays, such as the high-density oligonucleotide microarays produced by Affymetrix (Santa Clara, CA), is an often laborious process in which a basic understanding of molecular biology, computer science and statistics is required. In a typical microarray experiment, RNA obtained under various conditions (patients, treatments, disease states etc.) is hybridised to microarrays. By tagging the RNA with a fluorescent marker, intensity values can be obtained that correspond to the amount of labeled RNA bound to the array. On the widely used Affymetrix platform, gene expression is measured using probe sets consisting of 11 to 20 perfect match (PM) probes of 25 nucleotides, which are complementary to a target sequence, and a similar number of mismatch (MM) probes in which the 13^*th *^nucleotide has been changed. The MM probe measurements are thought to comprise most of the background cross-hybridization and stray signal affecting the PM probes.

Normalization of probe intensity values is performed to remove any non-biological variation. The individual probe measurements are then summarized as probe set expression levels, as estimates of the amount of specific mRNA present in the biological sample. Normalization and probe set summarization are statistical procedures for which several methods have been developed. MicroArray Suite (MAS 5.0), a software package provided by Affymetrix, normalizes intensities using a global scaling procedure and measures expression using a one-step Tukey biweight algorithm, which is defined as the anti-log of a robust average of differences between log(PM) and log(MM) [1]. The same algorithms are implemented in the software package currently provided by Affymetrix, GCOS. One of the first alternatives to this approach was provided by Li and Wong with the dChip-method, which scales the intensity data towards the median intensity in a group of arrays and then uses model-based index estimates, giving variable weight to PM-MM probe pairs of a probe set based on variance between arrays, to measure expression [2]. Irizarry et al. introduced RMA (robust multi-array average), later followed by GCRMA (GC robust multi-array average). RMA, often preceded by quantile normalization [3,4], applies a median polish procedure to PM intensities only in summarization. GCRMA is based on a similar model as RMA but takes into account the effect of stronger bonding of G/C pairs [5,6]. An overview of these methods is shown in Table 1. Other normalization methods, such as the variance stabilizing normalization (VSN, [7]) and summarization methods, such as PLIER [8], have been developed, but are less frequently applied.

Various studies have been published which assess the differences in outcome of these different data pre-processing methods [9-14]. To validate and test pre-processing methods, two publicly available datasets are commonly used. The Latin square dataset provided by Affymetrix [15] contains spiked-in cRNA's at several concentrations facilitating the assessment of the relation between mRNA concentration and expression value. The GeneLogic dilution series (obtainable on request, [16]) gives an estimate of the relation between actual and measured differential expression. Based on these datasets, an online benchmark tool has been developed to encourage authors to test their method [17]. This tool assesses quality of pre-processing using several parameters in five different groups: (1) variability of expression across replicate arrays, (2) response of expression measure to changes in abundance of RNA, (3) sensitivity of fold-change measures to the amount of actual RNA sample, (4) accuracy of fold-change as a measure of relative expression and (5) usefulness of raw fold-change score for the detection of differential expression. Pronounced differences between different procedures have been shown to occur [4,9,11,13].

The studies on the Latin square and dilution data were performed using data generated specifically for this purpose, allowing comparisons of specific analyses, showing accurately which methods perform best. In effect, statistical properties of the various estimators are tested. Several authors noted that the use of two special-purpose datasets for calibration of statistical procedures creates a risk on overfitting of the available data and therefore focused on using experimental data to compare methods with respect to the sets of differentially expressed genes found [9,11,14]. This sometimes lead to contradictory results, where for instance a study using the Latin square dataset showed the MAS5.0 method to outperform the dChip method on detecting differentially expressed genes [13], while a study on experimental data showed the opposite [9]. Therefore, more work is needed to reliably establish how important the effect of choice of pre-processing method is in every-day practice, especially when analyses such as clustering and classification are applied.

In this paper, we focus on one practical application of microarrays: patient-cohort studies [18-22]. In such studies, researchers typically select sets of genes that are differentially expressed between certain known conditions, a supervised analysis. Moreover, unsupervised techniques (not imposing any prior knowledge on the data) such as clustering are applied to detect biological relations between samples or genes by grouping them according to their expression profiles. Often the goal is to obtain a predictor (classifier) for, for instance, prognostically relevant categories, using supervised analysis.

Given that different pre-processing procedures will influence the outcome of these analyses, several questions can be asked, such as: How well is expression measured using a number of different pre-processing methods? What is their effect on the detection of differentially expressed genes, clusters found and classification results? By focusing on practical applications of microarray studies, we hope to give insight into the relevance of different pre-processing procedures to biology-oriented researchers.

## Results and discussion

The aim of our work was to evaluate the effect of several microarray data pre-processing methods on the outcome of analyses commonly applied in patient-cohort studies. Four types of analysis were performed: (1) expression level measurement, (2) detection of differential expression (supervised), (3) cluster analysis (unsupervised) and (4) classification of samples (supervised). These analyses were applied to two publicly available datasets, one of 285 acute myeloid leukemia (AML) samples profiled on Affymetrix HG-U133A GeneChips [19] and one consisting of 42 Affymetrix HuGeneFL GeneChips hybridized with central nervous system (CNS) tumor tissue [22].

### Comparing expression levels to RT-PCR (AML dataset)

Pearson correlation coefficients between expression levels of *EVI1*, *CEBPA*, *MEIS1*, *HOXA7*, *HOXA9*, *TRKA*, *PRDM1*, *PRDM2*, *P8 *and *GMCSF *found in the AML dataset, measured by RT-PCR and microarray after pre-processing using different methods, are listed in Table 2. The actual expression levels measured by RT-PCR and the Affymetrix probe sets (using the different pre-processing methods) are listed in Supp. Table 2 (see Additional file 1). Average correlation to RT-PCR expression is 0.48–0.57 for the different methods with RMA (0.57 ± 0. 30) and GCRMA (0.57 ± 0.28) showing the highest correlation on average, dChip (0.48 ± 0.31) scoring lowest and MAS (0.52 ± 0.29) scoring intermediate. No significant differences have been found between correlations of different pre-processing methods and RT-PCR data and (taking RT-PCR as gold standard) no pre-processing method unequivocally performed best in measuring expression level. Correlation overall is moderate, but this result is likely to be influenced by different genomic location of RT-PCR primers and Affymetrix probes, resulting in different expression values when alternative splicing occurs; by incorrect annotation of individual probe sets (such as 206848_at); and by suboptimal RT-PCR primer and Affymetrix probe design.

### Comparing expression levels between pre-processing methods

**Figure 1:**
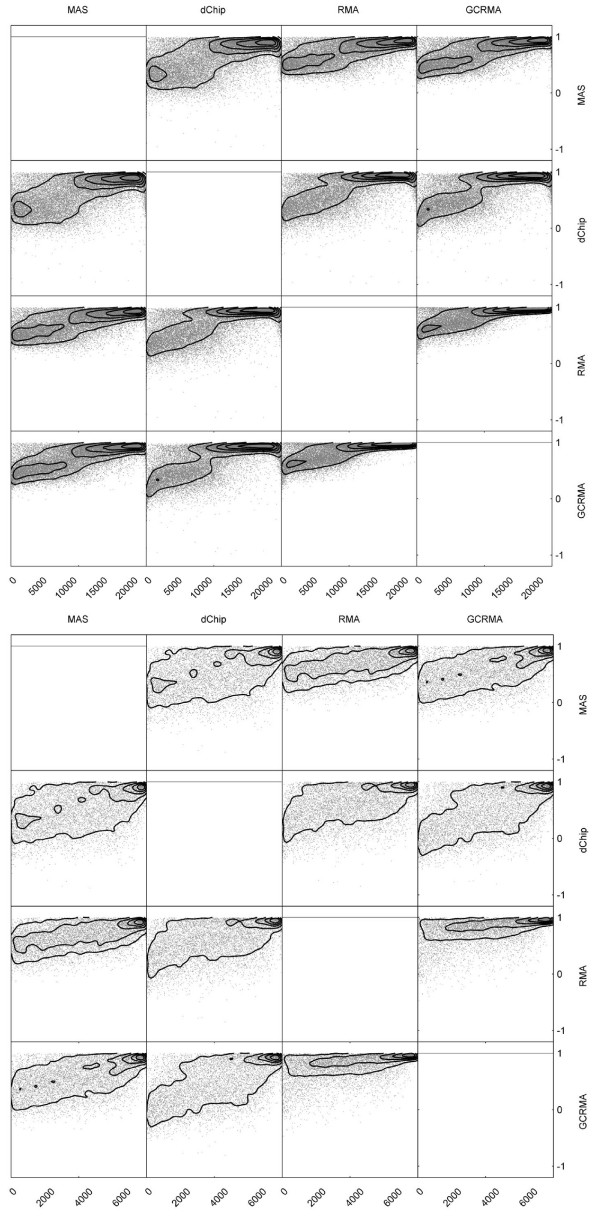
**Correlation of expression values pre-processed by two methods. **Pearson correlation coefficients of expression measurements calculated by two pre-processing procedures are shown on the *y*-axis, probe sets ranked by average expression level over the four pre-processing methods are shown on the *x*-axis. Contours indicate equal density, as estimated using a Gaussian kernel density estimate, with kernel width optimised by leave-one-out maximum-likelihood. A. AML dataset. B. CNS dataset.

Correlations are depicted in Figure 1A for the AML dataset and in Figure 1B for the CNS dataset for each probe set present on the microarray, ordered by average expression level over the four differently pre-processed datasets. Overall, a clear trend of increasing correlation at increasing expression levels is apparent, which has been noticed before [17]. Aside from a dense area of highly correlated genes with intermediate to high expression, in several comparisons, for instance that of RMA to MAS, a second more densely populated area is visible in the range of extremely low expression levels. These expression levels correspond to non-expressed genes (40–50% of all probe sets). At these levels, variability is relatively higher, resulting in moderate correlations. As the normalization method of RMA and GCRMA is the same (both using MM probes only for background correction) and their summarization methods are very similar, it is not surprising that these methods show the highest resemblance in measured expression. However, they show more agreement with MAS than with dChip (which was also seen when comparing microarray expression levels to RT-PCR expression levels). Perhaps this has to do with the fact that dChip calculates expression on the original probe intensity values rather than the log-transformed ones used by the other methods.

The CNS dataset shows similar trends, but the much higher level of variation suggests that sample size and/or quality of the platform and biological sample have a much more profound effect on estimated expression levels, than has the pre-processing method.

In conclusion, the AML dataset shows that variation in estimated expression levels exists between different pre-processing methods and that this variation is higher at lower mRNA concentrations. The clear trend of increasing correlation with increasing expression level suggests that pre-processing has an influence, but this concerns only a minority of probe sets.

Using the Affymetrix Latin square dataset, Rajagopalan noted that MAS and dChip perform equally well on estimating expression levels, with a small non-significant advantage for MAS [13]. Although trends towards MAS seem visible in our study as well, MAS and dChip behave rather differently in the experimental dataset used here.

Testing not only the Latin square dataset but also the GeneLogic dataset, Irizarry et al. conclude that RMA shows highest sensitivity and specificity when compared to dChip and the AvDiff algorithm [10]. As no method performs significantly different in our study, these results are not confirmed

### Differential expression

Significance of differences in expression when comparing two conditions was calculated using three standard methods: the *t*-test; the Wilcoxon rank sum test, controlling the Family-Wise Error Rate (FWER); and Significance Analysis of Microarrays (SAM), a test controlling the False Discovery Rate (FDR) using a statistic resembling that of the *t*-test [23]. Different pre-processing methods were compared by assessing the overlap in the number of probe sets marked as differentially expressed by two pre-processing methods. We call a probe set differentially expressed below an FWER or FDR of 5%. In the AML-dataset, *p*-values (FWER) and *q*-values (FDR) were computed for samples with recurrent FLT3 ITD mutations vs. the rest, inv(16) vs. the rest, t(15;17) vs. the rest and t(8;21) vs. the rest. In the CNS-dataset, *p*-values and *q*-values were computed for PNET-, RHAB-, GLIO- and MED-samples vs. the rest, respectively. Although different subdivisions into conditions were thus compared, the outcomes are remarkably similar.

Considering the AML dataset, the overlap between the probe sets selected on RMA and GCRMA pre-processed data is most striking, with a minimum *R *of 0.78 (average 0.85, Table 3, Supp. Tables 3A–C9 (see Additional file 1). Overall, the overlap between different pre-processing methods is considerable: a minimum *R *of 0.56 (average 0.74) is found, independent of the statistical test used. The combination MAS-dChip comes up as least comparable (Table 3, Supp. Tables 3A–C (see Additional file 1)). MAS shows higher concordance with RMA and GCRMA than does dChip. No indications were found that *R *will increase for smaller FWER or FDR (data not shown).

The overlap between probe set lists detected as differentially expressed is considerably less in the CNS dataset than in the AML dataset and there is more variation, which could be due to the higher amount of noise in this dataset and/or its smaller sample size. +The RMA-GCRMA comparison results in an average *R *of 0.56 (Table 4, Supp. Tables 3D–F (see Additional file 1)). Again, pre-processing with MAS will result in less differences with RMA pre-processing than with dChip (Table 4, Supp. Tables 3D–F (see Additional file 1)). Overall, average *R *is 0.40 for this dataset. When using *q*-values, again RMA and/or MAS often detect larger numbers of differentially expressed probe sets than dChip and GCRMA. Note also that the difference between the number of probe sets selected using the *t*-statistic and the Wilcoxon statistic is larger than for the AML dataset. This may be caused by outlier data on the HuGeneFL microarrays, to which the *t*-test is more susceptible.

In a study evaluating experimental datasets of 79 ovary tumors and 47 colon tumors profiled on the Affymetrix HG-U133A platform, Shedden et al. [9] show that dChip results are closer to those obtained using RMA than to those obtained using MAS, an observation not confirmed by our results. Statistical tests on RMA and MAS pre-processed data detect the largest number of differentially expressed probe sets in most cases, where GCRMA and dChip select less, with a maximum difference in number of selected probe sets of 49.7% in the AML dataset (Supp. Table 3A (see Additional file 1), *q*-values). This does not confirm the observations of Shedden et al., who found that dChip outperformed MAS and GCRMA in terms of sensitivity. Irizarry et al. [10] report that RMA performs better than dChip and the AvDiff algorithm in finding truly differentially expressed genes. No statement on the true nature of probe sets measured as differentially expressed here can be made. However, MAS and RMA score roughly equal numbers of probe sets as differentially expressed and both methods find more probe sets to be differentially expressed than dChip and GCRMA, as in [10].

Recently, Hoffmann et al. [11] stated that normalization will have a larger influence on the number of differentially expressed genes than the actual statistical test used. Although a direct comparison of [11] and our work is not possible due to differences in multiple testing correction, in our (much) larger datasets we observe a larger difference between the number of probe sets selected as a result of the multiple testing correction used (FWER or FDR) than as a result of the choice of pre-processing method.

Overall, the overlap between sets of genes selected as differentially expressed is considerable when pre-processing the data using different methods and overlap increases when non-biological variation decreases. Using the current datasets, it is not possible to give indications of the quality of probe sets selected, due to the lack of ground truth.

### Cluster analysis

**Figure 2:**
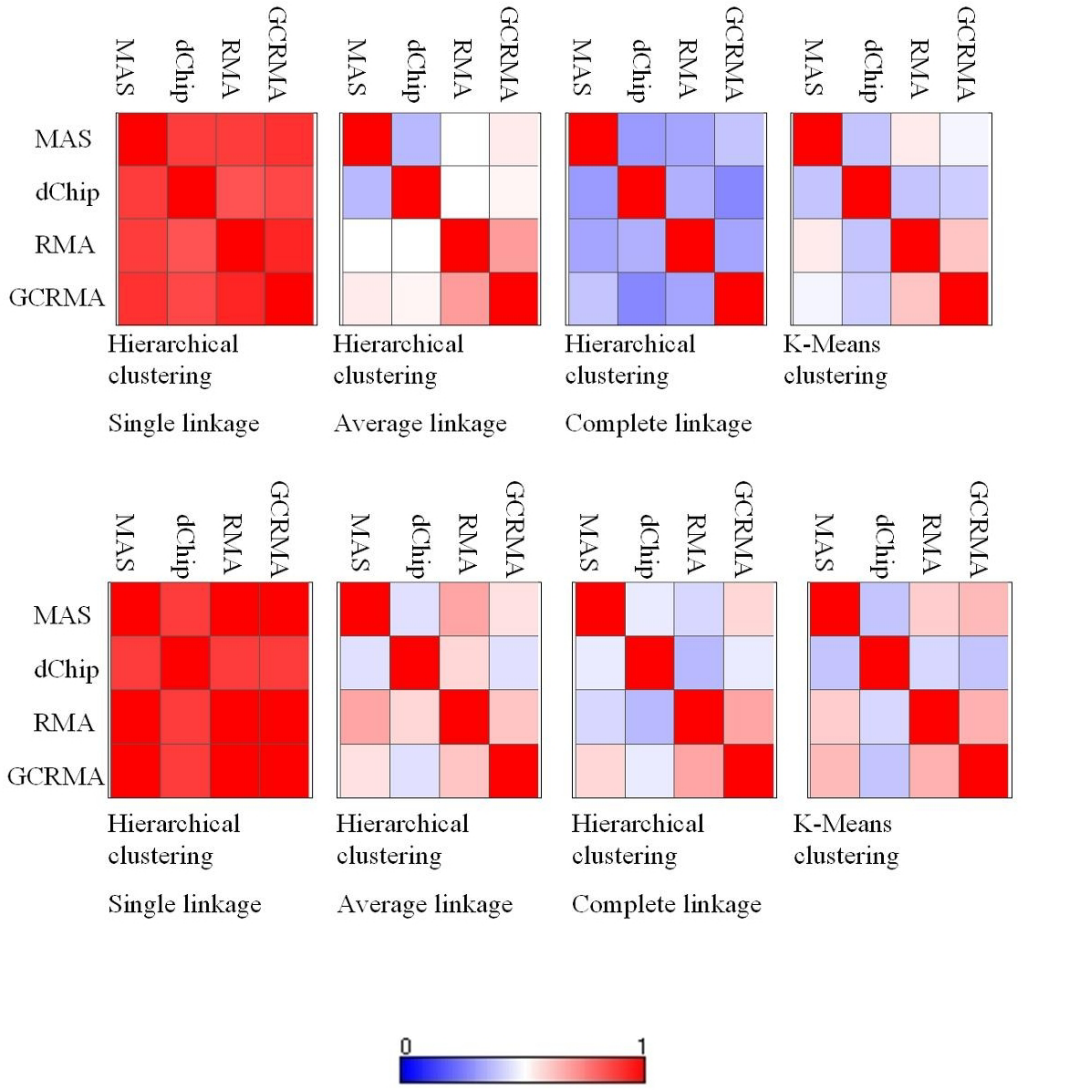
**Jaccard indices of clustering results. **Results were obtained using correlation distance on a fixed number of probe sets, after different pre-processing procedures and by different clustering algorithms. A. AML dataset, *k *= 12 clusters, 3000 probe sets. B. CNS dataset, *k *= 5 clusters, 1000 probe sets.

Data resulting from different pre-processing methods was clustered by *k*-means (KM) and hierarchical clustering with single, average and complete linkage (HC/S, HC/A, HC/C). Clusterings of both the AML and CNS datasets were compared using the Jaccard index; results are shown in Figure 2 and Supp. Figure 1 (see Additional file 1) [24]. RMA and GCRMA results are often similar, which is to be expected. dChip results frequently differ from results obtained using other pre-processing methods. In general, KM, HC/A and HC/C on both datasets show Jaccard indices of 0.3–0.6. HC/S shows higher indices, but the actual resulting clusterings are very poor due to the well-known high susceptibility of this method to outliers (data not shown): almost all samples end up in a single cluster, the remaining samples form individual clusters.

As an example, the confusion matrix in Table 5 shows that many clusters found using the MAS pre-processed dataset are also found reasonably well using the RMA pre-processed dataset (by *k*-means clustering into *k *= 12 clusters, on correlation distance, using 3000 probe sets). However, as there are 2716 sample pairs co-occurring in a cluster in both clustering results, 1099 sample pairs co-occurring in a cluster in the MAS clustering result only and 1137 sample pairs co-occurring in a cluster in the RMA result only, this leads to a Jaccard-index *J *of only 2716/(2716 + 1099 + 1137) = 0.55.

In an attempt to quantify the sensitivity of clusterings found to small perturbations, stability-normalized Jaccard indices *J*^*SN *^were therefore calculated, indicating to what extent the Jaccard indices *J *found are out of the ordinary. Figure 3A illustrates that for KM and the pair of pre-processing methods used (MAS and RMA), *J *= 0.55 is actually better than the Jaccard index obtained on average on a slightly changed version of the MAS pre-processed dataset (*J*^*SN *^> 0.5), but worse than that obtained on average on a slightly changed version of the RMA pre-processed dataset (*J*^*SN *^< 0.5).

Figure 3B shows that for KM and HC/A, differences using MAS and (GC)RMA are actually roughly of the same order as differences between 90% subsamples of the MAS pre-processed dataset (i.e. the *J*^*SN *^is high for MAS). To a lesser extent, this also holds for dChip vs. (GC)RMA. However, these same differences are quite large in terms of the differences in clusterings between 90% subsamples of (GC)RMA (i.e. the *J*^*SN *^is low for (GC)RMA). The main cause for this is (GC)RMA's higher stability: as it normalises over all arrays – unlike MAS and dChip – leaving out a small subset will have only a limited effect on probe set distributions, and hence on clustering results. When RMA and GCRMA results are compared to each other, a high *J*^*SN *^results as well. HC/C oftens shows lower values for *J *and *J*^*SN*^.

The CNS dataset (Figure 3C) largely tells the same story, although the *J*^*SN *^are somewhat larger, especially for *k*-means clustering. This is due to the smaller sample size: leaving out 10% of the samples relatively has more impact on the Jaccard indices.

Supp. Figures 1 and 2 (see Additional file 1) illustrate the influence of the choice of the number of clusters (*k*) and the distance measure (correlation or Euclidean). Both datasets show the same effects. For lower *k *(*k *= 2), both Jaccard indices and stability-normalized Jaccard indices are much higher, as clusterings of data pre-processed by the various methods agree on structure clearly present in the data. For higher *k *(AML: *k *= 20, CNS: *k *= 10), Jaccard indices and stability-normalized Jaccard indices are similar to or even lower than those for the *k *chosen originally. Using Euclidean distance leads to slightly lower Jaccard indices, with an increase in difference between Note however the more pronounced differences between how dChip and other methods. This may be the result of the negative values it produces (unlike MAS and (GC)RMA), which are thresholded at 0.1 in the data transformation steps. Due to the centering by the geometric mean this can lead to larger extreme probe set values over arrays.

In conclusion, clustering results are sensitive to the choice of pre-processing method. This sensitivity is smallest for small numbers of clusters *k *(i.e. when looking for clearly present structure) and when using correlation distance. Additionally, using (GC)RMA seems to result in more stable clusterings than using MAS or dChip.

### Classification

A number of different classification problems defined on the datasets have been approached using several classifiers trained on data of all pre-processing methods. Resulting performances are listed in Tables 6, 7, 8, 9 and Supp. Table 4 (see Additional file 1). Results are reported only for the number of probe sets giving lowest average test set error over the four methods. Although this makes the performance estimates biased, it does not influence comparison between methods.

In the AML dataset, inversion of chromosome 16 is well predictable, with error rates smaller than 5% (Table 6). Differences in error rate between classification algorithms are very small: although the nearest centroid classifier often performs worst, no algorithm performs significantly better than others. More importantly, no pre-processing method scores significantly better or worse than others (although MAS relatively often shows best results). Although predicted with a higher error rate, these observations are confirmed on the FLT3 (Table 7) and CCR (Table 8) AML problems.

Interestingly, this also holds for the CNS dataset (Table 9 shows results for the MED problem; other results are shown in Supp. Table 4 (see Additional file 1)), although performances show much more variation and MAS no longer comes out best. Ofcourse, the CNS dataset is rather small, so obtaining good classifiers is harder.

No classifier or pre-processing method scores significantly better than others. This can be explained by the fact that the probe sets on which classification is based are already selected to give good classification results: on differently pre-processed datasets, different probe sets may be selected (in fact, the selected sets of *n *= 1000 probe sets show an overlap of 71% to 87%). The six classifiers used seem to be equally susceptible to different pre-processing methods; that is, for each of them performance varies with pre-processing method used in at least some of the problems.

For classification, the choice of pre-processing method (and, for that matter, classification algorithm) seems to be irrelevant.

## Conclusion

Patient-cohort studies using microarrays are often performed to find pathobiologically relevant relations between genes and patient classes. The Affymetrix platform has become increasingly popular for this type of study. Processing intensity values obtained using Affymetrix GeneChips remains a challenging task for many microarray researchers. Apart from the Affymetrix MAS procedure, several statistical procedures have been proposed to assess expression, such as dChip, RMA and GCRMA. Our study has tried to estimate the effects of the choice of pre-processing method from a practical viewpoint. To this end, we have applied a number of analyses to two datasets, which we believe to represent two extremes in recent patient-cohort studies both in terms of sample size and of platform used.

The experimental results indicate that the normalization step in (GC)RMA has a larger effect on the data than the one in MAS and dChip, but this cannot be separated from the effect of applying different models for summarization. And, although the dChip and RMA summarization models are more related to each other than the MAS and RMA ones, MAS pre-processed data shows more similarity to RMA than does dChip.

In practical terms, the question of which method will give expression value estimates closest to the actual data is still to be answered; this study has not attempted to answer it, because we have not used data with accompanying ground truth. We showed that results of various analyses are not always dependent on the choice of pre-processing method. Analyses such as calculating expression levels or assessing differential expression are reasonably susceptible to differences between pre-processing methods; clustering as well, except when looking for clearly present structure (that is, using a small number of clusters); but classification far less so. The message is that while care should be taken in assigning biological meaning to individual probe set measurements, this holds less for global statements about the data.

Several other studies have been performed to assess the level of concordance in differential gene sets between pre-processing methods and noted that the choice of the method was of major influence, with different studies favoring different pre-processing methods [9,11,13]. Our results do not conclusively confirm one or more studies, although results partially overlap. One major difference with other studies is the size of the used datasets, where one of the datasets used in this study is considerably larger. It is to be expected that with the evolution of the array technology, the number of profiled samples in any single patient-cohort study is likely to increase.

The effects of the choice of pre-processing method are far more profound in the CNS dataset than in the AML dataset. Several possible explanations can be given for this, but it is not possible to single any of them out based only on the two datasets used in this study. The AML dataset contains more samples, which allows for better parameter estimates in the analysis methods presented in this work. Furthermore, Affymetrix technology has evolved over time, resulting in a more stable platform for the AML dataset (HG-U133A) than the CNS dataset (HuGeneFL). Biological differences also play a role in the two datasets. The amount of viable cells obtained from bone marrow is also likely to be higher compared to solid tumors, which often show necrotic areas, leading to difference in RNA-quality and -degradation. Also, tumor cells can be purified from bone marrow samples using Ficoll-centrifugation, a technique which is not available for the solid tumors which were hybridized in the CNS dataset, resulting in less contamination with other cell types in hybridized samples, which is known to be an important factor [25]. We recommend that the emphasis in setting up a large microarray-based study should therefore be on the quality of the biological sample and the quality of RNA rather than on the choice of the pre-processing procedure. However, we do believe that an inverse relation exists, with the importance of the method of normalization and expression summarization increasing when the quality of the biological sample and the number of studied samples decrease. Although we base this on a limited number of pre-processing methods and data sets, we think that taking into account more available methods will have no effect on our conclusion.

## Methods

### Datasets

The two datasets used have been described before [19,22]. The first dataset consists of microarray measurements taken on samples of 285 patients with acute myeloid leukemia (AML), of whom blasts and mono-nuclear cells were isolated from peripheral blood or bone marrow aspirates. The samples were hybridized on Affymetrix HG-U133A GeneChip microarrays. This dataset will be referred to as the AML dataset; it is available on the Gene Expression Omnibus website ([26], accession number GSE1159). The second dataset contains gene-expression data of 42 homogenized tumor tissues of the embryonal central nervous system (CNS), hybridized on Affymetrix HuGeneFL arrays. The dataset, referred to as the CNS dataset, is available at [27].

### Normalization and expression measurement

Both datasets were pre-processed with MAS, RMA, GCRMA and dChip, resulting in eight different datasets. MAS expression data combined with global scaling was obtained from the MAS 5.0 software, provided by Affymetrix (Affymetrix Inc., Santa Clara, CA). dChip pre-processing together with scaling of the data towards the median average expression value per chip was applied using software available from the authors [28]. RMA and GCRMA pre-processing was performed together with quantile normalization using the Bioconductor v2.0 library available in the R software environment [29].

### Real-time quantitative PCR (RT-PCR)

For the AML dataset only, a number of measured probe set expression levels were compared to available RT-PCR measurements of the corresponding genes on subsets of the original dataset (with *n *varying between 208 and 277, as indicated in Supp. Table 2 (see Additional file 1)). Probe sets were selected for RT-PCR measurement based on biological relevance to the study of leukemia; samples were selected based on availability of material. Eligible patients had a diagnosis of primary AML, confirmed by cytological examination of blood and bone marrow. After informed consent, bone marrow aspirates or peripheral blood samples were taken at diagnosis. Blasts and mononuclear cells were purified by Ficoll-Hypaque (Nygaard, Oslo, Norway) centrifugation and cryopreserved. The AML samples contained 80–100 percent blast cells after thawing, regardless of the blast count at diagnosis.

After thawing, cells were washed once with Hanks balanced salt solution. High quality total RNA was extracted by lysis with guanidinium isothiocyanate followed by cesium chloride gradient purification. RNA concentration, quality and purity were examined using the RNA 6000 Nano assay on the Agilent 2100 Bioanalyzer (Agilent, Amstelveen, The Netherlands). None of the samples showed RNA degradation (28S/18S rRNA ratio ≥ 2) or contamination by DNA.

cDNA was synthesized from 1ìg of RNA using random hexamer priming, essentially as described [30]. cDNA prepared from 50ng of RNA was used for all RT-PCR amplifications.

Real-time quantitative PCR amplification was performed with the ABI PRISM 7700 sequence Detector (Applied Biosystems, Nieuwerkerk aan den IJssel, Netherlands), using 50 μL mix containing 20 μM deoxyribonucleoside triphosphates (dNTPs; Amersham Pharmacia Biotech, Roosendaal, Netherlands); 15 pmol forward and reverse primer (Life Technologies); 3 mM MgCl2 (5 mM for the reference gene porphobilinogen deaminase [PBGD]); 10 pmol probe (Eurogentec, Maastricht, Netherlands); 5 μL 10 × buffer A and 1.25 U AmpliTaq Gold (Applied Biosystems). The primers and probe combinations for detection of *EVI1 *[EMBL:BX647613] [31], *CEBPA *[RefSeq:NM_004364.2] [32], *TRKA *[EMBL:M23102] [33] and *PBGD *[EMBL:AB162702] [33] have been described. Primer and probe combinations used to determine the expression of *MEIS1 *[EMBL:AB040810], *HOXA7 *[EMBL:AJ005814], *PRDM1 *[EMBL:AL358952], and *PRDM2 *[EMBL:U23736] are listed in Supp. Table 1 (see Additional file 1). Expression of *HOXA9 *[EMBL:BC006537], *GMCSF *[EMBL:X03021], *P8 *[EMBL:AF135266] was measured with 1 × SYBR Green I dye (Applied Biosystems). The primers used in the SYBR Green reactions are listed in Supp. Table 1 (see Additional file 1). The thermal cycling conditions included 10 minutes at 95°C followed by 45 cycles of denaturation for 30 seconds at 95°C and annealing/extension at 60°C for 60 seconds.

To quantify the relative expression levels of the various genes in AML the Ct values were normalized for the endogenous reference PBGD (ΔCt = Ct_target _- Ct_PBGD_) and compared with a calibrator NBM cells from healthy volunteers, using the ΔΔCt method (ΔΔCt = ΔCt _AMLsample _- ΔCt_Calibrator_). We used the ΔΔCt value to calculate relative expression (2^-ΔΔCt^).

A minimum threshold of 1 was applied, as well as log(2) transformation [34]. Pearson correlation coefficients were calculated between the RT-PCR data and the corresponding microarray-data pre-processed by the different procedures. Pearson correlation coefficients between data from the different procedures were also calculated, for each probe set present on the microarray.

### Data transformation

For each probe set, the geometric mean *m *of all expression values *e *over the different samples was calculated. The level of expression for a particular sample was subsequently determined as log_2_(*e*)-log_2_(*m*). This transformation was applied to all datasets and only transformed data was used for detection of differential expression, cluster analysis and classification.

### Differential expression

Tests for differential expression were performed on several biologically relevant groups, by comparing samples from a group to the remainder of the samples. Four groups were tested in the AML dataset: (1) samples with a recurrent mutation in the FLT3 gene (*n *= 78), (2) samples with inversion of chromosome 16, (inv(16), *n *= 23), (3) samples with translocation of chromosomes 15 and 17 (t(15;17), *n *= 19) and (4) samples with translocation of chromosomes 8 and 21 (t(8;21), *n *= 22). In the CNS dataset, four groups were tested as well: (1) samples with primitive neuro-ectodermal tumors (PNET, *n *= 8), (2) samples with medullablastomas (MED, *n *= 10), (3) samples with rhabdoid tumors (RHAB, *n *= 10) and (4) samples with malignant gliomas (GLIO, *n *= 10).

Student's *t*-test and Wilcoxon's rank sum test were applied to each probe set [35]. The resulting *p*-values were adjusted for multiple testing by Šidák step-down adjustment to control the Family-Wise Error Rate or FWER [36]. The Significance Analysis of Microarrays (SAM) permutation algorithm (Excel-version 1.21, [37]), controlling the False Discovery Rate (FDR), was also applied [23]. SAM provides an estimate of the FDR known as a *q*-value.

Each test was applied and lists of probe sets, considered significantly differentially expressed at an FWER or FDR of 5%, were retrieved. For all possible combinations (i.e. MAS-dChip, MAS-RMA, MAS-GCRMA, dChip-RMA, dChip-GCRMA and RMA-GCRMA) probe sets marked as significantly differentially expressed by both methods were counted. To be able to compare different combinations, an overlap ratio *R*(*A*,*B*) was calculated between the number of probe sets detected as differentially expressed in both datasets *A *and *B *and the total number of unique probe sets detected in the two datasets:

R(A,B)=2pa+b
 MathType@MTEF@5@5@+=feaafiart1ev1aaatCvAUfKttLearuWrP9MDH5MBPbIqV92AaeXatLxBI9gBaebbnrfifHhDYfgasaacH8akY=wiFfYdH8Gipec8Eeeu0xXdbba9frFj0=OqFfea0dXdd9vqai=hGuQ8kuc9pgc9s8qqaq=dirpe0xb9q8qiLsFr0=vr0=vr0dc8meaabaqaciaacaGaaeqabaqabeGadaaakeaacqWGsbGucqGGOaakcqWGbbqqcqGGSaalcqWGcbGqcqGGPaqkcqGH9aqpdaWcaaqaaiabikdaYiabdchaWbqaaiabdggaHjabgUcaRiabdkgaIbaaaaa@396E@

where *p *is the number of probe sets significant in both datasets, *a *is the number of significant probe sets found in dataset *A *and *b *is the number of significant probe sets found in dataset *B*.

### Cluster analysis

Subsets of *n *probe sets (for the AML dataset, *n *= 3000; for CNS, *n *= 1000) were created by ranking probe sets by their standard deviation over all samples, and selecting the top *n*. Samples in all datasets (4 pre-processing methods) were clustered using *k*-means clustering and hierarchical clustering on both correlation distance matrices, in which the distance between two samples *x *and *y *is defined as 1-ρ_*xy*_; and Euclidean distance matrices, as used in [19]. Hierarchical clustering was performed using single, average and complete linkage. To be able to compare all methods and datasets, the number of clusters was fixed to the expected number of groups based on biological characteristics of the patient population, which was 12 for the AML dataset and 5 for the CNS dataset. To investigate the influence of this setting, the AML dataset was also clustered into 2 and 20 clusters and the CNS dataset was clustered into 2 and 10 clusters, respectively. During each run of the *k*-means algorithm it was randomly restarted 1000 times, retaining the solution yielding minimum cluster within-scatter, in an attempt to avoid local minima.

Clustering results were compared using the Jaccard index. The Jaccard-index *J*(*C*_1_,*C*_2_) compares two clusterings *C*_1 _and *C*_2 _based on the number of similar sample pairs available in the clusters and results in a value between 0 (no similar pairs) and 1 (all pairs are equal). It is estimated as

J(C1,C2)=n12n12+n1+n2
 MathType@MTEF@5@5@+=feaafiart1ev1aaatCvAUfKttLearuWrP9MDH5MBPbIqV92AaeXatLxBI9gBaebbnrfifHhDYfgasaacH8akY=wiFfYdH8Gipec8Eeeu0xXdbba9frFj0=OqFfea0dXdd9vqai=hGuQ8kuc9pgc9s8qqaq=dirpe0xb9q8qiLsFr0=vr0=vr0dc8meaabaqaciaacaGaaeqabaqabeGadaaakeaacqWGkbGscqGGOaakcqWGdbWqdaWgaaWcbaGaeGymaedabeaakiabcYcaSiabdoeadnaaBaaaleaacqaIYaGmaeqaaOGaeiykaKIaeyypa0ZaaSaaaeaacqWGUbGBdaWgaaWcbaGaeGymaeJaeGOmaidabeaaaOqaaiabd6gaUnaaBaaaleaacqaIXaqmcqaIYaGmaeqaaOGaey4kaSIaemOBa42aaSbaaSqaaiabigdaXaqabaGccqGHRaWkcqWGUbGBdaWgaaWcbaGaeGOmaidabeaaaaaaaa@43A9@

where *n*_12 _denotes the number of pairs of samples in the same cluster in *C*_1 _and assigned to the same cluster in *C*_2_, *n*_1 _denotes the number of pairs in the same cluster in *C*_1_, but in different clusters in *C*_2 _and *n*_2 _denotes the number of pairs in the same cluster in *C*_2_, but in a different cluster in *C*_1_.

The raw Jaccard index should be interpreted in the light of how stable *C*_1 _and *C*_2 _actually are. If a clustering *C*, obtained using a certain pre-processing method, changes when one or a few samples are removed, it is to be expected that using a different pre-processing method will also have an impact. To estimate stability, for each pre-processing method 100 pairs of random subsets each containing 90% of the samples were clustered. Each individual subset was transformed as described and *n *= 3000 (or *n *= 1000 for the CNS dataset) probe sets were selected (these sets were 97.1% identical on average). In each pair, both subsets were then clustered, and the Jaccard index between these two clusterings was calculated using the samples present in both subsets. This resulted in 100 Jaccard indices, giving an impression of the variability due to transformation and subset selection. Finally, normal distributions were fitted to the 100 Jaccard indices found.

For a Jaccard index resulting from a comparison between two pre-processing methods *M*_1 _and *M*_2_, the cumulative distribution function (CDF) of the normal distribution for both pre-processing methods is used to arrive at two stability-normalized Jaccard indices *J*_1_^*SN *^and *J*_2_^*SN*^. Figure 3A illustrates this. A value of 0.5 for *J*_*i*_^*SN *^(in Figure 3A obtained at a Jaccard index of 0.48 for MAS or 0.62 for RMA) indicates that differences between pre-processing methods fall well within the range of clustering variability for pre-processing method *M*_*i*_; values higher than that indicate that clustering differences due to pre-processing are in fact smaller than the average differences between clusterings on subsampled datasets. Although the notion of stability has been used before in clustering (e.g. [38]), we believe this normalized index to be novel.

Note that for the k-means algorithm, stability-normalised Jaccard indices are displayed in Figures 3B and 3C as mean and standard deviation over 10 runs of the algorithm, each run the result of 1000 restarts (see above).

### Classification

Three two-class problems were defined on the AML dataset: (1) samples with inversion of chromosome 16 (inv(16) vs. all others, (2) samples with a mutation in the FLT3-gene vs. all others and (3) samples that showed continuous complete remission (CCR) vs. samples that did not. These problems were selected in increasing order of expected difficulty. In the case of the CNS dataset, four two-class problems (PNET vs. others, MED vs. others, RHAB vs. others and GLIO vs. others) were defined. A number of classifiers were trained on probe set subsets of increasing size (*n* = 10, 20, 50, 100, 200, 500, 1000). Probe sets were selected here using a signal-to-noise ratio (SNR) variation filter, i.e. |μ_1_-μ_2_|/√(σ_1_^2 ^+ σ_2_^2^) on the training set. Classifiers used were nearest centroid (NC), nearest shrunken centroid (PAM) [39], LIKNON [40], k-nearest neighbour (*k*-NN), support vector classifier with polynomial kernel of degree d (SVC-P) and radial basis function kernel of width σ (SVC-R) [41]. The parameters *k*, *d* and σ were optimised by performing cross-validation (*k*: leave-one-out; *d*, σ : 10-fold) on the training set only. Both PAM and LIKNON provide their own feature selection algorithm, which selects the optimal feature set within the set selected by the variation filter. In a single experiment, 90 percent of the samples (randomly selected) were used to train a classifier after which the classifier was tested on the remaining 10 percent. This experiment was repeated 100 times, resulting in an average performance and a standard deviation.

## List of Abbreviations

AML Acute myeloid leukemia

CNS Central nervous system

PNET Primitive neuro-ectodermal tumors

MED Medullablastoma

GLIO Malignant glioma

RHAB Rhabdoid tumors

SAM Significance analysis of microarrays

CDF Cumulative distribution function

CCR Continuous complete remission

PAM Prediction analysis of microarrays

k-NN k-Nearest neighbour

NC Nearest centroid

SVC-P Support vector classifier with polynomial kernel of degree *d*

SVC-R Support vector classifier with radial basis function kernel of width σ

## Authors' contributions

RGWV and DDR participated in all phases of research. PJMV assisted in study design. FJTS, BL and MJTR gave intellectual contributions. All authors and read and approved the manuscript.

## Supplementary Material

Additional File 1 Short description: Supplemental tables 1, 2, 3, 4, Supplemental figures 1, 2. Click here for file

## Figures and Tables

**Figure 3A:**
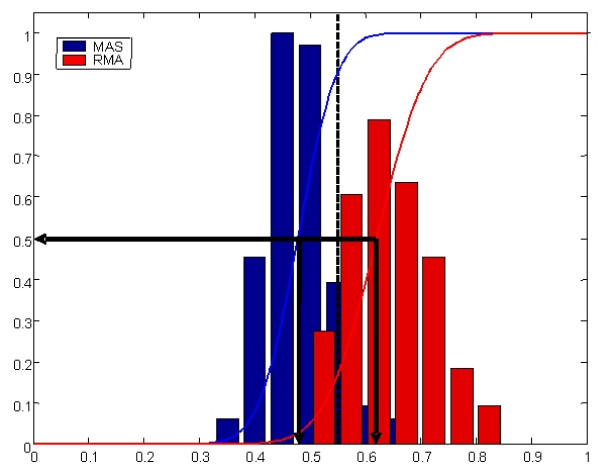
**Stability normalization of Jaccard index**. Illustration of stability normalization for the Jaccard index of a particular *k*-means clustering (*k *= 12), obtained on MAS- and RMA-pre-processed versions of the AML dataset (correlation distance, 3000 probesets). The dotted line corresponds to the Jaccard index between these clusterings (0.55). For both MAS and RMA, the CDF can be used to arrive at a stability normalized Jaccard index; in this case 0.90 and 0.16. The arrows indicate the Jaccard indices for which the normalised Jaccard index *J*^*SN *^= 0.5. The interpretation is that for MAS, the comparison to RMA falls well within what can be expected, for RMA less so.

**Figure 3B:**
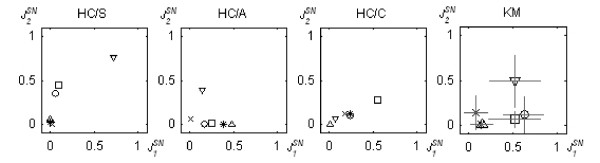
**B: AML dataset: stability-normalized pairwise Jaccard indices of cluster labels assigned by the various methods. **Clusterings into *k *= 12 clusters obtained using correlation distance on 3000 probe sets. Legend is shown in Figure 3D. For *k*-means, the grey bars indicate standard deviation over 10 repeated experiments.

**Figure 3C:**
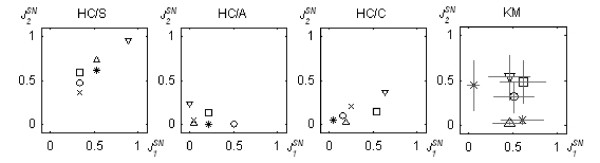
**CNS dataset: stability-normalized pairwise Jaccard indices of cluster labels assigned by the various methods. **Clusterings into *k *= 5 clusters obtained using correlation distance on 1000 probe sets. Legend is shown in Figure 3D. For *k*-means, the grey bars indicate standard deviation over 10 repeated experiments.

**Figure 3D:**
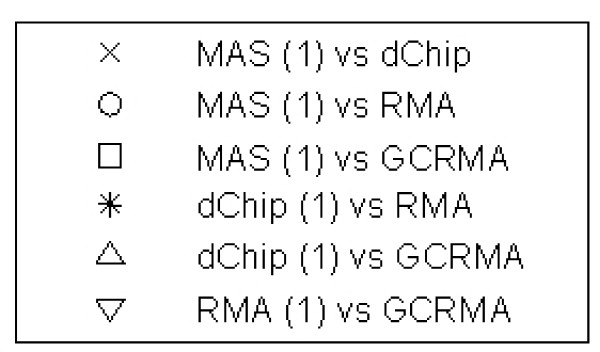
D: Legend to markers in Figures 3B-C.

**Table 1:** Overview of several pre-processing methods.

	**Normalization method**	**Summarization method**
**MAS**	Global scaling – individual array normalization; moderate influence on expression levels, no effect on outliers. Non-parametric methods are potentially more reliable	Tukey biweight (robust average) – subtract MM from PM and adjust for negative values
**dChip**	Average median scaling – individual array normalization; moderate influence on expression levels, no effect on outliers. Non-parametric methods are potentially more reliable	Model-based index estimate – subtract MM from PM, but take indivual probe variability, assessed over all available arrays, into account
**RMA**	Quantile normalization – multiple array normalization; considerable influence on expression levels, with removal of outliers. Parametric methods are potentially more reliable	Median polish – only use MM for background adjustment; fit parameters of linear model robustly using median polish, taking into account all available arrays
**GCRMA**	Quantile normalization – multiple array normalization; considerable influence on expression levels, with removal of outliers. Parametric methods are potentially more reliable	Median polish – only use MM for background adjustment; fit parameters of linear model robustly using median polish, taking into account all available arrays; fit extra GC-content parameter

**Table 2:** Correlation between expression levels measured by RT-PCR and Affymetrix GeneChip on the AML dataset, after use of different pre-processing methods.

**Gene symbol**	**Probe set ID**	**MAS**	**dChip**	**RMA**	**GCRMA**
***EVI1***	**215851_at**	0.34	0.52	0.63	0.29
	**221884_at**	0.64	0.87	0.88	0.45
***P8***	**209230_s_at**	0.09	0.15	0.16	0.00
***PRDM1***	**217192_s_at**	0.53	0.57	0.55	0.53
***TRKA***	**208605_s_at**	0.66	0.77	0.75	0.63
***PRDM2***	**205277_at**	0.21	0.25	0.28	0.25
	**203057_s_at**	0.56	0.56	0.59	0.58
	**203056_s_at**	0.57	0.59	0.59	0.55
	**216433_s_at**	0.33	0.42	0.45	0.12
***MEIS1***	**204069_at**	0.88	0.90	0.90	0.86
***HOXA9***	**214651_s_at**	0.90	0.90	0.91	0.89
	**209905_at**	0.91	0.91	0.90	0.90
***HOXA7***	**206847_s_at**	0.80	0.77	0.80	0.89
	**206848_at**	0.01	0.02	0.02	0.04
***CEBPA***	**204039_at**	0.61	0.61	0.63	0.58
***GMCSF***	**210229_s_at**	0.20	0.32	0.10	0.15

**Table 3:** Overlap *R*(*A, B*) between sets of genes marked as differentially expressed after pre-processing with different methods. *p*- and *q*-values for the significance of difference in expression between samples from the AML dataset with recurrent FLT3 mutation and samples without recurrent FLT3 mutation were calculated. The numbers on the diagonal represents the number of probe sets marked as differentially expressed after application of each method.

		MAS	dChip	RMA	GCRMA
SAM *q*-values	MAS	3185			
	dChip	0.68	2973		
	RMA	0.72	0.72	3649	
	GCRMA	0.73	0.70	0.86	3650

t-test *p*-values	MAS	458			
	dChip	0.66	354		
	RMA	0.68	0.70	419	
	GCRMA	0.69	0.71	0.83	472

Wilcoxon test *p*-values	MAS	337			
	dChip	0.72	295		
	RMA	0.75	0.79	322	
	GCRMA	0.75	0.79	0.87	344

**Table 4:** CNS dataset, GLIO samples vs. others.

		MAS	dChip	RMA	GCRMA
SAM *q*-values	MAS	400			
	dChip	0.39	714		
	RMA	0.50	0.54	666	
	GCRMA	0.52	0.39	0.58	330

t-test *p*-values	MAS	224			
	dChip	0.36	303		
	RMA	0.43	0.51	159	
	GCRMA	0.47	0.37	0.55	123

Wilcoxon test *p*-values	MAS	17			
	dChip	0.41	17		
	RMA	0.46	0.29	18	
	GCRMA	0.45	0.26	0.5	14

**Table 5:** Confusion matrix of MAS and RMA clustering results. Clustering into *k* = 12 clusters was performed using *k*-means clustering, using correlation distance on 3000 probe sets. A cell at position (*i, j*) shows the number of samples assigned to cluster i on data pre-processed using MAS and to cluster j on data pre-processed using RMA. The Jaccard index between these two clusterings is 0.55.

		Number of samples in RMA clusters											
		1	2	3	4	5	6	7	8	9	10	11	12

Number of samples in MAS clusters	1	33	1	1	0	1	1	1	0	0	1	1	0
	2	0	33	0	0	1	0	0	0	0	0	2	0
	3	0	3	28	2	0	0	0	0	0	0	0	0
	4	0	0	7	24	0	1	0	1	0	0	0	0
	5	0	1	0	0	21	2	0	0	0	0	0	0
	6	0	0	1	3	0	21	0	0	0	0	0	0
	7	0	0	0	0	0	0	21	0	0	0	0	0
	8	0	0	0	1	0	0	0	18	0	0	0	0
	9	0	0	0	0	4	0	0	0	10	0	0	8
	10	0	0	0	0	0	0	0	0	1	10	0	0
	11	0	3	0	0	0	0	0	0	0	0	10	0
	12	0	0	1	0	0	0	0	0	7	0	0	0

**Table 6:** Performance of different classification algorithms: AML dataset, inv(16) problem. Mean test set error (standard deviation) over 100 random splits of the original data into a training set (90%) and a test set (10%). Error is defined as average error per class, i.e. corresponding to assuming a prior probability of occurrence of a class of 50%. Classifiers were trained for 10, 20, 50, 100, 250, 500 and 1000 probe sets selected by the variation filter; results shown here are for the number of probe sets resulting in the smallest average test set error over the four methods, indicated between brackets after the classifier name.

		MAS	dChip	RMA	GCRMA
Classifier	NC (50)	0.01 (0.02)	0.02 (0.02)	0.01 (0.02)	0.02 (0.03)
(number of probe sets used)	PAM (20)	0.01 (0.02)	0.01 (0.02)	0.01 (0.02)	0.01 (0.02)
	LIKNON (10)	0.02 (0.03)	0.02 (0.03)	0.02 (0.02)	0.02 (0.02)
	k-NN (50)	0.01 (0.02)	0.01 (0.02)	0.01 (0.02)	0.01 (0.02)
	SVC/P (10)	0.02 (0.03)	0.02 (0.02)	0.02 (0.03)	0.02 (0.03)
	SVC/RBF (10)	0.03 (0.02)	0.02 (0.02)	0.02 (0.02)	0.02 (0.02)

**Table 7:** AML dataset, FLT3 problem.

		MAS	dChip	RMA	GCRMA
Classifier	NC (10)	0.16 (0.06)	0.19 (0.09)	0.16 (0.08)	0.26 (0.08)
(number of probe sets used)	PAM (20)	0.14 (0.06)	0.14 (0.06)	0.14 (0.06)	0.14 (0.06)
	LIKNON (20)	0.12 (0.05)	0.11 (0.05)	0.12 (0.06)	0.13 (0.05)
	k-NN (200)	0.10 (0.05)	0.11 (0.05)	0.11 (0.06)	0.13 (0.06)
	SVC/P (20)	0.12 (0.05)	0.11 (0.05)	0.13 (0.06)	0.13 (0.05)
	SVC/RBF (100)	0.09 (0.05)	0.10 (0.05)	0.10 (0.05)	0.14 (0.05)

**Table 8:** AML dataset, CCR problem.

		MAS	dChip	RMA	GCRMA
Classifier	NC (1000)	0.34 (0.09)	0.34 (0.08)	0.33 (0.08)	0.35 (0.08)
(number of probe sets used)	PAM (10)	0.29 (0.06)	0.30 (0.06)	0.30 (0.05)	0.30 (0.06)
	LIKNON (20)	0.27 (0.08)	0.31 (0.07)	0.31 (0.08)	0.28 (0.07)
	k-NN (20)	0.28 (0.07)	0.29 (0.06)	0.30 (0.06)	0.29 (0.07)
	SVC/P (20)	0.28 (0.08)	0.31 (0.07)	0.31 (0.07)	0.28 (0.06)
	SVC/RBF (20)	0.28 (0.05)	0.30 (0.04)	0.30 (0.04)	0.29 (0.04)

**Table 9:** CNS dataset, MED problem.

		MAS	dChip	RMA	GCRMA
Classifier	NC (1000)	0.04 (0.09)	0.03 (0.09)	0.03 (0.08)	0.07 (0.13)
(number of probe sets used)	PAM (1000)	0.05 (0.10)	0.07 (0.13)	0.09 (0.14)	0.06 (0.13)
	LIKNON (1000)	0.10 (0.12)	0.06 (0.11)	0.10 (0.13)	0.18 (0.18)
	k-NN (500)	0.06 (0.11)	0.06 (0.12)	0.08 (0.12)	0.04 (0.10)
	SVC/P (1000)	0.09 (0.12)	0.05 (0.11)	0.04 (0.09)	0.07 (0.12)
	SVC/RBF (10)	0.17 (0.13)	0.16 (0.13)	0.17 (0.14)	0.14 (0.14)
