# Supplementary material for: The effect of oligonucleotide microarray data pre-processing on the analysis of patient-cohort studies
Source: BMC Bioinformatics. 2006 Mar 2;7:105. doi: 10.1186/1471-2105-7-105 (PMC1481623; doi:10.1186/1471-2105-7-105)
Supplement: Additional File 1 — Short description: Supplemental tables 1, 2, 3, 4, Supplemental figures 1, 2. [file 1471-2105-7-105-S1.doc]

# SUPPLEMENTARY DATA

**The effect of oligonucleotide microarray data pre-processing on the analysis of patient-cohort studies**

Roel G.W. Verhaak^1^, Frank J.T. Staal^2^, Peter J.M. Valk^1^, Bob Lowenberg^1^, Marcel J.T. Reinders^3^, Dick de Ridder^2.3^

1. Department of Hematology, Erasmus Medical Center, Rotterdam, The Netherlands
2. Department of Immunology, Erasmus Medical Center, Rotterdam, The Netherlands
3. Information and Communication Theory Group, Faculty of Electrical Engineering, Mathematics and Computer Science, Delft University of Technology, Delft, the Netherlands

**Corresponding author:**

Roel G.W. Verhaak

Department of Hematology, Erasmus University Medical Center, Rotterdam

P.O. Box 1738

3000 DR Rotterdam

The Netherlands

Phone: +31-10-4087057

Fax: +31-10-4089470

e-mail:r.verhaak@erasmusmc.nl

## Supplementary Table 1. Primers and probes used for RT-PCR experiments.

|  | Reverse primer | Forward primer | Probe |
| --- | --- | --- | --- |
| *MEIS1* | 5’-TGAGTCCCGTGTCTT-3’ | 5’-CCCAGCACAGGTGAC-3’ | 6-FAM - 5’-CGTGGCATCTTTCCCAAAGTAG - 3’TAMRA |
| *HOXA7* | 5’-TAGCGCGTGTAGGTCTG-3’ | 5’-GCCTCCTACGACCAAAA-3’ | 6-FAM 5’-TTTCCGCATCTACCCCTGGA-3’TAMRA |
| *PRDM2* | 5’-TATTTTCCTGGGATTTTTTCTTC-3’ | 5’-TCCTGGTCTGGTACAATGG-3’ | 6-FAM – 5’-CAAGCGGAGCTCCCCCAAGAG - 3’TAMRA |
| *PRDM1* | 5’-AAAGCAACTGGATGCGCTATG-3’ | 5’-CCTAAGAACGCCAACAGGAAA-3’ | 6-FAM – 5’-TTCACCACTTCATTGACGGCTTTA - 3’TAMRA |
| *CEBPA* | 5’-TCGGTGGACAAGAACAG-3’ | 5’-GCAGGCGGTCATTG-3’ | 6-FAM – 5’- TGGAGACGCAGCAGAAGGTG – 3’TAMRA |
| *EVI1* | 5’ -CTTCTTGACTAAAGCCCTTGGA-3’ | 5’- GTACTTGAGCCAGCTTCCAACA-3’ | 6-FAM - 5’ -TCTTAGACGAATTTTACAATGTGAAGTTCTGCATAGATG-3’TAMRA |
| *TRKA* | 5'-TTGGCATGAGCAGGGATATCT-3' | 5'-ACGGTACAGGATGCTCTCGG-3 |  |
| *HOXA9* | 5’-CAGTTGGCTGCTGGGTTAT-3’ | 5’- CCCACGCTTGACACTC-3’ |  |
| *GMCSF* | 5’-CTGGCCGGTCACTCCT-3’ | 5’-GCAGCCTCACCAAGCTCAA-3’ |  |
| *P8* | 5’-GCCTCATCTCCAGCTCTGTG-3’ | 5’-ATAGCCTGGCCCATTCCTAC-3’ |  |

## Supplementary Table 2A. Expression of *CEBPA*, probe set id 204039_at.

## *n* = 208

| **Sample** | **PCR** | **MAS** | **dChip** | **RMA** | **GCRMA** |
| --- | --- | --- | --- | --- | --- |
| 322 | 0.5 | 1265.8 | 1490.5 | 3354.3 | 3321.3 |
| 1174 | 8.8 | 625.6 | 755.5 | 1806.2 | 1692.5 |
| 1188 | 3.3 | 416.2 | 518.2 | 1228.7 | 987.4 |
| 1197 | 4.3 | 671.5 | 768.2 | 1662.5 | 1437.8 |
| 1299 | 11.5 | 1175.8 | 949.6 | 2631.9 | 2489.9 |
| 1316 | 30.7 | 2923.3 | 2197.0 | 6083.1 | 6536.7 |
| 1432 | 4.4 | 998.3 | 973.3 | 2299.4 | 2146.1 |
| 1448 | 5.4 | 378.2 | 466.0 | 1145.4 | 869.0 |
| 1551 | 2.7 | 717.0 | 824.3 | 2112.9 | 1931.6 |
| 1595 | 3.6 | 548.8 | 804.0 | 1793.0 | 1525.7 |
| 1747 | 8.5 | 916.3 | 1213.7 | 2988.2 | 2829.4 |
| 1766 | 19.0 | 502.1 | 752.5 | 1584.2 | 1411.6 |
| 2169 | 16.5 | 2012.5 | 1652.9 | 4752.8 | 4979.0 |
| 2170 | 9.4 | 652.1 | 840.4 | 1800.0 | 1650.7 |
| 2171 | 13.1 | 566.1 | 661.0 | 1589.3 | 1348.7 |
| 2172 | 4.5 | 670.9 | 882.8 | 1813.7 | 1765.8 |
| 2173 | 17.0 | 747.3 | 838.2 | 1867.8 | 1768.4 |
| 2174 | 2.6 | 776.9 | 898.6 | 2106.4 | 2011.7 |
| 2175 | 26.7 | 667.5 | 709.8 | 1651.0 | 1453.2 |
| 2176 | 25.5 | 1130.2 | 1394.5 | 3051.9 | 3018.6 |
| 2177 | 10.4 | 846.6 | 1171.9 | 2967.5 | 2700.4 |
| 2178 | 5.2 | 269.3 | 351.6 | 717.1 | 493.5 |
| 2179 | 20.5 | 1003.0 | 1265.0 | 2472.3 | 2298.8 |
| 2181 | 4.0 | 697.2 | 807.1 | 1791.6 | 1477.6 |
| 2182 | 67.9 | 689.3 | 910.1 | 1716.9 | 1501.0 |
| 2183 | 7.3 | 376.8 | 495.7 | 1058.2 | 781.0 |
| 2184 | 31.1 | 1553.2 | 1893.2 | 3247.1 | 3386.0 |
| 2185 | 7.4 | 626.4 | 965.1 | 1772.8 | 1621.0 |
| 2186 | 5.5 | 369.7 | 577.1 | 1299.3 | 1058.5 |
| 2187 | 3.0 | 317.8 | 418.7 | 916.5 | 685.2 |
| 2188 | 11.0 | 1082.5 | 1142.3 | 2853.0 | 2603.9 |
| 2189 | 2.9 | 486.0 | 624.0 | 1261.3 | 1000.4 |
| 2190 | 4.5 | 634.5 | 634.2 | 1657.2 | 1501.9 |
| 2191 | 3.5 | 349.1 | 423.2 | 1005.8 | 685.5 |
| 2192 | 118.2 | 2886.6 | 2608.9 | 6406.7 | 7539.8 |
| 2193 | 11.5 | 1040.4 | 1028.7 | 2502.6 | 2409.1 |
| 2194 | 9.3 | 1234.7 | 1403.8 | 3394.8 | 3441.2 |
| 2195 | 4.8 | 679.0 | 908.9 | 2090.4 | 1981.5 |
| 2196 | 10.7 | 730.4 | 888.0 | 1995.4 | 1761.7 |
| 2197 | 0.4 | 246.5 | 358.3 | 792.4 | 482.2 |
| 2198 | 3.6 | 680.8 | 884.4 | 1873.1 | 1623.6 |
| 2199 | 3.2 | 1718.1 | 1604.7 | 4222.5 | 4472.5 |
| 2200 | 0.5 | 229.7 | 194.8 | 400.3 | 290.5 |
| 2201 | 6.3 | 704.7 | 763.4 | 1862.7 | 1811.8 |
| 2202 | 3.5 | 360.8 | 435.0 | 834.9 | 643.8 |
| 2203 | 4.2 | 629.1 | 806.1 | 1605.1 | 1442.3 |
| 2204 | 5.9 | 457.9 | 642.4 | 1185.4 | 990.5 |
| 2205 | 87.4 | 1154.2 | 978.0 | 2653.2 | 2427.2 |
| 2206 | 87.4 | 808.6 | 935.2 | 2137.2 | 1984.2 |
| 2207 | 2.6 | 700.1 | 838.0 | 1833.9 | 1609.8 |
| 2208 | 3.2 | 174.5 | 277.8 | 545.8 | 358.4 |
| 2209 | 34.9 | 469.4 | 605.9 | 1231.3 | 1032.0 |
| 2210 | 0.8 | 167.3 | 170.6 | 376.6 | 228.1 |
| 2211 | 0.2 | 362.7 | 452.5 | 837.5 | 596.0 |
| 2212 | 5.2 | 627.8 | 794.7 | 1607.6 | 1415.2 |
| 2215 | 10.6 | 693.1 | 734.3 | 1462.8 | 1326.7 |
| 2216 | 32.5 | 1177.9 | 1141.4 | 3141.2 | 3140.8 |
| 2217 | 12.5 | 1072.8 | 1119.5 | 2559.3 | 2529.7 |
| 2218 | 66.3 | 2039.7 | 1777.1 | 4964.3 | 5341.6 |
| 2219 | 6.8 | 1237.1 | 1236.3 | 2872.9 | 2770.2 |
| 2220 | 4.8 | 388.5 | 531.2 | 1097.4 | 824.8 |
| 2222 | 1.3 | 321.9 | 309.6 | 917.6 | 767.5 |
| 2223 | 1.8 | 687.0 | 811.0 | 1858.7 | 1802.2 |
| 2224 | 0.5 | 268.2 | 372.8 | 985.8 | 714.2 |
| 2225 | 7.5 | 641.6 | 751.2 | 1937.1 | 1671.8 |
| 2226 | 7.2 | 584.9 | 644.3 | 1435.9 | 1180.1 |
| 2227 | 6.2 | 535.6 | 653.4 | 1387.1 | 1190.3 |
| 2228 | 3.9 | 659.7 | 707.2 | 1676.7 | 1447.2 |
| 2229 | 5.5 | 790.3 | 811.8 | 1860.0 | 1756.3 |
| 2230 | 17.6 | 1982.5 | 1665.5 | 4413.2 | 4734.8 |
| 2231 | 5.8 | 502.3 | 728.1 | 1442.1 | 1210.5 |
| 2233 | 21.5 | 847.8 | 933.1 | 2375.1 | 2238.9 |
| 2234 | 111.0 | 2857.2 | 2354.1 | 6839.5 | 7533.3 |
| 2235 | 4.9 | 509.9 | 605.9 | 1430.3 | 1191.6 |
| 2236 | 6.2 | 1032.8 | 1046.2 | 2353.5 | 2194.0 |
| 2237 | 65.3 | 2894.9 | 3189.4 | 7386.7 | 8910.9 |
| 2238 | 3.6 | 118.1 | 135.2 | 339.6 | 175.0 |
| 2239 | 4.5 | 566.7 | 699.4 | 1631.6 | 1465.0 |
| 2240 | 13.6 | 2670.1 | 2207.4 | 6156.0 | 6813.5 |
| 2241 | 4.2 | 687.2 | 857.2 | 2056.1 | 1907.0 |
| 2242 | 5.5 | 2235.8 | 1825.6 | 4901.7 | 5031.7 |
| 2243 | 1.5 | 275.6 | 371.1 | 813.5 | 567.5 |
| 2244 | 5.6 | 600.7 | 803.2 | 1512.3 | 1341.4 |
| 2246 | 1.8 | 672.3 | 888.6 | 2217.9 | 1904.0 |
| 2247 | 3.3 | 613.8 | 787.1 | 1781.0 | 1557.3 |
| 2248 | 12.6 | 1831.2 | 2462.5 | 4558.1 | 4655.5 |
| 2249 | 0.7 | 293.1 | 433.5 | 1087.8 | 831.6 |
| 2250 | 7.5 | 809.6 | 921.6 | 2260.0 | 1929.3 |
| 2251 | 0.7 | 149.6 | 93.8 | 292.0 | 194.9 |
| 2252 | 6.1 | 474.1 | 493.4 | 1088.2 | 855.9 |
| 2253 | 34.7 | 2159.3 | 1800.8 | 4940.2 | 5359.6 |
| 2254 | 16.0 | 521.9 | 577.7 | 1425.1 | 1105.7 |
| 2255 | 1.0 | 418.7 | 591.0 | 1220.1 | 973.8 |
| 2256 | 0.9 | 344.1 | 461.1 | 1026.1 | 777.9 |
| 2257 | 1.9 | 443.1 | 606.0 | 1468.6 | 1199.2 |
| 2259 | 10.5 | 467.2 | 649.0 | 1176.0 | 940.7 |
| 2260 | 5.3 | 471.2 | 591.1 | 1388.2 | 1143.6 |
| 2261 | 4.6 | 829.7 | 767.9 | 2049.1 | 1710.3 |
| 2262 | 5.7 | 345.8 | 508.3 | 1171.4 | 922.9 |
| 2263 | 48.0 | 1381.8 | 1455.4 | 3181.1 | 3151.7 |
| 2265 | 11.9 | 2258.6 | 2517.3 | 5301.7 | 5516.0 |
| 2266 | 153.8 | 546.6 | 692.3 | 1434.6 | 1261.7 |
| 2267 | 27.0 | 585.8 | 699.8 | 1695.1 | 1489.5 |
| 2268 | 10.7 | 855.1 | 895.7 | 2049.9 | 1879.6 |
| 2270 | 2.1 | 612.8 | 736.8 | 1579.6 | 1306.8 |
| 2271 | 3.6 | 820.9 | 860.9 | 1992.0 | 1800.8 |
| 2272 | 2.9 | 488.4 | 653.1 | 1686.8 | 1475.1 |
| 2273 | 4.2 | 3230.0 | 3573.5 | 7966.6 | 8870.0 |
| 2274 | 2.4 | 402.3 | 403.4 | 966.2 | 705.0 |
| 2275 | 5.2 | 706.6 | 764.1 | 1718.1 | 1500.8 |
| 2276 | 1.0 | 136.5 | 270.2 | 525.3 | 349.4 |
| 2278 | 1.9 | 470.0 | 436.5 | 974.6 | 781.1 |
| 2279 | 2.8 | 306.4 | 372.2 | 646.9 | 451.7 |
| 2280 | 13.2 | 866.4 | 1181.4 | 2506.1 | 2369.3 |
| 2281 | 4.4 | 629.5 | 752.1 | 1737.7 | 1434.9 |
| 2282 | 6.5 | 652.9 | 697.3 | 1654.1 | 1432.2 |
| 2283 | 1.0 | 359.5 | 501.2 | 967.1 | 792.9 |
| 2284 | 2.5 | 463.9 | 602.0 | 1304.7 | 1084.1 |
| 2285 | 3.1 | 1300.7 | 1087.4 | 3181.0 | 3010.2 |
| 2286 | 5.1 | 865.0 | 797.7 | 2209.4 | 2062.3 |
| 2287 | 2.0 | 639.8 | 655.3 | 1818.0 | 1565.8 |
| 2288 | 2.5 | 641.7 | 773.0 | 1799.7 | 1598.7 |
| 2289 | 1.6 | 491.6 | 655.0 | 1445.8 | 1184.9 |
| 2290 | 13.1 | 1266.6 | 1428.2 | 3288.3 | 3208.3 |
| 2291 | 6.8 | 1027.4 | 1028.1 | 2327.3 | 2107.8 |
| 2292 | 2.4 | 875.7 | 894.7 | 2291.0 | 2138.7 |
| 2293 | 3.4 | 884.3 | 863.8 | 2061.4 | 1873.7 |
| 2297 | 8.4 | 1181.4 | 1527.5 | 3106.2 | 3052.3 |
| 2299 | 1.1 | 653.1 | 630.2 | 1636.2 | 1442.4 |
| 2300 | 19.5 | 1432.2 | 1840.3 | 3829.6 | 4098.0 |
| 2301 | 2.0 | 502.6 | 509.6 | 1342.4 | 1086.6 |
| 2302 | 13.7 | 1153.1 | 1219.7 | 2677.6 | 2695.7 |
| 2304 | 0.0 | 9.5 | 14.0 | 137.8 | 62.6 |
| 2305 | 2.8 | 834.6 | 786.6 | 2125.4 | 2095.8 |
| 2306 | 2.2 | 951.1 | 898.9 | 2527.1 | 2207.2 |
| 2307 | 5.1 | 778.9 | 873.9 | 1875.6 | 1844.2 |
| 2326 | 3.1 | 1099.4 | 1237.7 | 3005.6 | 2913.2 |
| 2327 | 7.3 | 336.4 | 437.8 | 928.9 | 674.2 |
| 2466 | 39.1 | 890.3 | 987.9 | 2286.5 | 2230.8 |
| 2467 | 4.3 | 472.8 | 615.4 | 1207.8 | 971.7 |
| 2468 | 27.3 | 1236.7 | 1579.1 | 2801.1 | 3009.3 |
| 2480 | 6.1 | 660.9 | 971.8 | 1838.2 | 1552.9 |
| 2490 | 0.6 | 220.2 | 310.1 | 796.3 | 590.4 |
| 2497 | 2.7 | 274.4 | 244.1 | 486.3 | 347.9 |
| 2498 | 6.5 | 621.3 | 696.6 | 1500.2 | 1371.7 |
| 2507 | 6.2 | 943.2 | 911.2 | 2388.0 | 2293.5 |
| 2509 | 19.5 | 1430.1 | 1309.3 | 3268.2 | 3178.6 |
| 2510 | 7.2 | 693.2 | 683.8 | 1808.4 | 1738.0 |
| 2511 | 3.8 | 352.6 | 560.1 | 1014.9 | 813.2 |
| 2514 | 2.0 | 571.6 | 567.9 | 1623.7 | 1615.3 |
| 2516 | 59.9 | 1945.2 | 2646.6 | 4642.1 | 5069.1 |
| 2524 | 6.4 | 1187.8 | 838.3 | 2746.7 | 2687.6 |
| 2525 | 0.7 | 283.3 | 293.5 | 652.8 | 451.6 |
| 2534 | 11.5 | 1168.9 | 1239.0 | 2846.2 | 2732.9 |
| 2535 | 9.5 | 1806.8 | 2035.6 | 4205.9 | 4438.5 |
| 2541 | 4.2 | 781.1 | 745.6 | 1781.4 | 1542.6 |
| 2542 | 0.3 | 380.4 | 620.5 | 1119.7 | 847.8 |
| 2543 | 18.5 | 593.2 | 728.6 | 1578.1 | 1352.9 |
| 2544 | 16.5 | 1237.8 | 1181.6 | 2538.5 | 2419.0 |
| 2545 | 147.0 | 3276.6 | 2473.8 | 6839.0 | 7621.4 |
| 2549 | 2.3 | 339.1 | 432.1 | 998.3 | 781.8 |
| 2550 | 9.9 | 547.2 | 763.6 | 1754.6 | 1513.7 |
| 2551 | 1.8 | 275.9 | 275.5 | 684.3 | 461.4 |
| 2644 | 1.0 | 403.3 | 538.2 | 1115.0 | 996.2 |
| 2655 | 0.4 | 230.4 | 369.4 | 700.5 | 517.0 |
| 2656 | 0.4 | 401.6 | 634.0 | 1277.6 | 986.3 |
| 2658 | 0.1 | 34.7 | 53.4 | 167.9 | 66.1 |
| 2664 | 0.6 | 159.8 | 209.8 | 485.9 | 316.5 |
| 2665 | 3.9 | 854.6 | 962.4 | 2298.0 | 2023.6 |
| 2666 | 0.8 | 277.4 | 443.7 | 894.2 | 607.2 |
| 2668 | 0.0 | 8.2 | 6.4 | 110.1 | 16.7 |
| 2669 | 1.9 | 682.9 | 1001.2 | 1913.2 | 1677.8 |
| 2670 | 2.5 | 765.7 | 830.5 | 2010.9 | 1733.2 |
| 2671 | 5.4 | 925.9 | 977.6 | 2351.9 | 2140.9 |
| 2672 | 1.3 | 484.9 | 628.0 | 1351.7 | 1081.1 |
| 2676 | 0.9 | 335.8 | 458.8 | 894.1 | 676.3 |
| 2678 | 0.9 | 352.3 | 528.7 | 1042.2 | 799.4 |
| 2679 | 3.8 | 743.6 | 886.3 | 2282.2 | 2124.4 |
| 2680 | 2.3 | 584.9 | 862.6 | 1753.2 | 1474.4 |
| 2682 | 4.6 | 614.2 | 719.5 | 1564.8 | 1526.3 |
| 2683 | 2.6 | 815.1 | 1235.7 | 2484.2 | 2527.9 |
| 2685 | 8.9 | 188.2 | 243.2 | 546.9 | 328.9 |
| 2686 | 0.0 | 12.0 | 5.8 | 145.3 | 15.2 |
| 2687 | 10.3 | 521.7 | 726.8 | 1457.1 | 1153.7 |
| 2688 | 3.7 | 285.5 | 354.3 | 736.0 | 533.7 |
| 2689 | 8.9 | 420.3 | 562.4 | 1175.6 | 1006.5 |
| 2690 | 3.7 | 615.8 | 712.9 | 1525.4 | 1221.6 |
| 2692 | 4.8 | 584.5 | 672.5 | 1545.5 | 1339.9 |
| 2694 | 4.8 | 700.7 | 1124.6 | 2409.9 | 2445.2 |
| 2695 | 10.1 | 339.3 | 467.5 | 1046.5 | 783.8 |
| 2696 | 7.7 | 437.7 | 590.3 | 1400.8 | 1253.1 |
| 2747 | 2.1 | 246.1 | 392.3 | 790.4 | 585.4 |
| 2748 | 28.1 | 2517.7 | 2253.5 | 5669.7 | 5967.2 |
| 2749 | 4.8 | 1036.6 | 1033.4 | 2449.7 | 2567.1 |
| 2750 | 3.2 | 272.0 | 401.9 | 861.7 | 645.5 |
| 2751 | 4.1 | 243.8 | 330.7 | 673.6 | 488.4 |
| 2752 | 15.1 | 514.3 | 539.0 | 1392.2 | 1123.4 |
| 2753 | 69.3 | 2839.7 | 2771.0 | 6363.9 | 7979.0 |
| 2754 | 15.3 | 560.4 | 671.1 | 1428.0 | 1242.6 |
| 2756 | 9.7 | 517.2 | 700.7 | 1363.3 | 1170.2 |
| 2757 | 2.0 | 365.8 | 437.3 | 994.6 | 871.8 |
| 2762 | 0.9 | 192.0 | 141.2 | 383.3 | 208.4 |
| 2764 | 1.4 | 277.1 | 217.0 | 478.9 | 309.2 |
| 2765 | 7.7 | 791.2 | 1002.3 | 2098.4 | 1908.7 |
| 2766 | 5.3 | 745.0 | 762.2 | 1829.6 | 1785.5 |
| 2767 | 22.9 | 984.8 | 1024.6 | 2827.4 | 2895.2 |
| 2769 | 12.8 | 1166.1 | 1621.3 | 3376.9 | 3622.4 |
| 2771 | 2.4 | 560.7 | 907.3 | 1554.3 | 1333.7 |

## Supplementary Table 2B. Expression of *EVI1*, probe set id’s 215851_at and 221884_at.

*n* = 265

|  |  | **215851_at** | | | | **221884_at** | | | |
| --- | --- | --- | --- | --- | --- | --- | --- | --- | --- |
| **Sample** | **PCR** | **MAS** | **dChip** | **RMA** | **GCRMA** | **MAS** | **dChip** | **RMA** | **GCRMA** |
| 2655 | 0.6 | 7.9 | 19.8 | 19.7 | 4.1 | 2.8 | 98.8 | 22.4 | 6.1 |
| 322 | 1.0 | 8.2 | 31.8 | 18.9 | 4.2 | 1.7 | 81.9 | 21.7 | 6.1 |
| 1063 | 1.0 | 13.1 | 28.5 | 19.4 | 4.4 | 3.3 | 171.7 | 24.2 | 7.2 |
| 1174 | 1.0 | 11.6 | 35.0 | 18.7 | 4.2 | 1.4 | 125.4 | 20.7 | 6.4 |
| 1188 | 1.0 | 11.6 | 26.8 | 21.0 | 4.2 | 1.7 | 106.5 | 22.0 | 6.5 |
| 1197 | 1.0 | 6.2 | 16.6 | 20.5 | 4.3 | 8.1 | 211.3 | 26.3 | 6.9 |
| 1201 | 1.0 | 11.1 | 31.6 | 20.6 | 4.1 | 1.8 | 118.6 | 20.1 | 6.2 |
| 1299 | 1.0 | 11.9 | 20.2 | 21.4 | 4.3 | 0.8 | 112.4 | 22.0 | 6.8 |
| 1316 | 1.0 | 10.2 | 28.5 | 18.9 | 4.3 | 0.9 | 165.5 | 22.2 | 6.8 |
| 1401 | 1.0 | 8.2 | 34.4 | 18.5 | 4.1 | 6.9 | 129.8 | 20.4 | 6.1 |
| 1432 | 1.0 | 7.0 | 25.4 | 18.3 | 4.2 | 1.5 | 107.1 | 20.6 | 6.4 |
| 1448 | 1.0 | 4.6 | 23.2 | 17.4 | 4.3 | 1.9 | 156.9 | 24.9 | 7.3 |
| 1482 | 1.0 | 9.2 | 31.6 | 18.2 | 4.2 | 1.7 | 106.5 | 19.8 | 6.5 |
| 1551 | 1.0 | 8.6 | 18.2 | 17.7 | 4.1 | 3.1 | 71.2 | 22.0 | 6.1 |
| 1595 | 1.0 | 12.2 | 20.2 | 20.3 | 4.1 | 59.3 | 134.5 | 44.6 | 20.6 |
| 1747 | 1.0 | 3.9 | 24.2 | 17.9 | 3.9 | 1.8 | 106.1 | 19.3 | 5.6 |
| 1766 | 1.0 | 12.9 | 15.8 | 20.9 | 4.3 | 1.5 | 107.4 | 19.5 | 6.6 |
| 2169 | 1.0 | 10.3 | 27.5 | 19.2 | 4.2 | 2.8 | 132.8 | 22.2 | 6.3 |
| 2170 | 1.0 | 19.4 | 31.9 | 22.5 | 4.3 | 14.1 | 214.0 | 23.2 | 6.8 |
| 2171 | 1.0 | 8.6 | 17.8 | 18.1 | 4.0 | 2.3 | 91.6 | 21.2 | 6.0 |
| 2172 | 1.0 | 13.3 | 29.0 | 20.0 | 4.3 | 0.8 | 120.7 | 19.4 | 6.4 |
| 2173 | 1.0 | 19.6 | 23.1 | 21.4 | 4.4 | 2.7 | 114.4 | 22.7 | 7.0 |
| 2174 | 1.0 | 18.9 | 35.6 | 21.0 | 4.4 | 1.7 | 127.7 | 20.4 | 7.1 |
| 2175 | 1.0 | 7.5 | 24.5 | 20.3 | 4.2 | 2.1 | 113.0 | 20.7 | 5.9 |
| 2176 | 1.0 | 12.7 | 24.8 | 19.5 | 4.1 | 2.5 | 94.8 | 20.0 | 5.7 |
| 2177 | 1.0 | 10.7 | 22.6 | 18.8 | 4.0 | 1.7 | 101.5 | 21.4 | 5.5 |
| 2178 | 1.0 | 10.5 | 18.2 | 19.9 | 4.3 | 3.0 | 141.8 | 21.9 | 6.9 |
| 2179 | 1.0 | 3.9 | 13.4 | 17.6 | 4.1 | 1.0 | 125.6 | 20.2 | 6.0 |
| 2181 | 1.0 | 8.8 | 22.0 | 17.7 | 3.9 | 4.7 | 150.6 | 22.5 | 5.5 |
| 2182 | 1.0 | 10.5 | 25.6 | 19.0 | 4.3 | 1.9 | 227.4 | 22.2 | 6.4 |
| 2183 | 1.0 | 6.9 | 21.6 | 19.1 | 3.9 | 2.4 | 97.4 | 20.6 | 5.7 |
| 2184 | 1.0 | 8.4 | 22.9 | 20.4 | 4.2 | 0.6 | 83.1 | 21.3 | 6.2 |
| 2185 | 1.0 | 8.8 | 22.8 | 18.2 | 3.9 | 0.7 | 83.2 | 19.9 | 5.6 |
| 2187 | 1.0 | 13.1 | 24.2 | 21.6 | 4.2 | 7.2 | 112.5 | 22.5 | 6.2 |
| 2188 | 1.0 | 6.5 | 17.8 | 18.7 | 4.0 | 1.1 | 49.3 | 18.9 | 5.5 |
| 2189 | 1.0 | 1.8 | 15.2 | 17.9 | 4.1 | 0.6 | 132.3 | 19.9 | 6.1 |
| 2191 | 1.0 | 13.1 | 28.6 | 22.9 | 4.2 | 1.3 | 143.8 | 22.4 | 6.0 |
| 2192 | 1.0 | 13.6 | 23.8 | 20.8 | 4.3 | 3.7 | 158.8 | 22.6 | 6.8 |
| 2193 | 1.0 | 9.4 | 19.7 | 19.1 | 4.3 | 0.6 | 153.9 | 23.0 | 7.0 |
| 2194 | 1.0 | 11.0 | 26.0 | 21.4 | 4.2 | 2.1 | 168.1 | 20.1 | 6.2 |
| 2195 | 1.0 | 14.2 | 22.1 | 21.1 | 4.2 | 2.6 | 156.6 | 21.4 | 8.3 |
| 2197 | 1.0 | 11.5 | 28.3 | 19.5 | 4.2 | 0.6 | 158.2 | 20.3 | 6.3 |
| 2198 | 1.0 | 7.4 | 19.1 | 19.2 | 4.3 | 2.9 | 176.2 | 22.7 | 7.6 |
| 2199 | 1.0 | 25.2 | 26.5 | 22.7 | 4.4 | 13.9 | 130.2 | 23.9 | 7.2 |
| 2200 | 1.0 | 16.1 | 28.7 | 20.9 | 4.5 | 1.7 | 143.1 | 21.4 | 7.7 |
| 2201 | 1.0 | 15.0 | 27.3 | 20.8 | 4.3 | 3.7 | 259.8 | 21.2 | 6.8 |
| 2202 | 1.0 | 3.7 | 17.2 | 19.2 | 4.4 | 2.2 | 163.5 | 24.9 | 7.3 |
| 2203 | 1.0 | 19.1 | 29.8 | 23.6 | 4.5 | 1.5 | 195.9 | 21.2 | 7.5 |
| 2204 | 1.0 | 8.7 | 26.0 | 19.6 | 4.3 | 5.6 | 92.4 | 23.1 | 6.7 |
| 2205 | 1.0 | 11.2 | 25.8 | 18.8 | 4.2 | 2.7 | 175.7 | 20.8 | 6.3 |
| 2206 | 1.0 | 8.8 | 19.5 | 20.7 | 4.2 | 0.8 | 141.4 | 22.4 | 6.6 |
| 2208 | 1.0 | 10.1 | 26.9 | 19.8 | 4.2 | 2.6 | 157.9 | 22.7 | 6.7 |
| 2209 | 1.0 | 6.6 | 29.2 | 20.0 | 4.2 | 8.1 | 199.6 | 25.9 | 6.7 |
| 2210 | 1.0 | 2.8 | 10.1 | 20.2 | 4.5 | 2.0 | 205.7 | 26.6 | 7.8 |
| 2211 | 1.0 | 4.3 | 15.9 | 20.1 | 4.3 | 3.4 | 118.3 | 22.4 | 7.1 |
| 2212 | 1.0 | 13.4 | 27.0 | 19.5 | 4.3 | 5.9 | 218.6 | 21.9 | 6.5 |
| 2214 | 1.0 | 4.1 | 24.0 | 20.2 | 4.2 | 2.0 | 165.3 | 21.1 | 6.6 |
| 2215 | 1.0 | 10.8 | 28.5 | 20.8 | 4.3 | 2.4 | 172.3 | 24.1 | 6.8 |
| 2216 | 1.0 | 13.4 | 24.3 | 21.0 | 4.2 | 8.0 | 187.4 | 20.3 | 6.5 |
| 2217 | 1.0 | 7.3 | 32.2 | 17.8 | 3.9 | 1.6 | 168.6 | 24.9 | 5.9 |
| 2218 | 1.0 | 12.0 | 24.2 | 19.7 | 4.2 | 0.6 | 114.3 | 20.4 | 6.4 |
| 2219 | 1.0 | 10.6 | 19.8 | 18.1 | 4.2 | 10.5 | 106.6 | 23.7 | 6.5 |
| 2220 | 1.0 | 10.1 | 30.3 | 18.2 | 4.1 | 3.5 | 118.4 | 20.5 | 6.2 |
| 2223 | 1.0 | 13.7 | 26.3 | 19.5 | 4.3 | 11.7 | 191.1 | 25.6 | 7.2 |
| 2224 | 1.0 | 7.9 | 21.8 | 18.5 | 4.1 | 2.3 | 166.9 | 22.0 | 6.4 |
| 2225 | 1.0 | 9.8 | 30.4 | 18.7 | 3.9 | 1.3 | 111.4 | 19.4 | 5.8 |
| 2226 | 1.0 | 11.0 | 13.2 | 19.9 | 4.2 | 2.0 | 103.6 | 22.0 | 6.2 |
| 2227 | 1.0 | 19.2 | 37.8 | 25.3 | 4.7 | 1.2 | 136.7 | 22.1 | 8.5 |
| 2229 | 1.0 | 7.6 | 26.9 | 18.9 | 4.1 | 1.7 | 144.8 | 23.0 | 6.2 |
| 2230 | 1.0 | 8.1 | 21.6 | 18.7 | 4.3 | 10.3 | 134.6 | 23.9 | 6.8 |
| 2231 | 1.0 | 15.0 | 27.3 | 19.0 | 4.1 | 3.3 | 162.4 | 24.2 | 6.3 |
| 2233 | 1.0 | 6.5 | 21.8 | 18.8 | 4.0 | 0.8 | 158.8 | 19.6 | 5.9 |
| 2234 | 1.0 | 10.0 | 26.4 | 20.2 | 4.2 | 1.9 | 205.5 | 21.3 | 6.2 |
| 2235 | 1.0 | 8.8 | 24.4 | 18.9 | 4.1 | 1.9 | 101.9 | 21.5 | 6.2 |
| 2236 | 1.0 | 20.6 | 21.7 | 20.1 | 4.3 | 11.7 | 145.1 | 22.0 | 6.7 |
| 2237 | 1.0 | 14.3 | 34.4 | 20.0 | 4.2 | 1.0 | 108.6 | 20.6 | 5.8 |
| 2238 | 1.0 | 18.3 | 29.7 | 19.5 | 4.1 | 2.8 | 94.7 | 21.3 | 6.1 |
| 2239 | 1.0 | 13.6 | 28.6 | 19.0 | 4.2 | 1.5 | 118.1 | 20.3 | 6.2 |
| 2240 | 1.0 | 12.2 | 30.8 | 19.4 | 4.2 | 2.0 | 140.7 | 21.5 | 6.6 |
| 2241 | 1.0 | 10.2 | 21.9 | 17.9 | 4.0 | 1.6 | 128.5 | 20.8 | 5.6 |
| 2242 | 1.0 | 11.4 | 18.1 | 20.5 | 4.3 | 1.7 | 205.2 | 23.0 | 6.6 |
| 2243 | 1.0 | 13.0 | 28.2 | 18.8 | 4.3 | 1.7 | 200.4 | 21.5 | 6.5 |
| 2244 | 1.0 | 9.8 | 16.4 | 19.3 | 4.3 | 2.2 | 92.5 | 23.5 | 7.0 |
| 2245 | 1.0 | 5.2 | 20.6 | 19.0 | 4.2 | 2.5 | 123.2 | 21.4 | 6.4 |
| 2246 | 1.0 | 5.2 | 21.8 | 18.5 | 4.0 | 1.7 | 130.9 | 21.1 | 5.5 |
| 2247 | 1.0 | 7.3 | 21.8 | 18.8 | 4.1 | 6.4 | 150.5 | 20.4 | 6.3 |
| 2248 | 1.0 | 18.0 | 25.2 | 20.8 | 4.4 | 0.9 | 51.4 | 18.4 | 6.8 |
| 2249 | 1.0 | 6.3 | 40.3 | 21.7 | 4.3 | 4.6 | 137.5 | 22.4 | 6.6 |
| 2251 | 1.0 | 11.3 | 14.1 | 20.6 | 4.8 | 2.3 | 137.5 | 23.5 | 10.2 |
| 2252 | 1.0 | 8.0 | 33.7 | 19.1 | 4.3 | 10.0 | 135.3 | 22.1 | 6.8 |
| 2253 | 1.0 | 6.2 | 30.7 | 20.5 | 4.3 | 3.6 | 129.3 | 23.0 | 7.8 |
| 2254 | 1.0 | 12.7 | 29.2 | 19.3 | 4.2 | 1.5 | 136.7 | 20.8 | 6.2 |
| 2255 | 1.0 | 11.5 | 29.5 | 21.2 | 4.2 | 1.8 | 128.1 | 24.2 | 6.4 |
| 2256 | 1.0 | 7.4 | 21.3 | 19.2 | 4.2 | 2.3 | 151.3 | 22.0 | 6.6 |
| 2257 | 1.0 | 12.3 | 32.4 | 18.7 | 4.3 | 1.8 | 204.8 | 24.0 | 6.8 |
| 2259 | 1.0 | 8.5 | 17.9 | 20.4 | 4.1 | 3.0 | 67.5 | 21.6 | 6.3 |
| 2260 | 1.0 | 9.9 | 18.9 | 20.7 | 4.2 | 1.3 | 134.8 | 20.6 | 6.4 |
| 2261 | 1.0 | 6.5 | 26.3 | 18.5 | 4.0 | 1.1 | 119.8 | 20.4 | 5.8 |
| 2262 | 1.0 | 12.1 | 30.7 | 21.3 | 4.2 | 2.3 | 158.6 | 21.0 | 6.6 |
| 2263 | 1.0 | 6.3 | 16.1 | 17.6 | 4.2 | 1.3 | 114.0 | 19.4 | 6.3 |
| 2265 | 1.0 | 20.2 | 26.6 | 21.5 | 4.5 | 2.7 | 118.8 | 20.6 | 6.8 |
| 2266 | 1.0 | 13.6 | 33.2 | 22.7 | 4.7 | 3.2 | 185.8 | 26.4 | 8.3 |
| 2267 | 1.0 | 10.3 | 21.7 | 19.9 | 4.4 | 1.0 | 182.8 | 20.3 | 7.0 |
| 2268 | 1.0 | 16.3 | 27.0 | 22.0 | 4.6 | 7.4 | 148.8 | 24.6 | 8.7 |
| 2270 | 1.0 | 14.7 | 33.5 | 21.0 | 4.3 | 6.5 | 203.4 | 22.2 | 6.9 |
| 2271 | 1.0 | 11.6 | 30.0 | 19.5 | 4.1 | 2.1 | 125.6 | 21.7 | 6.3 |
| 2272 | 1.0 | 8.5 | 27.5 | 18.7 | 4.2 | 2.2 | 210.3 | 21.9 | 6.5 |
| 2273 | 1.0 | 14.8 | 24.2 | 21.7 | 4.3 | 1.8 | 268.6 | 20.8 | 6.7 |
| 2274 | 1.0 | 14.6 | 36.5 | 20.8 | 4.3 | 3.1 | 177.7 | 25.2 | 6.7 |
| 2275 | 1.0 | 4.3 | 28.7 | 20.7 | 4.3 | 8.3 | 169.9 | 25.1 | 7.8 |
| 2278 | 1.0 | 16.7 | 37.7 | 20.6 | 4.4 | 10.2 | 182.2 | 23.8 | 7.1 |
| 2279 | 1.0 | 5.5 | 30.1 | 20.1 | 4.3 | 1.8 | 161.8 | 22.2 | 7.0 |
| 2280 | 1.0 | 9.1 | 27.3 | 18.5 | 4.2 | 1.4 | 157.0 | 19.2 | 6.4 |
| 2281 | 1.0 | 6.5 | 24.8 | 18.7 | 4.1 | 1.1 | 176.7 | 19.8 | 6.0 |
| 2282 | 1.0 | 3.3 | 35.8 | 19.7 | 4.3 | 0.6 | 191.8 | 22.0 | 6.3 |
| 2283 | 1.0 | 13.9 | 36.5 | 22.6 | 4.7 | 7.7 | 195.4 | 24.6 | 8.8 |
| 2284 | 1.0 | 12.1 | 30.3 | 20.9 | 4.2 | 1.2 | 189.5 | 22.1 | 6.3 |
| 2285 | 1.0 | 9.5 | 19.9 | 18.9 | 4.1 | 2.3 | 159.3 | 25.2 | 6.5 |
| 2286 | 1.0 | 11.9 | 29.3 | 19.4 | 4.4 | 11.4 | 205.6 | 22.3 | 8.2 |
| 2287 | 1.0 | 11.0 | 31.0 | 18.9 | 4.2 | 1.7 | 157.1 | 22.9 | 6.5 |
| 2289 | 1.0 | 11.4 | 40.7 | 19.6 | 4.3 | 0.8 | 167.4 | 21.7 | 6.6 |
| 2290 | 1.0 | 10.1 | 32.2 | 20.1 | 4.2 | 2.0 | 208.3 | 21.1 | 6.5 |
| 2291 | 1.0 | 9.6 | 28.4 | 19.2 | 4.2 | 2.5 | 208.5 | 24.8 | 6.6 |
| 2292 | 1.0 | 14.0 | 19.0 | 20.6 | 4.3 | 2.5 | 267.9 | 22.8 | 6.9 |
| 2293 | 1.0 | 14.3 | 33.7 | 19.5 | 4.3 | 3.4 | 221.4 | 26.2 | 7.2 |
| 2296 | 1.0 | 11.4 | 21.5 | 20.3 | 4.3 | 2.3 | 161.0 | 24.6 | 6.8 |
| 2297 | 1.0 | 16.9 | 36.4 | 20.6 | 4.4 | 1.5 | 132.1 | 22.4 | 7.5 |
| 2299 | 1.0 | 22.3 | 34.5 | 22.7 | 4.4 | 2.0 | 238.9 | 22.7 | 7.2 |
| 2300 | 1.0 | 1.0 | 23.3 | 19.2 | 4.4 | 2.2 | 208.8 | 23.6 | 7.4 |
| 2301 | 1.0 | 8.4 | 26.6 | 20.7 | 4.2 | 1.4 | 146.8 | 21.5 | 6.4 |
| 2302 | 1.0 | 11.1 | 26.1 | 19.7 | 4.4 | 1.3 | 204.6 | 21.2 | 7.1 |
| 2304 | 1.0 | 29.6 | 36.6 | 22.9 | 4.6 | 3.3 | 214.4 | 25.7 | 8.1 |
| 2305 | 1.0 | 3.9 | 25.4 | 21.6 | 4.5 | 4.2 | 242.7 | 22.1 | 7.7 |
| 2306 | 1.0 | 4.2 | 26.3 | 20.0 | 4.1 | 1.9 | 147.8 | 21.7 | 6.3 |
| 2307 | 1.0 | 4.1 | 20.5 | 18.3 | 4.6 | 4.0 | 161.0 | 42.2 | 10.3 |
| 2466 | 1.0 | 20.8 | 29.7 | 20.2 | 4.6 | 11.9 | 151.4 | 25.9 | 9.0 |
| 2467 | 1.0 | 16.0 | 32.1 | 20.6 | 4.3 | 11.3 | 97.5 | 23.0 | 6.8 |
| 2468 | 1.0 | 18.5 | 32.5 | 23.3 | 4.9 | 1.1 | 269.9 | 22.1 | 9.1 |
| 2480 | 1.0 | 14.4 | 25.2 | 19.6 | 4.2 | 8.2 | 129.3 | 21.0 | 6.5 |
| 2490 | 1.0 | 5.7 | 14.8 | 17.8 | 4.4 | 1.2 | 163.6 | 22.2 | 7.3 |
| 2497 | 1.0 | 6.9 | 9.0 | 21.1 | 4.5 | 2.0 | 158.1 | 21.9 | 8.2 |
| 2498 | 1.0 | 17.9 | 24.4 | 22.2 | 4.6 | 14.7 | 147.5 | 25.0 | 10.8 |
| 2507 | 1.0 | 14.8 | 24.4 | 19.8 | 4.3 | 1.3 | 158.1 | 20.9 | 6.7 |
| 2509 | 1.0 | 10.2 | 18.3 | 19.8 | 4.5 | 2.4 | 131.1 | 24.9 | 7.9 |
| 2510 | 1.0 | 9.3 | 27.0 | 19.6 | 4.3 | 1.1 | 116.3 | 21.1 | 6.8 |
| 2511 | 1.0 | 20.4 | 22.8 | 20.0 | 4.2 | 11.9 | 112.6 | 22.9 | 6.4 |
| 2514 | 1.0 | 9.0 | 25.9 | 22.6 | 4.6 | 2.4 | 285.8 | 22.2 | 8.4 |
| 2516 | 1.0 | 17.6 | 36.0 | 24.1 | 4.5 | 13.2 | 177.2 | 23.7 | 7.5 |
| 2524 | 1.0 | 6.4 | 31.9 | 19.2 | 4.4 | 4.1 | 217.7 | 21.3 | 7.1 |
| 2525 | 1.0 | 14.2 | 30.7 | 21.5 | 4.4 | 2.8 | 264.3 | 24.0 | 8.5 |
| 2534 | 1.0 | 10.3 | 23.1 | 20.2 | 4.3 | 7.4 | 175.1 | 24.0 | 7.2 |
| 2535 | 1.0 | 15.4 | 40.6 | 18.5 | 4.3 | 0.8 | 163.1 | 19.9 | 6.8 |
| 2541 | 1.0 | 2.1 | 19.6 | 18.2 | 4.3 | 8.7 | 87.4 | 21.9 | 6.8 |
| 2543 | 1.0 | 11.7 | 23.3 | 19.8 | 4.2 | 2.9 | 121.1 | 21.0 | 6.8 |
| 2544 | 1.0 | 6.5 | 40.7 | 19.8 | 4.4 | 13.6 | 116.5 | 22.5 | 7.5 |
| 2545 | 1.0 | 10.4 | 30.4 | 20.7 | 4.4 | 2.6 | 118.5 | 23.0 | 7.2 |
| 2546 | 1.0 | 12.9 | 21.2 | 19.9 | 4.3 | 1.3 | 175.8 | 21.6 | 6.8 |
| 2549 | 1.0 | 15.7 | 24.2 | 22.4 | 4.3 | 12.9 | 159.6 | 22.0 | 6.8 |
| 2550 | 1.0 | 17.0 | 25.7 | 20.6 | 4.2 | 2.5 | 96.8 | 20.7 | 6.3 |
| 2644 | 1.0 | 28.2 | 28.7 | 22.3 | 4.6 | 3.7 | 123.6 | 21.5 | 7.7 |
| 2647 | 1.0 | 11.2 | 20.8 | 19.6 | 4.3 | 10.3 | 102.1 | 23.9 | 6.8 |
| 2656 | 1.0 | 12.1 | 31.7 | 18.7 | 4.1 | 2.3 | 128.8 | 20.0 | 6.1 |
| 2658 | 1.0 | 12.5 | 25.4 | 20.3 | 4.2 | 5.4 | 176.4 | 22.0 | 6.2 |
| 2665 | 1.0 | 15.3 | 31.1 | 20.2 | 4.2 | 1.2 | 128.0 | 19.8 | 6.1 |
| 2667 | 1.0 | 13.8 | 19.2 | 20.9 | 4.3 | 13.8 | 146.0 | 24.6 | 7.2 |
| 2668 | 1.0 | 16.2 | 33.0 | 21.3 | 4.3 | 3.6 | 181.7 | 20.6 | 6.5 |
| 2669 | 1.0 | 11.4 | 30.5 | 20.2 | 4.3 | 2.4 | 135.1 | 20.7 | 6.8 |
| 2670 | 1.0 | 9.5 | 28.2 | 17.8 | 4.0 | 1.3 | 98.1 | 19.1 | 5.7 |
| 2671 | 1.0 | 9.9 | 24.0 | 19.7 | 4.3 | 1.3 | 132.8 | 21.2 | 6.8 |
| 2672 | 1.0 | 5.5 | 27.5 | 19.4 | 4.2 | 1.2 | 148.6 | 20.7 | 6.3 |
| 2676 | 1.0 | 2.4 | 16.1 | 18.3 | 4.3 | 4.4 | 186.1 | 23.4 | 7.4 |
| 2678 | 1.0 | 9.3 | 16.6 | 20.5 | 4.1 | 0.8 | 95.2 | 19.8 | 6.2 |
| 2679 | 1.0 | 17.7 | 26.4 | 19.4 | 4.4 | 12.1 | 136.6 | 25.9 | 7.2 |
| 2680 | 1.0 | 15.6 | 20.0 | 19.7 | 4.3 | 2.1 | 91.2 | 19.7 | 6.5 |
| 2681 | 1.0 | 27.3 | 41.2 | 23.9 | 5.1 | 8.0 | 221.8 | 27.2 | 10.9 |
| 2683 | 1.0 | 32.2 | 30.9 | 24.2 | 4.7 | 2.3 | 150.8 | 25.7 | 9.2 |
| 2685 | 1.0 | 19.8 | 32.2 | 22.4 | 4.5 | 1.5 | 173.2 | 24.0 | 7.7 |
| 2686 | 1.0 | 15.9 | 17.4 | 21.3 | 4.3 | 13.4 | 124.2 | 21.0 | 7.1 |
| 2687 | 1.0 | 12.6 | 22.1 | 19.8 | 4.5 | 1.9 | 140.6 | 22.8 | 8.1 |
| 2688 | 1.0 | 12.9 | 29.4 | 21.6 | 4.9 | 1.6 | 303.9 | 21.1 | 10.4 |
| 2689 | 1.0 | 18.3 | 24.7 | 21.3 | 4.4 | 0.9 | 114.4 | 20.1 | 7.1 |
| 2690 | 1.0 | 18.7 | 32.6 | 24.6 | 4.8 | 10.6 | 224.8 | 23.7 | 8.6 |
| 2692 | 1.0 | 12.8 | 32.8 | 22.1 | 4.8 | 4.7 | 282.5 | 24.3 | 9.0 |
| 2694 | 1.0 | 4.1 | 21.6 | 21.0 | 4.5 | 13.8 | 143.5 | 22.6 | 7.7 |
| 2695 | 1.0 | 11.9 | 19.8 | 20.2 | 4.3 | 4.6 | 148.6 | 22.7 | 7.0 |
| 2696 | 1.0 | 29.3 | 27.3 | 21.2 | 4.4 | 2.1 | 164.0 | 23.9 | 7.6 |
| 2704 | 1.0 | 31.8 | 27.3 | 23.4 | 4.7 | 15.7 | 216.9 | 24.3 | 9.1 |
| 2748 | 1.0 | 5.7 | 18.3 | 19.3 | 4.1 | 7.8 | 117.5 | 20.2 | 6.4 |
| 2749 | 1.0 | 12.1 | 36.0 | 20.7 | 4.3 | 2.6 | 154.7 | 24.2 | 6.9 |
| 2750 | 1.0 | 9.3 | 34.5 | 19.3 | 4.4 | 2.7 | 148.8 | 22.2 | 7.2 |
| 2751 | 1.0 | 15.3 | 27.4 | 23.2 | 4.4 | 2.9 | 143.8 | 22.3 | 7.0 |
| 2752 | 1.0 | 6.3 | 29.2 | 18.8 | 4.0 | 1.4 | 64.3 | 21.0 | 5.9 |
| 2753 | 1.0 | 19.6 | 29.6 | 23.8 | 4.3 | 0.8 | 156.1 | 19.4 | 6.5 |
| 2754 | 1.0 | 15.6 | 35.2 | 22.4 | 4.5 | 7.0 | 120.4 | 23.1 | 7.6 |
| 2756 | 1.0 | 1.4 | 12.6 | 20.8 | 4.3 | 1.9 | 73.0 | 21.1 | 6.8 |
| 2757 | 1.0 | 9.0 | 13.1 | 19.1 | 4.5 | 1.9 | 214.0 | 21.7 | 8.3 |
| 2762 | 1.0 | 21.2 | 35.7 | 21.6 | 4.7 | 1.5 | 215.4 | 23.5 | 7.9 |
| 2764 | 1.0 | 13.6 | 28.2 | 21.9 | 4.6 | 10.8 | 237.1 | 24.3 | 8.2 |
| 2765 | 1.0 | 12.8 | 23.6 | 18.8 | 4.2 | 1.5 | 123.8 | 22.2 | 6.6 |
| 2766 | 1.0 | 9.8 | 23.3 | 20.1 | 4.2 | 1.4 | 217.6 | 21.8 | 6.7 |
| 2767 | 1.0 | 2.9 | 22.0 | 20.4 | 4.3 | 1.6 | 142.1 | 22.4 | 6.8 |
| 2769 | 1.0 | 16.7 | 15.9 | 21.2 | 4.5 | 1.5 | 221.3 | 22.5 | 7.9 |
| 2771 | 1.0 | 8.7 | 15.1 | 21.6 | 4.2 | 7.4 | 89.9 | 20.6 | 6.6 |
| 2773 | 1.0 | 24.8 | 30.2 | 19.0 | 4.3 | 2.5 | 109.2 | 23.2 | 6.8 |
| 2774 | 1.0 | 8.9 | 24.1 | 19.3 | 4.0 | 7.7 | 138.8 | 21.0 | 6.0 |
| 3096 | 1.0 | 16.6 | 36.8 | 20.6 | 4.2 | 1.9 | 137.1 | 22.1 | 6.3 |
| 3097 | 1.0 | 6.3 | 24.7 | 18.7 | 4.1 | 4.8 | 95.9 | 23.2 | 6.4 |
| 3098 | 1.0 | 13.3 | 24.0 | 21.5 | 4.4 | 2.6 | 133.8 | 20.8 | 7.3 |
| 3099 | 1.0 | 2.7 | 40.2 | 22.0 | 4.8 | 4.1 | 198.4 | 20.4 | 8.3 |
| 3100 | 1.0 | 14.3 | 30.0 | 22.0 | 4.2 | 1.6 | 136.7 | 21.7 | 6.0 |
| 3101 | 1.0 | 11.2 | 18.3 | 19.2 | 4.0 | 2.7 | 137.7 | 21.8 | 6.1 |
| 3308 | 1.0 | 6.1 | 21.0 | 19.0 | 4.1 | 1.6 | 100.0 | 20.2 | 6.0 |
| 3310 | 1.0 | 43.3 | 17.1 | 25.0 | 4.8 | 8.2 | 103.4 | 33.1 | 10.9 |
| 3311 | 1.0 | 15.0 | 28.8 | 19.4 | 4.3 | 8.2 | 166.4 | 21.9 | 6.7 |
| 3312 | 1.0 | 10.7 | 15.4 | 19.6 | 4.2 | 0.9 | 141.9 | 19.2 | 6.2 |
| 3313 | 1.0 | 21.4 | 21.3 | 23.8 | 4.7 | 1.2 | 188.4 | 23.1 | 8.0 |
| 3314 | 1.0 | 8.4 | 16.1 | 17.7 | 4.1 | 0.6 | 106.3 | 20.6 | 6.0 |
| 3317 | 1.0 | 12.7 | 22.9 | 19.6 | 4.2 | 1.8 | 153.2 | 21.8 | 6.6 |
| 3318 | 1.0 | 17.3 | 27.2 | 21.7 | 4.2 | 83.4 | 215.6 | 121.7 | 161.8 |
| 3319 | 1.0 | 13.0 | 30.3 | 20.0 | 4.5 | 2.7 | 179.1 | 20.8 | 7.3 |
| 3320 | 1.0 | 9.3 | 25.4 | 19.5 | 4.3 | 3.4 | 67.5 | 21.9 | 6.8 |
| 3321 | 1.0 | 7.8 | 9.9 | 19.4 | 4.1 | 1.5 | 82.5 | 19.5 | 6.2 |
| 3322 | 1.0 | 11.9 | 19.5 | 20.2 | 4.3 | 2.4 | 101.7 | 22.2 | 6.8 |
| 3323 | 1.0 | 25.8 | 24.3 | 21.9 | 4.3 | 51.2 | 149.0 | 48.8 | 72.7 |
| 3324 | 1.0 | 16.9 | 27.6 | 19.2 | 4.0 | 2.2 | 98.8 | 19.1 | 5.8 |
| 3325 | 1.0 | 19.2 | 26.9 | 19.6 | 4.2 | 2.5 | 120.4 | 19.9 | 6.6 |
| 3327 | 1.0 | 5.0 | 7.7 | 20.6 | 4.4 | 3.7 | 110.0 | 21.7 | 7.2 |
| 3329 | 1.0 | 19.4 | 31.1 | 20.2 | 4.4 | 4.7 | 101.9 | 22.0 | 6.9 |
| 3330 | 1.0 | 18.2 | 13.2 | 22.1 | 4.3 | 5.9 | 117.1 | 20.7 | 6.6 |
| 3331 | 1.0 | 4.5 | 17.5 | 17.4 | 4.2 | 4.9 | 73.8 | 21.0 | 6.4 |
| 3332 | 1.0 | 10.9 | 29.9 | 18.4 | 4.2 | 6.1 | 157.2 | 21.3 | 6.7 |
| 3333 | 1.0 | 1.8 | 17.8 | 18.0 | 4.4 | 9.5 | 61.2 | 22.0 | 7.1 |
| 3334 | 1.0 | 6.6 | 25.0 | 19.4 | 4.1 | 4.6 | 107.1 | 19.8 | 6.0 |
| 3481 | 1.0 | 4.2 | 1.1 | 18.1 | 4.3 | 2.9 | 128.8 | 21.2 | 6.9 |
| 3482 | 1.0 | 4.8 | 22.2 | 19.8 | 4.3 | 1.4 | 95.8 | 22.8 | 6.7 |
| 3483 | 1.0 | 15.4 | 29.7 | 20.6 | 4.3 | 3.8 | 85.8 | 20.7 | 6.7 |
| 3485 | 1.0 | 15.3 | 8.2 | 21.6 | 4.5 | 1.0 | 245.9 | 22.2 | 8.0 |
| 3486 | 1.0 | 12.2 | 32.3 | 22.7 | 4.5 | 2.6 | 152.0 | 23.1 | 7.6 |
| 3488 | 1.0 | 16.0 | 31.5 | 21.4 | 4.3 | 1.6 | 121.4 | 20.6 | 6.3 |
| 3489 | 1.0 | 8.8 | 17.9 | 18.5 | 4.2 | 2.4 | 117.7 | 22.9 | 6.3 |
| 3490 | 1.0 | 8.8 | 20.0 | 18.9 | 4.6 | 11.8 | 121.2 | 23.0 | 8.1 |
| 3491 | 1.0 | 6.9 | 20.8 | 19.1 | 4.1 | 0.7 | 154.9 | 22.1 | 6.2 |
| 3492 | 1.0 | 4.2 | 27.1 | 20.6 | 4.4 | 3.4 | 93.8 | 22.7 | 7.2 |
| 3493 | 1.0 | 14.4 | 27.2 | 19.6 | 4.3 | 1.3 | 115.4 | 21.5 | 6.8 |
| 2551 | 141.0 | 31.8 | 38.0 | 29.0 | 4.8 | 49.5 | 185.2 | 59.0 | 24.5 |
| 2222 | 149.0 | 12.2 | 23.0 | 19.4 | 4.4 | 18.6 | 169.1 | 29.8 | 9.4 |
| 2250 | 178.5 | 13.5 | 28.6 | 21.5 | 4.2 | 102.0 | 1203.5 | 178.3 | 273.7 |
| 2186 | 307.6 | 14.4 | 24.8 | 19.8 | 4.0 | 71.7 | 129.2 | 68.0 | 62.1 |
| 3328 | 1333.0 | 75.9 | 61.1 | 62.5 | 36.2 | 239.0 | 319.6 | 290.1 | 590.6 |
| 2661 | 1428.0 | 16.8 | 25.8 | 21.9 | 4.1 | 71.0 | 116.8 | 71.1 | 51.3 |
| 2326 | 2436.0 | 33.8 | 47.4 | 33.1 | 7.3 | 172.7 | 2212.4 | 242.4 | 459.1 |
| 2327 | 2512.7 | 17.3 | 29.9 | 24.4 | 4.2 | 149.0 | 1666.8 | 215.7 | 354.7 |
| 2207 | 3082.7 | 13.2 | 27.3 | 20.5 | 4.3 | 219.9 | 2209.9 | 278.6 | 439.3 |
| 2196 | 3456.3 | 39.2 | 75.1 | 40.9 | 16.9 | 212.2 | 2125.9 | 297.3 | 530.2 |
| 2228 | 3821.0 | 15.1 | 37.0 | 21.6 | 4.2 | 84.6 | 262.2 | 129.8 | 133.6 |
| 2288 | 3916.0 | 75.7 | 93.7 | 75.6 | 105.4 | 435.9 | 3147.3 | 620.6 | 1182.6 |
| 2276 | 4420.0 | 23.4 | 39.7 | 26.9 | 5.2 | 107.9 | 305.0 | 144.2 | 220.1 |
| 2682 | 4803.9 | 48.9 | 41.1 | 44.1 | 12.3 | 302.8 | 347.3 | 327.6 | 801.7 |
| 3102 | 9541.0 | 24.6 | 37.2 | 29.7 | 4.2 | 156.6 | 233.5 | 236.9 | 375.8 |
| 2190 | 12119.1 | 214.4 | 196.2 | 284.9 | 324.1 | 2.9 | 259.6 | 23.0 | 7.2 |
| 2666 | 16844.6 | 57.3 | 65.5 | 81.2 | 56.1 | 460.0 | 447.6 | 803.1 | 1614.4 |
| 2772 | 17560.0 | 37.2 | 39.9 | 28.6 | 5.9 | 146.7 | 214.2 | 160.3 | 230.3 |
| 2664 | 19551.6 | 1013.9 | 664.5 | 1350.8 | 1841.2 | 8.7 | 469.8 | 23.9 | 7.7 |
| 2542 | 24919.7 | 25.3 | 37.4 | 31.4 | 4.3 | 249.7 | 355.2 | 391.4 | 669.4 |
| 2747 | 27554.5 | 44.4 | 55.6 | 50.9 | 22.0 | 388.7 | 487.7 | 625.1 | 1211.2 |

## Supplementary Table 2C. Expression of *MEIS1*, probe set id 204069_at.

*n* = 214

| **Sample** | **PCR** | **MAS** | **dChip** | **RMA** | **GCRMA** |
| --- | --- | --- | --- | --- | --- |
| 1316 | 0.0 | 0.7 | -1.7 | 27.1 | 5.0 |
| 2179 | 0.0 | 7.4 | 12.1 | 27.1 | 6.7 |
| 2197 | 0.0 | 10.7 | 4.1 | 31.7 | 6.8 |
| 2208 | 0.0 | 5.9 | 5.3 | 35.9 | 6.9 |
| 2218 | 0.0 | 15.8 | 9.8 | 26.3 | 4.2 |
| 2240 | 0.0 | 10.9 | 11.5 | 29.5 | 6.2 |
| 2267 | 0.0 | 4.7 | 7.3 | 31.0 | 7.1 |
| 2511 | 0.0 | 12.7 | 4.0 | 30.6 | 6.8 |
| 2549 | 0.0 | 8.8 | 15.7 | 35.7 | 6.9 |
| 2695 | 0.0 | 13.9 | 5.0 | 41.4 | 7.6 |
| 2752 | 0.0 | 6.7 | 2.1 | 30.7 | 6.7 |
| 2764 | 0.0 | 19.0 | 22.2 | 53.4 | 7.4 |
| 2767 | 0.0 | 14.1 | 13.8 | 40.7 | 7.0 |
| 2169 | 0.0 | 5.2 | 6.1 | 39.7 | 6.8 |
| 2178 | 0.0 | 15.2 | 11.7 | 39.5 | 7.1 |
| 2200 | 0.0 | 11.2 | 12.6 | 35.3 | 7.3 |
| 2204 | 0.0 | 7.7 | 6.0 | 43.0 | 4.5 |
| 2211 | 0.0 | 14.6 | 11.2 | 43.5 | 6.9 |
| 2219 | 0.0 | 11.1 | 8.4 | 25.6 | 4.4 |
| 2238 | 0.0 | 16.7 | 16.7 | 35.6 | 7.2 |
| 2242 | 0.0 | 10.9 | 0.9 | 35.5 | 4.3 |
| 2262 | 0.0 | 17.9 | 13.5 | 32.5 | 6.9 |
| 2509 | 0.0 | 11.6 | 9.7 | 54.4 | 7.3 |
| 2658 | 0.0 | 13.4 | 4.3 | 31.9 | 6.7 |
| 2668 | 0.0 | 19.4 | 10.8 | 31.9 | 6.9 |
| 2680 | 0.0 | 8.8 | 15.0 | 34.3 | 7.0 |
| 2753 | 0.0 | 8.5 | 5.7 | 43.1 | 6.9 |
| 2762 | 0.0 | 14.4 | 16.1 | 33.3 | 7.4 |
| 1432 | 0.0 | 254.7 | 518.8 | 475.7 | 610.1 |
| 2192 | 0.0 | 6.3 | 6.8 | 37.5 | 7.0 |
| 2237 | 0.0 | 11.0 | 3.8 | 32.9 | 6.8 |
| 2253 | 0.0 | 3.4 | 7.7 | 41.3 | 4.6 |
| 2686 | 0.0 | 19.2 | 4.3 | 39.6 | 4.7 |
| 2234 | 0.0 | 14.4 | 9.7 | 38.4 | 7.2 |
| 2297 | 0.0 | 11.4 | 4.7 | 42.8 | 7.7 |
| 2301 | 0.0 | 8.8 | 15.7 | 28.6 | 6.8 |
| 2466 | 0.0 | 9.8 | 6.3 | 29.7 | 5.8 |
| 2468 | 0.0 | 20.7 | 10.0 | 35.7 | 7.6 |
| 2534 | 0.0 | 20.9 | 12.6 | 33.4 | 7.1 |
| 2671 | 0.0 | 5.1 | 4.7 | 32.6 | 7.0 |
| 2230 | 0.0 | 10.7 | 6.5 | 29.8 | 7.0 |
| 2265 | 0.1 | 8.0 | 12.3 | 31.6 | 7.0 |
| 322 | 0.1 | 7.8 | 12.2 | 30.5 | 6.7 |
| 2170 | 0.1 | 16.9 | 16.2 | 34.2 | 7.0 |
| 2273 | 0.1 | 9.9 | 5.5 | 35.9 | 6.6 |
| 2545 | 0.1 | 11.3 | 8.6 | 27.7 | 7.1 |
| 2243 | 0.1 | 6.7 | 10.7 | 35.0 | 4.3 |
| 2266 | 0.1 | 14.8 | 18.7 | 45.1 | 8.5 |
| 2212 | 0.1 | 42.1 | 58.3 | 54.5 | 15.9 |
| 2497 | 0.1 | 12.8 | 9.9 | 34.1 | 5.3 |
| 2748 | 0.1 | 4.9 | 5.8 | 41.6 | 4.2 |
| 2263 | 0.1 | 13.0 | 11.8 | 36.4 | 6.8 |
| 2516 | 0.1 | 2.6 | 9.5 | 35.4 | 7.1 |
| 2751 | 0.1 | 7.4 | 14.2 | 42.2 | 7.1 |
| 2279 | 0.2 | 6.4 | 20.4 | 43.0 | 7.3 |
| 1448 | 0.2 | 32.7 | 69.4 | 56.1 | 30.6 |
| 2283 | 0.3 | 11.2 | 17.4 | 34.5 | 7.4 |
| 2250 | 0.3 | 11.4 | 7.0 | 31.3 | 6.6 |
| 2259 | 0.3 | 11.3 | 14.8 | 40.9 | 6.9 |
| 2210 | 0.3 | 12.7 | 7.4 | 35.7 | 7.3 |
| 2514 | 0.5 | 52.8 | 98.7 | 68.1 | 44.2 |
| 2251 | 0.7 | 30.8 | 35.0 | 44.4 | 8.7 |
| 2766 | 0.8 | 36.6 | 83.1 | 56.3 | 15.8 |
| 2289 | 0.9 | 30.4 | 73.8 | 52.4 | 15.9 |
| 2304 | 1.0 | 15.7 | 17.3 | 32.2 | 7.3 |
| 2247 | 1.1 | 27.5 | 58.9 | 46.9 | 11.3 |
| 1197 | 1.1 | 33.8 | 37.4 | 47.0 | 10.7 |
| 1201 | 1.1 | 71.5 | 194.7 | 140.5 | 89.1 |
| 2544 | 1.2 | 15.2 | 23.6 | 41.8 | 7.9 |
| 2307 | 1.2 | 24.2 | 15.2 | 39.6 | 6.2 |
| 2215 | 1.2 | 21.3 | 51.9 | 48.7 | 10.6 |
| 2256 | 1.2 | 62.9 | 147.6 | 100.1 | 73.8 |
| 2773 | 1.3 | 47.8 | 52.7 | 53.7 | 18.6 |
| 2223 | 1.3 | 30.8 | 54.0 | 51.0 | 16.2 |
| 2189 | 1.5 | 43.3 | 116.7 | 86.9 | 53.4 |
| 2694 | 1.6 | 80.6 | 154.6 | 102.4 | 56.9 |
| 2176 | 1.6 | 56.5 | 119.0 | 88.6 | 44.8 |
| 2678 | 2.0 | 52.8 | 99.4 | 84.6 | 42.1 |
| 2287 | 2.1 | 61.6 | 147.9 | 104.0 | 53.9 |
| 2171 | 2.3 | 37.3 | 96.9 | 72.3 | 41.1 |
| 2249 | 2.4 | 77.5 | 183.2 | 120.0 | 78.6 |
| 2696 | 2.5 | 46.0 | 70.1 | 67.6 | 21.2 |
| 2690 | 2.8 | 140.3 | 210.3 | 138.9 | 146.9 |
| 2551 | 2.9 | 97.3 | 128.4 | 101.7 | 71.2 |
| 2228 | 3.1 | 69.3 | 165.9 | 101.0 | 74.7 |
| 2186 | 3.2 | 54.9 | 104.8 | 77.3 | 39.2 |
| 2655 | 3.5 | 158.2 | 370.7 | 239.8 | 229.0 |
| 2260 | 3.6 | 55.4 | 129.4 | 87.2 | 64.2 |
| 1747 | 3.6 | 445.4 | 761.1 | 721.7 | 1035.5 |
| 1188 | 3.8 | 72.9 | 197.9 | 138.8 | 68.6 |
| 2209 | 3.8 | 354.4 | 630.0 | 640.9 | 747.8 |
| 2254 | 3.8 | 95.7 | 211.3 | 166.5 | 129.0 |
| 2194 | 3.9 | 74.8 | 140.7 | 98.3 | 64.6 |
| 2222 | 4.2 | 134.0 | 239.0 | 198.7 | 209.4 |
| 2656 | 4.3 | 152.0 | 365.8 | 266.6 | 285.0 |
| 2274 | 4.5 | 59.1 | 119.3 | 97.5 | 59.5 |
| 2765 | 4.7 | 126.9 | 209.3 | 166.8 | 149.3 |
| 2525 | 4.7 | 100.8 | 187.6 | 145.8 | 123.8 |
| 2244 | 4.8 | 64.4 | 159.5 | 116.8 | 94.2 |
| 2543 | 4.9 | 83.7 | 199.5 | 136.4 | 104.5 |
| 2290 | 5.0 | 73.3 | 154.4 | 113.2 | 82.2 |
| 2510 | 5.0 | 11.8 | 15.4 | 35.1 | 7.0 |
| 2749 | 5.1 | 182.8 | 336.0 | 287.3 | 302.8 |
| 2229 | 5.8 | 211.0 | 388.5 | 343.8 | 388.7 |
| 2305 | 5.9 | 89.7 | 139.1 | 126.7 | 79.1 |
| 1595 | 6.0 | 171.1 | 340.5 | 281.1 | 294.9 |
| 2224 | 6.8 | 163.8 | 334.0 | 293.7 | 283.0 |
| 2750 | 6.9 | 157.2 | 310.6 | 276.5 | 275.9 |
| 2490 | 7.0 | 284.8 | 407.1 | 347.0 | 402.3 |
| 2535 | 7.4 | 245.8 | 406.2 | 356.6 | 444.1 |
| 2252 | 7.8 | 122.4 | 273.8 | 201.1 | 197.6 |
| 2664 | 8.3 | 335.8 | 549.3 | 475.5 | 587.8 |
| 2507 | 8.9 | 270.8 | 489.2 | 416.8 | 511.1 |
| 2670 | 9.0 | 156.0 | 372.1 | 285.0 | 298.8 |
| 2235 | 9.1 | 172.9 | 327.0 | 288.6 | 307.0 |
| 2644 | 9.2 | 475.0 | 675.6 | 639.1 | 979.7 |
| 1174 | 9.7 | 86.5 | 154.6 | 123.9 | 96.3 |
| 2774 | 10.0 | 276.4 | 542.9 | 468.8 | 564.9 |
| 2666 | 10.0 | 219.0 | 529.2 | 416.2 | 507.4 |
| 2285 | 10.0 | 300.8 | 542.8 | 537.8 | 617.6 |
| 2233 | 10.1 | 81.4 | 203.3 | 141.4 | 106.1 |
| 2683 | 10.8 | 516.1 | 933.0 | 722.6 | 1153.4 |
| 2226 | 10.8 | 148.3 | 342.2 | 289.9 | 270.7 |
| 2757 | 11.0 | 237.8 | 507.4 | 397.7 | 516.7 |
| 1551 | 11.0 | 365.2 | 591.6 | 552.5 | 690.3 |
| 2669 | 11.4 | 212.1 | 486.2 | 375.3 | 426.3 |
| 2692 | 11.4 | 296.3 | 488.4 | 459.5 | 577.1 |
| 3333 | 11.5 | 242.9 | 494.0 | 476.8 | 563.5 |
| 2278 | 11.9 | 193.2 | 327.3 | 283.6 | 309.5 |
| 2172 | 12.0 | 130.5 | 269.7 | 229.2 | 241.1 |
| 2190 | 12.0 | 116.9 | 210.9 | 184.7 | 162.9 |
| 2291 | 12.4 | 164.2 | 354.7 | 277.4 | 315.2 |
| 2296 | 12.6 | 113.8 | 199.5 | 157.0 | 143.4 |
| 2524 | 12.7 | 300.4 | 405.6 | 383.6 | 460.6 |
| 2236 | 12.8 | 221.7 | 427.1 | 369.8 | 470.3 |
| 2665 | 12.9 | 309.7 | 573.6 | 492.4 | 556.4 |
| 2327 | 13.1 | 119.7 | 284.1 | 198.9 | 210.3 |
| 2676 | 13.4 | 336.0 | 673.9 | 582.9 | 696.6 |
| 2672 | 13.6 | 243.3 | 478.4 | 406.6 | 549.1 |
| 2225 | 14.1 | 184.6 | 390.4 | 335.4 | 342.2 |
| 2220 | 14.6 | 191.4 | 432.9 | 357.9 | 388.5 |
| 2276 | 14.7 | 142.5 | 291.6 | 228.6 | 241.9 |
| 2293 | 15.1 | 210.4 | 391.4 | 326.1 | 393.0 |
| 2306 | 15.2 | 339.2 | 555.0 | 586.2 | 736.8 |
| 2541 | 15.3 | 364.3 | 650.8 | 550.5 | 866.5 |
| 2185 | 15.4 | 125.2 | 272.6 | 212.1 | 219.5 |
| 2191 | 15.5 | 87.8 | 170.6 | 133.6 | 101.6 |
| 2747 | 15.5 | 323.0 | 662.9 | 552.9 | 772.3 |
| 2771 | 15.8 | 248.6 | 594.3 | 407.4 | 493.7 |
| 2227 | 15.9 | 115.7 | 271.2 | 194.7 | 177.7 |
| 2216 | 16.0 | 180.6 | 389.9 | 300.5 | 343.1 |
| 2202 | 16.2 | 71.8 | 114.0 | 72.4 | 59.6 |
| 2246 | 16.2 | 289.2 | 559.5 | 495.7 | 560.3 |
| 2187 | 16.6 | 169.1 | 388.8 | 250.8 | 287.8 |
| 2241 | 16.6 | 309.1 | 552.7 | 557.0 | 675.9 |
| 2181 | 17.0 | 254.3 | 457.0 | 410.6 | 533.6 |
| 2272 | 17.0 | 170.4 | 342.1 | 289.3 | 279.4 |
| 2467 | 17.2 | 133.2 | 278.8 | 213.5 | 210.8 |
| 2174 | 17.6 | 223.3 | 392.0 | 327.9 | 410.4 |
| 2275 | 18.2 | 220.4 | 480.5 | 445.3 | 465.4 |
| 2542 | 18.4 | 192.2 | 450.6 | 359.4 | 400.1 |
| 2271 | 18.8 | 333.1 | 543.7 | 534.0 | 642.4 |
| 2292 | 18.9 | 332.6 | 563.5 | 537.7 | 634.8 |
| 2239 | 19.0 | 216.3 | 432.7 | 390.4 | 450.6 |
| 2231 | 19.1 | 164.5 | 416.2 | 270.7 | 340.3 |
| 2326 | 19.2 | 363.8 | 643.0 | 595.5 | 777.5 |
| 2182 | 19.4 | 99.1 | 275.3 | 185.0 | 162.5 |
| 2261 | 20.0 | 289.2 | 523.4 | 574.0 | 727.8 |
| 2687 | 20.1 | 125.9 | 302.0 | 198.5 | 170.1 |
| 2248 | 20.7 | 396.4 | 719.3 | 664.2 | 907.1 |
| 2284 | 21.6 | 376.8 | 666.4 | 545.0 | 753.6 |
| 2688 | 21.7 | 339.2 | 658.4 | 484.1 | 646.3 |
| 2217 | 22.6 | 163.3 | 369.9 | 322.1 | 350.9 |
| 2177 | 22.7 | 236.2 | 472.7 | 428.5 | 490.2 |
| 2282 | 22.9 | 187.3 | 371.6 | 348.3 | 379.2 |
| 2184 | 22.9 | 268.1 | 472.2 | 470.4 | 562.4 |
| 2257 | 23.2 | 388.8 | 632.5 | 668.4 | 814.1 |
| 2302 | 23.8 | 97.6 | 239.4 | 138.9 | 134.3 |
| 2270 | 24.1 | 354.3 | 578.7 | 556.5 | 719.1 |
| 2480 | 24.7 | 202.2 | 412.0 | 353.7 | 350.4 |
| 2300 | 25.6 | 283.7 | 669.0 | 395.4 | 547.7 |
| 2188 | 25.6 | 202.5 | 415.7 | 303.0 | 328.9 |
| 2679 | 25.6 | 415.8 | 693.2 | 822.9 | 1129.1 |
| 2255 | 28.1 | 560.6 | 931.4 | 996.2 | 1331.7 |
| 2268 | 28.6 | 351.2 | 574.3 | 525.5 | 645.0 |
| 2756 | 30.9 | 264.1 | 536.8 | 356.8 | 493.0 |
| 2772 | 31.2 | 325.7 | 622.5 | 474.1 | 553.7 |
| 1063 | 32.7 | 393.9 | 842.0 | 671.6 | 915.9 |
| 1299 | 33.5 | 316.4 | 541.6 | 532.4 | 649.7 |
| 2498 | 33.6 | 296.4 | 472.4 | 424.4 | 527.2 |
| 2195 | 34.3 | 308.6 | 543.9 | 571.8 | 696.2 |
| 2281 | 34.9 | 565.4 | 993.1 | 1056.6 | 1532.8 |
| 2689 | 35.0 | 244.8 | 463.1 | 386.5 | 478.2 |
| 2769 | 36.1 | 480.2 | 802.2 | 718.2 | 1232.9 |
| 2175 | 40.1 | 210.4 | 386.1 | 344.6 | 404.9 |
| 2183 | 40.4 | 359.7 | 646.8 | 622.9 | 804.5 |
| 2286 | 41.1 | 285.1 | 571.2 | 534.4 | 643.7 |
| 1766 | 41.4 | 403.7 | 831.7 | 796.1 | 1033.4 |
| 2280 | 42.0 | 288.8 | 558.4 | 443.5 | 598.7 |
| 2299 | 42.7 | 454.8 | 650.1 | 617.6 | 789.7 |
| 2754 | 42.7 | 276.0 | 467.8 | 428.9 | 475.2 |
| 2550 | 44.2 | 219.4 | 412.2 | 412.6 | 450.5 |
| 2288 | 48.2 | 628.1 | 888.5 | 986.9 | 1522.2 |
| 2685 | 49.7 | 299.5 | 690.4 | 461.9 | 582.5 |
| 2199 | 53.8 | 362.3 | 715.9 | 639.7 | 773.4 |
| 2198 | 58.9 | 219.3 | 434.6 | 419.9 | 464.9 |
| 2205 | 61.2 | 188.2 | 353.3 | 314.2 | 326.3 |
| 2193 | 62.5 | 293.6 | 500.4 | 486.3 | 615.2 |
| 2201 | 62.5 | 322.8 | 441.2 | 356.5 | 494.6 |
| 2206 | 69.6 | 207.8 | 421.1 | 360.1 | 384.1 |
| 2173 | 73.8 | 451.7 | 827.0 | 882.8 | 1277.5 |
| 2196 | 73.8 | 303.4 | 550.2 | 491.2 | 615.4 |
| 2207 | 84.7 | 389.8 | 681.9 | 607.0 | 884.9 |
| 2203 | 188.1 | 770.2 | 1228.8 | 1136.0 | 1849.4 |

## Supplementary Table 2D. Expression of *HOXA7*, probe set id’s 206847_s_at and 206848_at.

*n* = 213

|  |  | **206847_s_at** | | | | **206848_at** | | | |
| --- | --- | --- | --- | --- | --- | --- | --- | --- | --- |
| **Sample** | **PCR** | **MAS** | **dChip** | **RMA** | **GCRMA** | **MAS** | **dChip** | **RMA** | **GCRMA** |
| 1316 | 0.0 | 3.2 | -5.4 | 69.2 | 24.3 | 80.3 | 174.0 | 210.0 | 233.2 |
| 2179 | 0.0 | 2.2 | -8.0 | 63.9 | 18.4 | 96.4 | 203.4 | 221.1 | 252.7 |
| 2197 | 0.0 | 2.3 | -5.5 | 55.6 | 19.4 | 107.0 | 191.9 | 245.9 | 282.8 |
| 2200 | 0.0 | 3.4 | -5.5 | 70.0 | 23.3 | 144.5 | 227.4 | 281.8 | 385.1 |
| 2211 | 0.0 | 6.5 | -15.4 | 67.5 | 22.6 | 75.5 | 170.8 | 190.8 | 222.5 |
| 2218 | 0.0 | 10.0 | -14.6 | 72.4 | 20.7 | 139.2 | 255.0 | 313.6 | 387.1 |
| 2230 | 0.0 | 12.0 | -22.8 | 65.5 | 24.2 | 103.3 | 221.9 | 267.9 | 345.7 |
| 2234 | 0.0 | 1.9 | -25.4 | 75.5 | 17.7 | 131.6 | 248.2 | 319.2 | 421.9 |
| 2235 | 0.0 | 3.0 | -5.8 | 59.1 | 17.1 | 66.6 | 144.4 | 191.3 | 174.9 |
| 2240 | 0.0 | 1.5 | -10.4 | 63.4 | 22.4 | 76.3 | 167.4 | 173.4 | 194.3 |
| 2242 | 0.0 | 3.1 | -39.2 | 77.5 | 24.0 | 123.9 | 218.3 | 286.7 | 379.4 |
| 2259 | 0.0 | 2.9 | -12.6 | 64.1 | 22.3 | 81.8 | 164.8 | 181.1 | 203.8 |
| 2263 | 0.0 | 5.0 | -43.2 | 81.9 | 22.4 | 191.8 | 292.5 | 412.4 | 558.8 |
| 2266 | 0.0 | 3.7 | -35.1 | 77.7 | 26.0 | 229.5 | 273.4 | 352.1 | 495.0 |
| 2267 | 0.0 | 26.7 | -2.4 | 70.8 | 24.4 | 87.9 | 159.7 | 213.4 | 229.3 |
| 2273 | 0.0 | 3.2 | -7.2 | 70.1 | 22.8 | 121.4 | 209.7 | 266.9 | 321.1 |
| 2287 | 0.0 | 1.8 | -1.5 | 66.7 | 13.8 | 43.7 | 109.3 | 103.9 | 98.2 |
| 2293 | 0.0 | 3.8 | -9.1 | 62.9 | 24.3 | 142.5 | 244.9 | 333.5 | 458.7 |
| 2301 | 0.0 | 7.0 | -18.4 | 73.6 | 18.0 | 93.6 | 174.1 | 213.0 | 255.1 |
| 2468 | 0.0 | 15.2 | -37.7 | 83.8 | 25.3 | 130.3 | 214.7 | 276.6 | 429.1 |
| 2511 | 0.0 | 9.7 | -12.7 | 54.7 | 22.3 | 180.4 | 270.4 | 324.1 | 454.3 |
| 2680 | 0.0 | 2.7 | -12.1 | 51.3 | 18.4 | 78.8 | 145.7 | 152.7 | 136.5 |
| 2695 | 0.0 | 12.2 | -7.6 | 50.4 | 24.2 | 241.4 | 334.4 | 624.9 | 897.0 |
| 2748 | 0.0 | 10.9 | -7.6 | 59.7 | 22.5 | 155.1 | 271.6 | 326.1 | 391.0 |
| 2750 | 0.0 | 5.0 | -14.9 | 65.4 | 24.0 | 89.2 | 190.9 | 215.2 | 257.8 |
| 2751 | 0.0 | 2.3 | -11.0 | 61.3 | 21.7 | 168.5 | 283.9 | 337.0 | 456.6 |
| 2752 | 0.0 | 1.6 | -2.1 | 59.2 | 16.6 | 81.9 | 168.8 | 205.1 | 224.3 |
| 2762 | 0.0 | 8.9 | -4.4 | 66.3 | 25.3 | 72.0 | 139.1 | 161.2 | 176.5 |
| 2767 | 0.0 | 4.8 | -14.5 | 59.7 | 20.4 | 99.8 | 192.6 | 242.1 | 302.0 |
| 1448 | 0.0 | 28.3 | 29.9 | 88.8 | 29.6 | 31.9 | 88.0 | 87.6 | 65.5 |
| 2204 | 0.0 | 2.4 | -16.7 | 66.2 | 24.0 | 133.8 | 245.2 | 302.1 | 393.6 |
| 2219 | 0.0 | 15.5 | -21.7 | 68.1 | 22.4 | 195.5 | 318.0 | 451.7 | 612.6 |
| 2297 | 0.0 | 1.9 | -19.3 | 75.8 | 23.3 | 228.6 | 313.4 | 460.2 | 681.0 |
| 2534 | 0.0 | 9.0 | -15.3 | 78.8 | 24.3 | 154.6 | 273.3 | 382.0 | 525.2 |
| 2549 | 0.0 | 3.3 | -5.1 | 57.1 | 24.1 | 139.2 | 235.3 | 304.8 | 398.3 |
| 2678 | 0.0 | 10.3 | 0.4 | 53.7 | 23.8 | 81.8 | 186.6 | 196.1 | 248.0 |
| 2753 | 0.0 | 14.8 | -19.5 | 56.0 | 22.6 | 220.0 | 351.7 | 529.8 | 745.7 |
| 2764 | 0.0 | 11.5 | 10.5 | 65.6 | 24.7 | 220.0 | 330.4 | 483.8 | 725.1 |
| 2208 | 0.0 | 1.7 | -10.7 | 62.2 | 22.5 | 83.4 | 160.4 | 201.6 | 201.8 |
| 2249 | 0.0 | 19.8 | 3.8 | 82.0 | 25.0 | 76.8 | 168.7 | 202.4 | 207.5 |
| 2253 | 0.0 | 4.1 | -12.9 | 72.0 | 23.0 | 119.1 | 259.6 | 294.8 | 406.4 |
| 2262 | 0.0 | 2.4 | -10.7 | 60.0 | 23.4 | 174.5 | 292.3 | 381.6 | 473.2 |
| 2497 | 0.0 | 2.5 | -55.4 | 84.2 | 24.7 | 174.0 | 252.1 | 336.5 | 455.1 |
| 2671 | 0.0 | 2.0 | -6.3 | 66.2 | 25.1 | 109.8 | 243.6 | 303.1 | 381.4 |
| 2189 | 0.0 | 1.6 | -17.2 | 51.5 | 19.8 | 108.3 | 210.7 | 272.9 | 303.1 |
| 2192 | 0.0 | 2.5 | -26.0 | 72.9 | 22.8 | 118.8 | 202.6 | 260.1 | 332.7 |
| 2510 | 0.0 | 13.1 | -42.8 | 85.5 | 24.2 | 89.9 | 152.7 | 200.9 | 214.0 |
| 2516 | 0.0 | 4.1 | -28.6 | 74.2 | 24.3 | 330.2 | 473.5 | 690.4 | 1070.9 |
| 2658 | 0.0 | 13.9 | 0.4 | 67.6 | 23.1 | 80.2 | 166.3 | 204.6 | 209.5 |
| 1197 | 0.1 | 3.3 | -12.9 | 75.5 | 24.2 | 110.5 | 187.2 | 224.8 | 301.5 |
| 2184 | 0.1 | 1.2 | -8.9 | 58.4 | 21.6 | 96.9 | 214.3 | 266.8 | 282.4 |
| 2202 | 0.1 | 2.5 | -12.0 | 68.9 | 25.6 | 97.2 | 158.8 | 182.4 | 210.0 |
| 2169 | 0.1 | 0.9 | -12.1 | 67.6 | 17.5 | 94.0 | 159.8 | 216.1 | 234.3 |
| 2509 | 0.1 | 22.9 | -33.3 | 97.1 | 27.3 | 130.2 | 198.9 | 280.6 | 376.8 |
| 2666 | 0.1 | 1.8 | -11.6 | 66.1 | 17.1 | 149.0 | 264.0 | 334.1 | 423.6 |
| 2170 | 0.1 | 5.7 | -3.7 | 57.2 | 24.0 | 167.2 | 269.2 | 413.5 | 531.8 |
| 2265 | 0.1 | 8.2 | -7.6 | 63.0 | 21.4 | 179.1 | 294.5 | 419.5 | 572.2 |
| 2307 | 0.1 | 8.5 | -17.3 | 95.0 | 26.4 | 223.2 | 256.9 | 349.4 | 502.4 |
| 2187 | 0.2 | 2.1 | -6.1 | 58.5 | 19.9 | 139.3 | 293.3 | 382.9 | 528.1 |
| 2466 | 0.2 | 14.6 | -27.8 | 76.4 | 26.1 | 112.7 | 207.8 | 285.2 | 362.6 |
| 2178 | 0.2 | 5.2 | -4.4 | 61.4 | 22.8 | 171.8 | 251.9 | 316.0 | 448.2 |
| 2304 | 0.2 | 5.2 | -33.2 | 87.5 | 24.5 | 192.8 | 275.7 | 361.7 | 583.8 |
| 2237 | 0.3 | 2.8 | -8.2 | 57.6 | 16.9 | 131.1 | 251.2 | 333.2 | 432.5 |
| 2210 | 0.4 | 4.1 | -11.5 | 65.1 | 24.4 | 86.5 | 162.7 | 212.3 | 246.2 |
| 2243 | 0.5 | 2.8 | -27.4 | 81.3 | 23.4 | 144.5 | 256.3 | 303.4 | 423.1 |
| 322 | 0.6 | 2.6 | -6.8 | 54.4 | 19.8 | 56.6 | 114.1 | 122.0 | 89.7 |
| 2696 | 0.7 | 10.9 | 3.2 | 67.6 | 23.2 | 117.0 | 242.1 | 284.5 | 411.3 |
| 2251 | 0.8 | 27.7 | -71.3 | 117.2 | 38.1 | 92.2 | 102.5 | 158.0 | 189.4 |
| 2283 | 1.0 | 5.3 | -36.1 | 85.5 | 24.7 | 87.6 | 155.0 | 196.9 | 205.3 |
| 2766 | 1.0 | 3.0 | -2.8 | 71.3 | 22.6 | 95.2 | 212.6 | 264.5 | 337.3 |
| 2542 | 1.3 | 1.5 | -7.8 | 67.1 | 17.4 | 112.4 | 227.9 | 255.2 | 294.0 |
| 2305 | 1.5 | 20.1 | -6.9 | 88.8 | 25.6 | 95.5 | 165.2 | 195.0 | 242.6 |
| 2217 | 1.9 | 1.7 | -1.1 | 68.1 | 15.3 | 58.3 | 136.1 | 157.4 | 147.1 |
| 2664 | 2.2 | 26.5 | 10.9 | 74.8 | 24.6 | 218.5 | 329.8 | 456.8 | 652.2 |
| 2656 | 2.3 | 16.1 | 6.8 | 61.7 | 17.3 | 127.5 | 261.1 | 322.6 | 396.8 |
| 2176 | 2.3 | 6.9 | -4.5 | 60.4 | 15.5 | 91.6 | 195.1 | 233.1 | 261.4 |
| 2186 | 2.6 | 13.6 | 16.1 | 55.9 | 15.5 | 92.4 | 220.0 | 256.7 | 317.1 |
| 2279 | 2.7 | 8.9 | -3.2 | 81.4 | 24.2 | 64.6 | 146.1 | 176.8 | 165.9 |
| 2545 | 3.4 | 8.5 | 4.7 | 67.0 | 23.4 | 174.0 | 289.8 | 372.1 | 532.2 |
| 2280 | 3.6 | 2.0 | -11.8 | 66.8 | 20.3 | 152.5 | 267.0 | 338.5 | 437.5 |
| 2256 | 5.3 | 33.7 | -17.7 | 93.7 | 24.1 | 88.4 | 168.3 | 218.9 | 223.9 |
| 2215 | 5.6 | 3.8 | 10.7 | 77.8 | 22.7 | 81.1 | 169.7 | 208.5 | 255.7 |
| 2228 | 6.5 | 8.8 | -3.5 | 74.7 | 20.3 | 40.1 | 108.8 | 104.2 | 92.9 |
| 1201 | 6.5 | 16.5 | 64.7 | 93.6 | 38.1 | 98.4 | 185.4 | 217.8 | 251.0 |
| 2749 | 7.0 | 35.4 | 36.7 | 94.1 | 30.2 | 94.7 | 190.1 | 244.2 | 306.2 |
| 2467 | 7.1 | 33.3 | 102.4 | 122.3 | 51.7 | 97.8 | 197.8 | 241.3 | 293.6 |
| 2254 | 7.3 | 9.1 | 10.4 | 63.5 | 23.9 | 61.3 | 133.9 | 169.1 | 153.7 |
| 2274 | 7.6 | 2.0 | -1.2 | 74.1 | 23.2 | 117.8 | 210.7 | 280.3 | 351.7 |
| 2490 | 7.7 | 63.9 | 53.4 | 133.5 | 66.6 | 52.2 | 96.6 | 127.7 | 106.2 |
| 2290 | 7.9 | 31.3 | 18.3 | 93.4 | 24.9 | 174.4 | 261.7 | 338.0 | 490.6 |
| 2543 | 8.1 | 15.0 | 2.7 | 77.5 | 24.2 | 148.8 | 256.2 | 302.8 | 397.3 |
| 2551 | 8.3 | 48.4 | 37.8 | 93.6 | 29.1 | 137.8 | 266.2 | 346.5 | 502.3 |
| 2223 | 9.1 | 19.6 | -35.5 | 103.4 | 25.4 | 88.2 | 135.2 | 179.6 | 195.9 |
| 2212 | 9.7 | 24.1 | 17.1 | 91.5 | 24.3 | 44.4 | 101.7 | 110.6 | 83.4 |
| 2690 | 11.4 | 50.3 | 70.7 | 112.1 | 54.4 | 104.6 | 151.1 | 208.2 | 264.3 |
| 2514 | 12.8 | 26.2 | -15.3 | 95.8 | 26.0 | 88.5 | 150.3 | 195.3 | 194.7 |
| 2525 | 14.8 | 36.7 | 71.5 | 96.4 | 33.8 | 110.4 | 182.3 | 230.5 | 270.2 |
| 2498 | 16.5 | 70.6 | 73.3 | 98.3 | 38.5 | 147.7 | 227.3 | 311.2 | 445.3 |
| 2255 | 16.6 | 8.1 | 2.1 | 70.2 | 22.7 | 103.6 | 209.3 | 253.3 | 290.0 |
| 2747 | 17.2 | 13.2 | 4.2 | 76.1 | 22.3 | 107.2 | 182.4 | 239.6 | 259.7 |
| 2655 | 17.3 | 55.8 | 103.9 | 137.1 | 97.3 | 74.0 | 174.9 | 200.2 | 221.9 |
| 2171 | 18.8 | 9.5 | 4.1 | 59.9 | 14.2 | 61.2 | 134.8 | 154.2 | 148.6 |
| 2172 | 19.7 | 4.2 | -1.0 | 60.3 | 23.0 | 124.8 | 243.9 | 344.3 | 448.3 |
| 2276 | 19.8 | 19.0 | 32.6 | 89.2 | 25.8 | 124.3 | 207.2 | 242.4 | 292.7 |
| 2185 | 20.8 | 29.2 | 98.3 | 121.0 | 39.1 | 93.2 | 226.3 | 288.3 | 300.6 |
| 2289 | 21.5 | 33.4 | 49.3 | 97.3 | 26.9 | 72.9 | 158.9 | 158.7 | 171.9 |
| 2327 | 22.2 | 11.9 | 32.1 | 85.5 | 23.9 | 78.6 | 172.6 | 197.4 | 226.9 |
| 2260 | 22.4 | 19.8 | 45.8 | 91.4 | 31.4 | 89.2 | 192.0 | 242.6 | 273.4 |
| 2683 | 23.4 | 46.3 | 153.7 | 124.9 | 81.7 | 162.7 | 280.7 | 371.3 | 566.2 |
| 2773 | 23.8 | 41.9 | 13.3 | 76.8 | 24.2 | 139.3 | 232.1 | 266.2 | 363.6 |
| 2507 | 24.8 | 49.6 | 111.7 | 131.2 | 75.9 | 144.6 | 243.7 | 306.4 | 402.2 |
| 2668 | 26.9 | 35.4 | 27.7 | 86.7 | 27.8 | 283.6 | 435.3 | 715.5 | 1017.7 |
| 2544 | 27.3 | 32.5 | 23.1 | 104.2 | 30.5 | 158.9 | 244.9 | 300.0 | 394.8 |
| 2676 | 27.4 | 54.2 | 161.7 | 147.6 | 103.4 | 110.1 | 192.0 | 218.4 | 258.7 |
| 1595 | 27.6 | 27.2 | 27.1 | 83.3 | 24.6 | 79.8 | 154.8 | 160.2 | 149.0 |
| 2238 | 27.6 | 2.7 | -10.0 | 48.3 | 17.3 | 289.1 | 506.4 | 678.5 | 1199.9 |
| 2665 | 29.5 | 53.8 | 99.4 | 107.7 | 68.0 | 275.1 | 414.1 | 649.5 | 936.6 |
| 1063 | 31.1 | 47.5 | 114.3 | 126.6 | 56.1 | 203.1 | 330.2 | 447.4 | 617.7 |
| 2535 | 31.2 | 32.9 | 78.4 | 109.4 | 58.9 | 209.0 | 296.3 | 469.5 | 694.4 |
| 2672 | 32.9 | 65.2 | 161.9 | 160.0 | 127.7 | 126.4 | 193.2 | 256.8 | 313.0 |
| 2285 | 34.3 | 40.5 | 110.8 | 120.3 | 74.5 | 57.4 | 97.5 | 128.5 | 106.0 |
| 2524 | 34.7 | 65.6 | 155.0 | 186.9 | 134.8 | 91.2 | 163.7 | 192.8 | 213.4 |
| 2270 | 34.9 | 41.0 | 87.2 | 112.2 | 75.8 | 165.5 | 295.1 | 365.0 | 516.4 |
| 2688 | 36.6 | 55.9 | 139.2 | 144.1 | 60.6 | 272.5 | 330.7 | 464.4 | 789.5 |
| 2670 | 39.5 | 31.7 | 121.8 | 120.0 | 40.9 | 83.0 | 170.8 | 200.3 | 225.8 |
| 1188 | 40.8 | 18.3 | 61.8 | 103.9 | 37.6 | 60.3 | 140.7 | 137.7 | 154.5 |
| 2644 | 41.2 | 43.5 | 115.1 | 104.1 | 36.4 | 407.3 | 600.0 | 1139.1 | 1891.6 |
| 2687 | 41.9 | 22.6 | 55.0 | 94.9 | 24.4 | 224.5 | 345.8 | 422.4 | 635.7 |
| 2686 | 42.4 | 46.9 | 69.6 | 99.3 | 49.0 | 217.7 | 342.0 | 372.5 | 565.4 |
| 2550 | 43.9 | 27.1 | 22.7 | 73.8 | 22.7 | 154.0 | 273.4 | 364.6 | 514.8 |
| 2250 | 43.9 | 6.9 | -3.9 | 63.2 | 17.8 | 128.3 | 252.8 | 328.4 | 388.5 |
| 2541 | 45.3 | 50.7 | 144.2 | 152.8 | 106.3 | 61.9 | 132.0 | 149.6 | 153.7 |
| 2679 | 48.0 | 67.1 | 172.6 | 152.9 | 146.9 | 200.3 | 325.3 | 418.9 | 721.8 |
| 2224 | 51.1 | 33.6 | 68.5 | 110.4 | 53.9 | 49.7 | 94.8 | 128.7 | 107.7 |
| 2480 | 56.9 | 40.4 | 133.1 | 123.0 | 99.4 | 228.7 | 324.1 | 448.6 | 649.1 |
| 2685 | 57.5 | 40.4 | 104.6 | 136.1 | 96.7 | 116.6 | 265.7 | 322.4 | 478.6 |
| 2190 | 59.5 | 19.9 | 19.9 | 77.2 | 24.3 | 82.2 | 157.5 | 181.9 | 206.5 |
| 2757 | 61.0 | 48.0 | 29.8 | 85.7 | 35.3 | 158.8 | 221.3 | 261.7 | 410.3 |
| 2302 | 62.2 | 50.4 | 106.1 | 112.1 | 84.9 | 230.7 | 321.9 | 493.1 | 709.9 |
| 2306 | 63.3 | 54.9 | 204.3 | 141.6 | 116.6 | 141.5 | 285.6 | 378.8 | 496.5 |
| 2291 | 64.9 | 47.2 | 160.2 | 130.9 | 104.6 | 121.4 | 198.9 | 257.5 | 312.3 |
| 2261 | 66.3 | 33.4 | 130.0 | 112.0 | 36.3 | 109.1 | 237.7 | 303.8 | 345.8 |
| 2756 | 66.7 | 36.7 | 98.1 | 123.1 | 45.8 | 169.9 | 286.5 | 350.2 | 453.7 |
| 2257 | 67.0 | 42.9 | 138.1 | 144.6 | 40.4 | 85.9 | 167.5 | 208.0 | 205.0 |
| 2326 | 68.1 | 61.7 | 224.6 | 157.1 | 165.2 | 162.6 | 301.2 | 382.1 | 502.1 |
| 1747 | 68.4 | 54.6 | 231.3 | 158.0 | 125.4 | 157.3 | 301.9 | 405.8 | 520.7 |
| 2278 | 69.3 | 49.6 | 76.2 | 127.7 | 63.6 | 96.0 | 136.4 | 183.6 | 213.8 |
| 2244 | 72.5 | 25.3 | 82.4 | 132.6 | 32.9 | 39.5 | 112.8 | 121.1 | 105.9 |
| 2296 | 72.8 | 39.4 | 62.2 | 120.4 | 35.9 | 71.5 | 154.5 | 172.2 | 191.4 |
| 2288 | 74.5 | 41.7 | 135.0 | 144.4 | 76.0 | 106.1 | 201.8 | 241.1 | 309.9 |
| 2299 | 76.6 | 31.1 | 66.0 | 90.4 | 55.5 | 147.0 | 229.1 | 293.2 | 444.1 |
| 2233 | 82.4 | 16.3 | 26.7 | 90.3 | 23.6 | 83.7 | 153.4 | 199.2 | 195.3 |
| 2222 | 85.3 | 18.4 | -23.5 | 97.7 | 24.6 | 81.9 | 148.4 | 176.9 | 209.1 |
| 2292 | 87.1 | 53.2 | 119.1 | 139.8 | 61.9 | 162.3 | 250.9 | 330.7 | 439.6 |
| 2252 | 87.7 | 65.9 | 45.4 | 142.9 | 73.9 | 124.2 | 191.4 | 248.8 | 317.4 |
| 3333 | 96.7 | 87.0 | 181.8 | 262.7 | 164.6 | 61.3 | 157.5 | 170.5 | 173.1 |
| 2183 | 97.3 | 71.9 | 244.7 | 167.0 | 184.0 | 104.0 | 197.2 | 241.6 | 246.2 |
| 2229 | 97.3 | 45.0 | 138.6 | 157.3 | 105.6 | 101.4 | 204.8 | 242.9 | 303.1 |
| 2272 | 98.7 | 40.8 | 98.3 | 118.0 | 74.5 | 75.0 | 176.4 | 201.9 | 227.0 |
| 2247 | 99.4 | 48.2 | 122.5 | 149.0 | 81.6 | 134.2 | 235.7 | 305.4 | 357.4 |
| 2275 | 99.7 | 49.2 | 144.5 | 156.9 | 79.0 | 70.4 | 154.7 | 174.2 | 203.3 |
| 2689 | 100.8 | 63.6 | 176.5 | 129.6 | 138.6 | 288.4 | 432.3 | 594.9 | 935.7 |
| 2669 | 104.3 | 64.5 | 222.6 | 217.1 | 181.5 | 91.6 | 212.4 | 233.8 | 267.1 |
| 2268 | 107.3 | 66.6 | 68.4 | 110.8 | 50.8 | 246.7 | 374.4 | 511.3 | 809.5 |
| 2194 | 111.4 | 36.9 | 111.5 | 117.3 | 47.4 | 104.8 | 200.0 | 202.8 | 244.1 |
| 2774 | 113.4 | 46.6 | 150.3 | 127.0 | 92.0 | 94.5 | 205.9 | 246.9 | 287.8 |
| 2246 | 120.3 | 47.0 | 141.4 | 147.0 | 86.0 | 106.1 | 186.2 | 237.5 | 267.2 |
| 2281 | 120.7 | 98.3 | 288.1 | 281.4 | 280.9 | 144.4 | 257.9 | 340.7 | 427.9 |
| 2282 | 122.4 | 58.9 | 139.7 | 150.3 | 140.3 | 66.0 | 142.0 | 161.1 | 173.8 |
| 2271 | 124.1 | 74.9 | 201.1 | 146.3 | 139.0 | 109.2 | 211.4 | 243.4 | 287.7 |
| 2300 | 127.1 | 94.3 | 186.4 | 221.0 | 157.1 | 205.9 | 310.2 | 410.8 | 621.8 |
| 2284 | 142.0 | 74.3 | 175.9 | 199.3 | 148.2 | 118.5 | 203.9 | 270.0 | 316.3 |
| 2226 | 142.5 | 36.5 | 109.6 | 103.6 | 59.9 | 115.1 | 197.0 | 236.9 | 283.8 |
| 1432 | 152.8 | 52.1 | 153.2 | 164.4 | 129.8 | 87.8 | 185.4 | 226.0 | 255.7 |
| 2239 | 152.8 | 42.7 | 168.3 | 118.6 | 73.3 | 130.7 | 289.0 | 351.8 | 458.1 |
| 1551 | 155.4 | 34.7 | 140.5 | 101.1 | 47.6 | 53.8 | 66.5 | 101.9 | 84.6 |
| 2754 | 165.4 | 55.0 | 117.9 | 118.6 | 90.3 | 150.4 | 259.9 | 360.8 | 500.9 |
| 2286 | 167.2 | 45.1 | 150.2 | 134.0 | 109.8 | 91.1 | 166.2 | 198.1 | 229.6 |
| 2231 | 178.5 | 34.1 | 89.7 | 109.2 | 41.8 | 121.3 | 244.6 | 304.2 | 372.4 |
| 2241 | 179.2 | 57.6 | 177.6 | 157.4 | 121.2 | 200.1 | 327.0 | 482.0 | 615.6 |
| 2771 | 183.6 | 34.2 | 117.4 | 137.8 | 83.5 | 120.2 | 237.0 | 294.4 | 327.9 |
| 1174 | 200.9 | 29.5 | 90.0 | 131.4 | 52.4 | 116.5 | 176.6 | 223.0 | 272.6 |
| 2216 | 219.0 | 45.5 | 98.4 | 116.9 | 54.1 | 91.5 | 188.4 | 217.9 | 277.2 |
| 2248 | 225.2 | 23.4 | 138.5 | 126.4 | 85.4 | 145.1 | 257.7 | 352.0 | 418.9 |
| 2225 | 238.9 | 70.5 | 222.4 | 179.7 | 189.0 | 124.0 | 244.8 | 308.5 | 375.9 |
| 2199 | 239.7 | 49.9 | 106.8 | 119.1 | 83.3 | 119.0 | 220.2 | 283.6 | 393.1 |
| 2692 | 248.1 | 72.1 | 221.8 | 160.4 | 65.4 | 165.6 | 279.5 | 382.2 | 506.5 |
| 2182 | 267.8 | 41.6 | 100.1 | 112.7 | 44.0 | 158.2 | 264.2 | 311.1 | 370.0 |
| 2227 | 289.0 | 42.0 | 59.5 | 119.7 | 46.0 | 79.3 | 157.7 | 182.6 | 223.5 |
| 2236 | 294.1 | 51.7 | 179.0 | 133.7 | 136.2 | 101.7 | 211.6 | 234.8 | 292.8 |
| 2191 | 308.7 | 19.9 | 52.2 | 91.0 | 25.4 | 106.1 | 194.9 | 243.4 | 299.3 |
| 2205 | 344.9 | 43.3 | 124.8 | 126.8 | 69.1 | 78.9 | 159.3 | 194.2 | 201.8 |
| 2198 | 358.3 | 44.2 | 117.4 | 139.5 | 90.2 | 67.4 | 157.1 | 160.3 | 180.0 |
| 2175 | 360.8 | 43.3 | 115.1 | 116.6 | 70.6 | 56.7 | 116.5 | 147.4 | 124.7 |
| 1766 | 386.7 | 42.4 | 129.8 | 112.8 | 57.0 | 139.6 | 251.8 | 320.1 | 400.1 |
| 1299 | 423.1 | 52.7 | 236.2 | 167.9 | 194.0 | 143.3 | 235.0 | 314.4 | 376.1 |
| 2765 | 426.1 | 60.9 | 152.9 | 119.3 | 84.0 | 252.8 | 385.9 | 532.2 | 726.8 |
| 2195 | 444.2 | 59.0 | 148.5 | 134.9 | 92.2 | 83.6 | 196.8 | 229.1 | 296.5 |
| 2207 | 445.7 | 74.6 | 162.7 | 161.2 | 118.4 | 97.1 | 187.4 | 206.8 | 254.6 |
| 2188 | 447.3 | 21.9 | 78.9 | 95.2 | 24.2 | 140.2 | 285.4 | 373.3 | 464.1 |
| 2181 | 455.1 | 79.7 | 200.4 | 203.4 | 176.5 | 93.3 | 206.3 | 252.3 | 285.6 |
| 2203 | 456.7 | 57.5 | 163.1 | 170.9 | 73.6 | 201.2 | 303.3 | 389.4 | 485.1 |
| 2206 | 467.9 | 48.7 | 133.5 | 114.6 | 54.9 | 96.8 | 177.4 | 219.8 | 255.1 |
| 2220 | 491.1 | 63.6 | 262.9 | 231.4 | 198.6 | 64.8 | 159.4 | 192.0 | 202.3 |
| 2177 | 539.3 | 70.2 | 230.6 | 147.6 | 152.7 | 121.1 | 253.3 | 297.5 | 345.4 |
| 2193 | 558.3 | 56.7 | 152.9 | 149.7 | 106.8 | 114.6 | 217.1 | 263.0 | 322.6 |
| 2201 | 594.3 | 58.8 | 142.7 | 141.2 | 89.7 | 128.5 | 187.5 | 234.1 | 326.7 |
| 2772 | 608.9 | 40.9 | 83.7 | 107.6 | 48.8 | 95.1 | 173.7 | 195.0 | 211.8 |
| 2173 | 659.4 | 51.9 | 101.6 | 165.7 | 57.6 | 163.7 | 282.3 | 332.1 | 499.5 |
| 2174 | 680.3 | 69.6 | 142.8 | 175.6 | 120.9 | 88.2 | 163.1 | 195.8 | 243.2 |
| 2209 | 692.2 | 65.2 | 204.8 | 143.0 | 148.4 | 112.5 | 196.3 | 230.1 | 270.5 |
| 2769 | 716.6 | 84.0 | 205.1 | 130.0 | 130.3 | 335.1 | 456.2 | 727.9 | 1320.3 |
| 2196 | 814.6 | 61.9 | 181.1 | 150.0 | 147.1 | 86.2 | 184.0 | 213.4 | 240.9 |

## Supplementary Table 2E. Expression of *HOXA9*, probe set id 214651_s_at and 209905_at.

*n* = 211

|  |  | **214651_s_at** | | | | **209905_at** | | | |
| --- | --- | --- | --- | --- | --- | --- | --- | --- | --- |
| **Sample** | **PCR** | **MAS** | **dChip** | **RMA** | **GCRMA** | **MAS** | **dChip** | **RMA** | **GCRMA** |
| 322 | 0.0 | 2.0 | 9.8 | 18.7 | 5.5 | 1.4 | -30.0 | 55.0 | 12.0 |
| 1316 | 0.0 | 3.9 | 12.3 | 17.6 | 6.7 | 1.4 | -15.4 | 52.0 | 14.6 |
| 1448 | 0.0 | 270.7 | 461.2 | 613.7 | 814.5 | 160.9 | 246.3 | 436.8 | 485.5 |
| 2169 | 0.0 | 3.7 | 8.4 | 15.2 | 5.8 | 1.0 | -24.1 | 47.2 | 10.5 |
| 2170 | 0.0 | 3.4 | 15.0 | 19.3 | 7.4 | 2.7 | -27.4 | 54.9 | 17.7 |
| 2178 | 0.0 | 9.2 | 8.6 | 18.0 | 7.6 | 1.4 | -29.6 | 53.8 | 16.5 |
| 2179 | 0.0 | 4.3 | 8.8 | 19.2 | 6.5 | 0.9 | -29.0 | 41.9 | 8.1 |
| 2187 | 0.0 | 13.1 | 18.0 | 24.4 | 6.8 | 1.5 | -33.3 | 54.6 | 12.8 |
| 2189 | 0.0 | 4.7 | 11.6 | 18.8 | 5.3 | 0.6 | -31.0 | 37.3 | 7.1 |
| 2192 | 0.0 | 3.8 | 9.8 | 21.5 | 5.3 | 1.5 | -19.7 | 51.6 | 17.2 |
| 2197 | 0.0 | 2.0 | 9.2 | 16.7 | 5.1 | 1.0 | -22.0 | 46.9 | 7.5 |
| 2200 | 0.0 | 5.1 | 14.7 | 19.1 | 8.2 | 3.8 | -30.7 | 46.4 | 23.2 |
| 2202 | 0.0 | 0.5 | 8.5 | 16.3 | 8.1 | 2.7 | -29.0 | 61.6 | 16.2 |
| 2204 | 0.0 | 1.8 | 11.7 | 19.1 | 5.9 | 1.3 | -26.4 | 49.7 | 15.7 |
| 2208 | 0.0 | 13.3 | 14.6 | 22.7 | 7.1 | 1.1 | -29.2 | 52.8 | 8.6 |
| 2218 | 0.0 | 2.2 | 6.6 | 21.2 | 5.0 | 1.3 | -25.5 | 51.4 | 7.4 |
| 2230 | 0.0 | 7.6 | 12.8 | 21.3 | 5.3 | 1.5 | -28.1 | 53.4 | 7.6 |
| 2234 | 0.0 | 0.7 | 5.0 | 18.5 | 5.0 | 1.7 | -30.1 | 49.2 | 7.3 |
| 2235 | 0.0 | 2.9 | 8.3 | 15.9 | 6.1 | 0.7 | -22.5 | 45.3 | 7.0 |
| 2237 | 0.0 | 1.3 | 4.0 | 18.4 | 4.9 | 1.7 | -22.6 | 50.1 | 9.5 |
| 2240 | 0.0 | 5.6 | 12.8 | 20.5 | 4.8 | 1.3 | -35.2 | 41.7 | 7.7 |
| 2253 | 0.0 | 5.4 | 9.5 | 17.0 | 7.2 | 2.0 | -39.0 | 56.5 | 14.8 |
| 2266 | 0.0 | 3.3 | 3.8 | 19.3 | 8.5 | 3.4 | -26.0 | 62.1 | 44.0 |
| 2267 | 0.0 | 8.6 | 17.9 | 20.6 | 7.7 | 1.7 | -26.4 | 54.0 | 13.3 |
| 2288 | 0.0 | 761.4 | 873.3 | 1568.9 | 2519.8 | 516.1 | 558.1 | 1137.3 | 1532.1 |
| 2289 | 0.0 | 505.1 | 698.1 | 1071.7 | 1706.9 | 356.4 | 415.2 | 762.8 | 1009.6 |
| 2297 | 0.0 | 3.4 | 3.4 | 14.7 | 6.5 | 2.5 | -26.9 | 62.1 | 13.3 |
| 2307 | 0.0 | 24.4 | 5.9 | 25.0 | 9.8 | 3.9 | -49.5 | 75.8 | 20.7 |
| 2468 | 0.0 | 4.7 | 6.8 | 21.5 | 10.0 | 1.8 | -33.4 | 58.1 | 22.3 |
| 2509 | 0.0 | 15.1 | 27.7 | 24.9 | 13.2 | 2.6 | -16.7 | 90.6 | 30.0 |
| 2511 | 0.0 | 6.6 | 10.7 | 25.6 | 6.9 | 1.1 | -28.4 | 38.3 | 7.3 |
| 2516 | 0.0 | 2.5 | 7.6 | 19.3 | 8.0 | 1.2 | -33.0 | 53.6 | 9.4 |
| 2534 | 0.0 | 8.1 | 8.6 | 20.0 | 6.0 | 2.5 | -25.5 | 54.9 | 18.2 |
| 2545 | 0.0 | 5.8 | 13.8 | 19.7 | 6.6 | 2.1 | -20.6 | 49.4 | 13.7 |
| 2549 | 0.0 | 5.5 | 15.2 | 18.6 | 7.2 | 1.4 | -32.9 | 49.2 | 14.6 |
| 2695 | 0.0 | 5.6 | 16.3 | 18.8 | 7.7 | 1.7 | -16.2 | 68.6 | 17.4 |
| 2751 | 0.0 | 4.5 | 19.8 | 22.8 | 12.9 | 2.1 | -32.1 | 50.2 | 8.9 |
| 2752 | 0.0 | 1.4 | 4.3 | 18.5 | 5.0 | 0.9 | -22.6 | 48.4 | 12.8 |
| 2764 | 0.0 | 9.7 | 18.1 | 19.6 | 8.9 | 3.3 | -25.8 | 61.0 | 26.6 |
| 2273 | 0.0 | 5.1 | 14.2 | 19.5 | 7.4 | 1.4 | -29.1 | 57.1 | 11.6 |
| 2748 | 0.0 | 7.4 | 11.7 | 15.5 | 6.8 | 1.6 | -18.3 | 54.7 | 15.0 |
| 2753 | 0.0 | 1.7 | 11.3 | 19.6 | 5.2 | 1.1 | -19.4 | 59.0 | 11.9 |
| 2242 | 0.0 | 1.2 | 16.4 | 20.4 | 11.2 | 1.8 | -25.2 | 52.8 | 7.8 |
| 2680 | 0.1 | 8.4 | 17.7 | 21.4 | 7.2 | 1.2 | -32.9 | 47.1 | 7.9 |
| 2762 | 0.1 | 3.2 | 12.5 | 18.8 | 9.1 | 2.1 | -38.0 | 50.6 | 23.4 |
| 2497 | 0.1 | 1.7 | 0.7 | 22.7 | 7.4 | 3.4 | -28.3 | 56.2 | 10.6 |
| 2767 | 0.1 | 6.1 | 14.8 | 20.2 | 7.1 | 1.2 | -28.0 | 47.3 | 14.2 |
| 2233 | 0.1 | 343.1 | 524.0 | 709.7 | 1054.2 | 251.8 | 344.3 | 553.2 | 656.9 |
| 2666 | 0.1 | 14.6 | 16.3 | 21.5 | 6.4 | 1.2 | -21.4 | 38.3 | 6.5 |
| 2658 | 0.1 | 3.9 | 7.2 | 13.9 | 4.9 | 1.5 | -15.6 | 51.9 | 16.4 |
| 2219 | 0.2 | 6.2 | 9.5 | 13.8 | 5.1 | 1.6 | -15.2 | 43.7 | 10.8 |
| 2263 | 0.2 | 5.7 | -0.6 | 16.5 | 6.9 | 0.7 | -26.8 | 50.8 | 7.5 |
| 2262 | 0.2 | 12.7 | 20.6 | 19.3 | 7.3 | 1.7 | -31.7 | 47.8 | 19.7 |
| 2678 | 0.2 | 4.1 | 16.4 | 19.0 | 6.8 | 1.4 | -22.4 | 48.5 | 8.1 |
| 2227 | 0.2 | 386.9 | 548.2 | 673.3 | 1043.5 | 246.1 | 222.0 | 493.6 | 639.4 |
| 2671 | 0.2 | 3.6 | 11.1 | 16.8 | 7.4 | 1.1 | -20.3 | 52.0 | 8.2 |
| 2750 | 0.2 | 3.4 | 4.2 | 15.6 | 7.7 | 1.0 | -30.6 | 49.3 | 12.4 |
| 2259 | 0.3 | 4.7 | 10.5 | 17.9 | 6.0 | 1.7 | -23.2 | 58.6 | 15.2 |
| 2250 | 0.3 | 9.7 | 17.2 | 21.1 | 6.3 | 0.9 | -13.4 | 48.1 | 10.8 |
| 2510 | 0.3 | 4.3 | 12.7 | 19.6 | 7.4 | 1.9 | -20.9 | 52.2 | 15.0 |
| 2301 | 0.3 | 0.5 | 5.7 | 18.4 | 5.0 | 1.4 | -19.1 | 48.0 | 7.2 |
| 2211 | 0.4 | 6.5 | 12.3 | 18.5 | 7.3 | 1.2 | -25.2 | 47.9 | 8.8 |
| 2210 | 0.4 | 11.3 | 21.5 | 20.8 | 8.6 | 1.5 | -26.9 | 73.8 | 26.9 |
| 2466 | 0.5 | 1.3 | 16.2 | 21.0 | 9.8 | 1.8 | -27.2 | 58.6 | 20.1 |
| 2287 | 0.5 | 9.0 | 14.8 | 19.5 | 6.9 | 1.0 | -17.0 | 53.2 | 8.8 |
| 2304 | 0.7 | 12.7 | 21.0 | 22.9 | 9.2 | 1.9 | -21.1 | 62.3 | 26.3 |
| 2238 | 0.9 | 20.4 | 31.3 | 22.9 | 12.2 | 3.5 | -13.1 | 52.3 | 14.4 |
| 2243 | 1.6 | 2.5 | 8.2 | 19.0 | 6.7 | 2.3 | -19.3 | 63.0 | 17.1 |
| 2283 | 2.0 | 20.2 | 34.5 | 26.8 | 29.6 | 3.7 | -19.2 | 83.3 | 35.1 |
| 2172 | 2.4 | 45.1 | 109.8 | 90.9 | 133.4 | 25.8 | 16.6 | 95.5 | 97.5 |
| 2249 | 3.2 | 34.0 | 64.9 | 55.7 | 48.5 | 5.2 | -9.5 | 81.5 | 52.7 |
| 2265 | 3.7 | 8.8 | 19.0 | 19.4 | 12.6 | 1.3 | -30.9 | 56.7 | 12.9 |
| 2171 | 3.9 | 174.8 | 308.5 | 369.2 | 504.0 | 114.9 | 142.6 | 283.8 | 287.3 |
| 2542 | 5.2 | 51.9 | 117.9 | 107.1 | 112.7 | 23.0 | 32.7 | 108.1 | 77.2 |
| 2766 | 5.3 | 80.3 | 125.8 | 114.3 | 146.3 | 13.9 | 22.3 | 111.0 | 97.0 |
| 2254 | 6.8 | 89.1 | 185.1 | 196.7 | 233.6 | 29.5 | 50.5 | 140.3 | 111.2 |
| 2228 | 7.6 | 112.3 | 252.8 | 240.5 | 312.9 | 71.4 | 87.3 | 200.1 | 171.6 |
| 2274 | 7.9 | 55.9 | 106.2 | 80.1 | 123.0 | 26.3 | 22.4 | 136.1 | 81.6 |
| 2327 | 7.9 | 96.2 | 193.4 | 218.8 | 274.0 | 48.8 | 72.2 | 154.1 | 162.9 |
| 2656 | 7.9 | 151.2 | 281.3 | 313.3 | 410.8 | 113.2 | 135.4 | 248.0 | 286.1 |
| 2223 | 8.5 | 70.7 | 147.3 | 134.1 | 207.1 | 21.7 | 26.0 | 142.3 | 101.5 |
| 2293 | 8.6 | 38.3 | 62.2 | 43.6 | 55.0 | 3.8 | -11.7 | 83.3 | 30.6 |
| 2251 | 10.5 | 33.8 | 48.6 | 39.2 | 53.3 | 6.6 | -8.1 | 98.2 | 77.2 |
| 2190 | 11.0 | 175.1 | 247.9 | 267.5 | 465.9 | 73.6 | 90.4 | 233.7 | 249.9 |
| 2747 | 11.9 | 241.7 | 341.9 | 416.3 | 588.7 | 119.4 | 169.6 | 329.8 | 366.7 |
| 2279 | 12.3 | 37.0 | 74.3 | 64.6 | 74.7 | 5.6 | -11.0 | 91.9 | 59.5 |
| 2696 | 14.7 | 105.3 | 150.4 | 160.0 | 203.6 | 35.8 | 35.0 | 152.0 | 155.5 |
| 2655 | 15.2 | 325.9 | 474.7 | 654.1 | 1042.8 | 220.9 | 291.5 | 462.7 | 552.2 |
| 2185 | 15.6 | 380.2 | 592.6 | 907.7 | 1459.9 | 305.2 | 355.0 | 669.9 | 792.9 |
| 2186 | 19.1 | 200.0 | 345.1 | 398.0 | 556.5 | 124.5 | 149.4 | 326.4 | 359.0 |
| 2175 | 19.6 | 542.8 | 671.4 | 958.9 | 1567.5 | 370.5 | 405.6 | 807.7 | 1045.0 |
| 2193 | 19.7 | 729.9 | 681.2 | 1315.8 | 2251.5 | 414.2 | 462.3 | 836.0 | 1169.2 |
| 2191 | 20.0 | 213.9 | 303.3 | 458.4 | 635.8 | 112.3 | 146.7 | 315.8 | 296.5 |
| 2690 | 20.4 | 330.4 | 412.5 | 502.1 | 835.5 | 204.1 | 215.1 | 468.3 | 588.9 |
| 2668 | 21.2 | 238.1 | 284.9 | 444.9 | 696.0 | 182.0 | 202.9 | 391.2 | 449.9 |
| 2194 | 21.6 | 791.7 | 985.1 | 1611.8 | 2740.2 | 597.7 | 652.6 | 1208.5 | 1733.0 |
| 2514 | 23.7 | 404.9 | 445.8 | 581.8 | 929.6 | 275.5 | 248.2 | 477.4 | 669.1 |
| 2773 | 23.8 | 200.1 | 278.5 | 291.1 | 509.6 | 143.4 | 141.6 | 307.7 | 382.8 |
| 2256 | 23.9 | 211.6 | 340.3 | 419.2 | 615.7 | 167.0 | 179.9 | 317.5 | 361.8 |
| 2215 | 24.4 | 84.5 | 169.4 | 148.5 | 226.6 | 32.8 | 52.1 | 158.5 | 136.0 |
| 2198 | 25.5 | 470.5 | 680.4 | 907.1 | 1382.9 | 276.8 | 299.3 | 601.6 | 767.8 |
| 2195 | 25.6 | 707.2 | 852.9 | 1399.0 | 2463.7 | 605.4 | 564.5 | 1207.3 | 1681.0 |
| 2176 | 27.8 | 526.8 | 703.7 | 1003.6 | 1584.1 | 322.4 | 367.4 | 699.0 | 796.5 |
| 2525 | 27.8 | 488.5 | 557.0 | 742.6 | 1234.5 | 261.2 | 285.7 | 546.1 | 695.7 |
| 2644 | 28.1 | 803.5 | 1000.4 | 1548.8 | 3080.1 | 678.9 | 570.5 | 1243.4 | 1887.4 |
| 2749 | 28.4 | 530.0 | 662.3 | 986.3 | 1682.4 | 431.3 | 398.0 | 725.5 | 967.9 |
| 2222 | 28.9 | 221.7 | 316.6 | 399.9 | 628.0 | 117.1 | 127.0 | 298.9 | 347.3 |
| 2507 | 30.2 | 584.9 | 720.6 | 1162.4 | 1991.6 | 376.9 | 359.2 | 672.7 | 879.2 |
| 2299 | 30.3 | 233.1 | 301.3 | 372.5 | 567.2 | 139.9 | 135.3 | 317.6 | 365.8 |
| 1595 | 31.0 | 179.9 | 316.9 | 341.9 | 460.7 | 114.4 | 128.8 | 298.2 | 317.9 |
| 2490 | 31.1 | 611.5 | 794.0 | 1230.4 | 2064.5 | 398.0 | 366.2 | 701.6 | 911.3 |
| 2199 | 32.1 | 941.9 | 1097.3 | 1808.5 | 3424.8 | 815.6 | 779.3 | 1793.7 | 2528.3 |
| 2252 | 32.3 | 605.4 | 798.0 | 1150.3 | 2044.3 | 398.0 | 436.5 | 824.9 | 1052.8 |
| 2260 | 35.4 | 195.0 | 369.5 | 451.3 | 600.5 | 108.7 | 154.4 | 306.0 | 320.0 |
| 2694 | 37.1 | 688.1 | 925.6 | 1336.8 | 2656.7 | 595.6 | 594.0 | 1184.7 | 1922.4 |
| 2480 | 37.4 | 434.8 | 662.2 | 904.0 | 1396.3 | 254.9 | 322.7 | 570.9 | 697.5 |
| 2543 | 38.5 | 149.0 | 255.1 | 287.9 | 375.5 | 95.0 | 120.7 | 231.5 | 256.9 |
| 2261 | 40.6 | 487.8 | 645.5 | 1029.1 | 1582.0 | 327.7 | 374.2 | 648.3 | 796.4 |
| 1551 | 40.8 | 944.6 | 799.3 | 1606.7 | 2920.8 | 696.4 | 572.4 | 1276.0 | 1747.4 |
| 2224 | 40.8 | 607.6 | 750.0 | 1275.2 | 2013.7 | 396.6 | 418.8 | 765.9 | 1025.0 |
| 2467 | 41.8 | 351.2 | 512.8 | 671.3 | 1073.7 | 220.5 | 259.8 | 445.3 | 526.8 |
| 2182 | 44.5 | 642.8 | 912.1 | 1269.3 | 2263.0 | 503.3 | 570.4 | 1077.1 | 1404.3 |
| 2757 | 47.5 | 561.8 | 710.4 | 1007.9 | 2135.9 | 419.8 | 366.6 | 817.0 | 1286.4 |
| 1188 | 48.8 | 270.4 | 435.1 | 554.0 | 826.9 | 181.0 | 234.8 | 409.8 | 488.1 |
| 2535 | 49.7 | 1072.8 | 1143.2 | 1892.6 | 3279.5 | 673.2 | 687.6 | 1360.4 | 2082.3 |
| 2285 | 50.2 | 677.9 | 765.2 | 1281.9 | 2275.8 | 413.3 | 487.0 | 845.1 | 1084.2 |
| 2524 | 50.7 | 840.5 | 843.1 | 1578.5 | 2850.7 | 768.6 | 578.8 | 1262.7 | 1851.2 |
| 2270 | 52.7 | 516.3 | 759.5 | 1153.4 | 1915.4 | 338.5 | 433.1 | 835.9 | 1086.4 |
| 2183 | 52.9 | 719.4 | 927.4 | 1606.3 | 2977.4 | 583.2 | 625.8 | 1147.9 | 1422.8 |
| 2239 | 53.5 | 648.4 | 762.9 | 1249.8 | 1980.5 | 334.6 | 409.8 | 792.5 | 902.4 |
| 2664 | 55.1 | 292.8 | 348.7 | 433.0 | 659.9 | 135.0 | 142.0 | 356.4 | 455.5 |
| 2206 | 57.5 | 652.7 | 780.9 | 1137.6 | 2119.7 | 480.9 | 490.6 | 952.7 | 1326.2 |
| 2244 | 57.7 | 274.7 | 442.8 | 553.8 | 767.1 | 174.6 | 207.4 | 437.1 | 476.4 |
| 2181 | 58.5 | 820.0 | 1001.5 | 1680.3 | 2915.2 | 579.1 | 640.0 | 1373.5 | 1694.3 |
| 2241 | 60.6 | 649.1 | 786.3 | 1414.7 | 2395.9 | 489.2 | 520.0 | 1107.9 | 1419.9 |
| 2291 | 60.6 | 656.4 | 740.2 | 1104.6 | 1910.8 | 415.2 | 444.0 | 794.1 | 931.8 |
| 2177 | 61.4 | 825.2 | 873.2 | 1652.1 | 2971.6 | 534.5 | 576.7 | 1090.2 | 1434.4 |
| 2771 | 62.5 | 614.4 | 878.4 | 1110.3 | 1779.4 | 421.2 | 484.6 | 868.1 | 1225.6 |
| 2676 | 63.3 | 589.8 | 673.8 | 972.3 | 1650.9 | 386.3 | 394.2 | 768.7 | 982.9 |
| 2276 | 64.0 | 226.3 | 376.5 | 397.5 | 601.9 | 118.9 | 142.2 | 322.9 | 355.7 |
| 2212 | 65.3 | 235.3 | 370.8 | 425.9 | 616.8 | 140.3 | 167.4 | 323.3 | 368.7 |
| 2683 | 65.3 | 989.5 | 1160.2 | 1690.2 | 3516.0 | 773.6 | 753.7 | 1620.5 | 2436.9 |
| 2184 | 65.8 | 1264.3 | 1069.3 | 2289.1 | 4146.3 | 828.3 | 823.9 | 1507.0 | 2287.3 |
| 2669 | 65.8 | 539.9 | 790.8 | 1031.2 | 1703.7 | 359.0 | 441.9 | 784.0 | 996.5 |
| 2209 | 66.5 | 655.2 | 773.6 | 1212.6 | 2062.1 | 518.1 | 480.9 | 906.1 | 1205.9 |
| 1747 | 67.7 | 927.3 | 1146.8 | 2090.6 | 3869.2 | 763.5 | 838.2 | 1680.5 | 2477.5 |
| 2255 | 69.1 | 141.8 | 290.7 | 284.2 | 389.4 | 104.5 | 130.2 | 267.9 | 269.5 |
| 2257 | 73.0 | 748.4 | 815.2 | 1261.2 | 2388.7 | 399.9 | 466.4 | 900.5 | 1198.1 |
| 2174 | 74.8 | 777.0 | 886.1 | 1473.1 | 2647.9 | 581.1 | 498.8 | 1137.6 | 1692.0 |
| 2205 | 76.9 | 874.7 | 912.4 | 1563.9 | 2751.8 | 608.0 | 587.5 | 1143.1 | 1545.0 |
| 2196 | 78.2 | 975.0 | 1238.8 | 2127.5 | 4018.2 | 753.6 | 779.2 | 1607.4 | 2393.2 |
| 2290 | 80.4 | 218.5 | 352.6 | 424.1 | 636.8 | 142.1 | 158.6 | 310.3 | 326.3 |
| 2756 | 81.0 | 577.8 | 755.6 | 1030.8 | 1770.5 | 435.2 | 503.0 | 924.7 | 1203.5 |
| 2687 | 81.6 | 666.7 | 645.5 | 1122.6 | 1957.3 | 401.1 | 412.3 | 762.1 | 1147.0 |
| 2188 | 83.3 | 560.4 | 758.1 | 1223.8 | 2144.3 | 448.5 | 525.7 | 1011.7 | 1302.0 |
| 2247 | 83.3 | 638.7 | 756.7 | 1125.8 | 1868.5 | 452.6 | 475.0 | 844.2 | 1147.8 |
| 2679 | 83.3 | 847.7 | 973.2 | 1613.5 | 2900.1 | 761.1 | 697.9 | 1625.6 | 2577.7 |
| 2173 | 84.7 | 1021.4 | 1112.3 | 1935.9 | 3567.6 | 889.9 | 715.6 | 1735.3 | 2629.7 |
| 2275 | 85.0 | 505.4 | 636.8 | 1048.3 | 1699.9 | 444.7 | 439.9 | 785.1 | 1107.3 |
| 2236 | 89.9 | 852.8 | 959.3 | 1687.2 | 2931.8 | 647.8 | 649.4 | 1368.0 | 1915.5 |
| 2282 | 92.4 | 817.5 | 953.2 | 1676.5 | 2961.4 | 815.1 | 749.0 | 1589.3 | 2279.1 |
| 2665 | 92.7 | 921.6 | 1019.7 | 1816.0 | 3057.4 | 680.2 | 672.0 | 1279.9 | 1938.6 |
| 2272 | 93.7 | 490.6 | 710.7 | 978.3 | 1563.6 | 317.0 | 376.1 | 702.4 | 855.5 |
| 2670 | 93.7 | 553.2 | 731.4 | 1152.4 | 1763.6 | 393.4 | 505.1 | 814.8 | 1084.2 |
| 2688 | 94.4 | 963.0 | 1060.1 | 1591.2 | 3215.2 | 633.8 | 516.9 | 1142.1 | 1880.1 |
| 2672 | 95.0 | 739.0 | 1004.7 | 1580.4 | 2790.0 | 661.5 | 611.4 | 1221.8 | 1869.6 |
| 2248 | 95.3 | 809.7 | 1052.8 | 1516.0 | 3005.4 | 570.6 | 656.5 | 1488.4 | 1857.2 |
| 2692 | 96.7 | 1449.6 | 1369.8 | 2243.6 | 3940.8 | 797.3 | 664.2 | 1445.6 | 2274.2 |
| 2774 | 97.7 | 772.4 | 933.3 | 1469.8 | 2596.0 | 535.8 | 620.5 | 1115.4 | 1591.3 |
| 2498 | 101.1 | 571.2 | 679.4 | 937.1 | 1671.3 | 353.2 | 314.8 | 683.6 | 980.3 |
| 2207 | 102.2 | 972.8 | 1195.5 | 2063.2 | 3868.3 | 802.3 | 747.2 | 1569.7 | 2172.6 |
| 2765 | 102.9 | 1266.1 | 1188.9 | 2074.0 | 3352.7 | 798.1 | 883.7 | 1586.2 | 2268.2 |
| 2686 | 104.3 | 646.4 | 764.4 | 1223.7 | 2145.4 | 546.9 | 523.6 | 1006.1 | 1501.3 |
| 2225 | 106.2 | 910.2 | 995.2 | 1922.2 | 3359.3 | 693.3 | 643.3 | 1482.5 | 1924.4 |
| 2541 | 107.6 | 807.3 | 809.3 | 1481.9 | 2434.9 | 614.6 | 609.9 | 1244.8 | 1693.6 |
| 2772 | 109.5 | 866.1 | 912.4 | 1401.7 | 2309.2 | 589.0 | 603.9 | 1079.1 | 1570.8 |
| 2284 | 111.0 | 794.5 | 914.8 | 1665.7 | 3068.0 | 552.4 | 599.0 | 1179.5 | 1694.7 |
| 2305 | 111.0 | 600.2 | 683.6 | 1093.4 | 1877.8 | 371.4 | 323.6 | 703.7 | 995.1 |
| 2201 | 113.8 | 836.1 | 931.3 | 1578.3 | 2985.0 | 562.3 | 523.5 | 1106.4 | 1679.3 |
| 1766 | 114.6 | 1013.1 | 1247.2 | 1937.9 | 3526.8 | 738.6 | 760.3 | 1600.2 | 2278.6 |
| 2769 | 116.2 | 1085.3 | 1226.5 | 2034.0 | 3850.6 | 1082.3 | 907.6 | 1904.9 | 3386.4 |
| 2306 | 118.2 | 1004.8 | 965.2 | 1831.0 | 3064.5 | 569.8 | 609.8 | 1230.8 | 1601.7 |
| 2551 | 134.4 | 244.8 | 358.5 | 432.7 | 651.8 | 193.4 | 175.1 | 389.0 | 490.0 |
| 2229 | 135.3 | 762.1 | 835.4 | 1384.3 | 2468.7 | 548.1 | 525.8 | 1040.2 | 1371.0 |
| 2685 | 135.8 | 496.2 | 654.4 | 931.9 | 1521.6 | 387.8 | 385.0 | 704.6 | 1003.0 |
| 1174 | 137.7 | 677.7 | 686.6 | 1190.5 | 2037.0 | 476.3 | 455.5 | 990.1 | 1404.0 |
| 2278 | 141.5 | 414.4 | 607.8 | 809.1 | 1447.8 | 356.6 | 310.9 | 622.8 | 841.9 |
| 2231 | 142.0 | 620.9 | 789.9 | 1126.2 | 1741.2 | 438.8 | 480.5 | 889.3 | 1097.8 |
| 1432 | 149.6 | 941.8 | 946.6 | 1712.0 | 2934.7 | 949.5 | 828.4 | 1815.6 | 2530.2 |
| 2292 | 151.2 | 761.9 | 842.5 | 1517.5 | 2703.1 | 577.9 | 550.2 | 1057.6 | 1529.3 |
| 1197 | 154.9 | 197.9 | 319.8 | 335.8 | 513.3 | 136.7 | 145.0 | 338.3 | 350.5 |
| 2246 | 154.9 | 850.5 | 898.4 | 1705.0 | 2971.4 | 567.4 | 576.6 | 1284.8 | 1694.0 |
| 2296 | 164.9 | 317.0 | 467.4 | 642.3 | 967.7 | 174.0 | 204.7 | 419.0 | 521.1 |
| 2216 | 166.0 | 471.1 | 635.2 | 966.7 | 1447.0 | 305.9 | 351.4 | 710.1 | 796.7 |
| 2217 | 167.7 | 1289.7 | 1547.0 | 2550.2 | 4748.4 | 1127.1 | 1080.8 | 2227.7 | 3298.0 |
| 2300 | 168.3 | 1000.1 | 1374.4 | 2182.2 | 4143.6 | 925.5 | 871.7 | 1797.0 | 2800.2 |
| 2550 | 184.8 | 260.3 | 411.4 | 519.8 | 798.3 | 195.0 | 205.5 | 414.1 | 470.6 |
| 2203 | 210.8 | 1496.4 | 1376.2 | 2412.5 | 4392.0 | 1241.4 | 1178.8 | 2512.7 | 3844.4 |
| 2220 | 219.8 | 1451.4 | 1340.2 | 2582.5 | 4631.5 | 934.1 | 919.3 | 1804.7 | 2691.7 |
| 2271 | 226.8 | 1122.5 | 922.2 | 2156.9 | 3415.4 | 728.6 | 725.9 | 1447.1 | 2249.2 |
| 2286 | 227.5 | 799.1 | 733.4 | 1418.7 | 2395.6 | 432.7 | 447.1 | 803.2 | 1106.9 |
| 2326 | 248.1 | 1351.6 | 1324.3 | 2679.9 | 5409.2 | 1019.0 | 1018.3 | 2284.7 | 3663.7 |
| 2754 | 278.2 | 1013.1 | 1063.3 | 1812.1 | 3273.5 | 724.8 | 685.0 | 1486.4 | 2076.1 |
| 2689 | 282.1 | 1417.6 | 1494.1 | 2481.2 | 5011.8 | 1139.9 | 1066.0 | 2294.7 | 3738.4 |
| 2226 | 308.7 | 626.2 | 793.0 | 1254.9 | 2060.7 | 378.6 | 435.0 | 864.0 | 1045.2 |
| 2280 | 308.7 | 396.2 | 618.9 | 856.1 | 1307.1 | 320.2 | 341.4 | 558.0 | 713.2 |
| 2281 | 369.7 | 1863.9 | 1484.6 | 3339.2 | 5651.2 | 1397.5 | 1191.7 | 2693.4 | 4188.6 |
| 2268 | 372.2 | 1011.3 | 1222.1 | 1797.8 | 3751.6 | 602.3 | 576.1 | 1207.1 | 1856.7 |
| 2544 | 414.4 | 581.1 | 723.5 | 1010.7 | 1741.0 | 405.3 | 416.9 | 830.6 | 1053.9 |
| 2302 | 666.3 | 874.0 | 977.9 | 1576.2 | 3012.9 | 833.8 | 685.2 | 1390.5 | 2144.3 |
| 1299 | 719.1 | 1541.1 | 1186.6 | 2605.7 | 4670.4 | 1140.5 | 937.4 | 2156.1 | 3040.2 |

## Supplementary Table 2F. Expression of *PRDM1*, probe set id 217192_s_at.

*n* = 276

| **Sample** | **PCR** | **MAS** | **dChip** | **RMA** | **GCRMA** |
| --- | --- | --- | --- | --- | --- |
| 2193 | 0.4 | 45.8 | 97.0 | 122.8 | 104.5 |
| 2766 | 0.4 | 53.7 | 112.3 | 124.0 | 135.7 |
| 2257 | 0.6 | 9.2 | 54.6 | 98.9 | 54.7 |
| 2514 | 1.0 | 58.4 | 123.7 | 149.4 | 147.4 |
| 2680 | 1.0 | 15.6 | 69.5 | 79.8 | 72.3 |
| 3101 | 1.2 | 17.5 | 66.4 | 88.7 | 48.5 |
| 3277 | 1.3 | 16.1 | 72.9 | 96.7 | 72.9 |
| 2179 | 1.3 | 24.8 | 79.7 | 89.1 | 66.8 |
| 2670 | 1.3 | 15.9 | 72.0 | 83.7 | 48.5 |
| 2772 | 1.3 | 19.5 | 92.2 | 108.2 | 106.9 |
| 2205 | 1.5 | 8.1 | 67.9 | 95.4 | 73.2 |
| 2658 | 1.5 | 15.3 | 75.7 | 86.4 | 49.1 |
| 2267 | 1.5 | 21.9 | 79.5 | 98.2 | 96.7 |
| 2664 | 1.6 | 5.2 | 113.0 | 92.7 | 83.1 |
| 2690 | 1.7 | 11.1 | 107.0 | 114.3 | 70.5 |
| 2276 | 1.7 | 15.6 | 68.5 | 90.7 | 65.7 |
| 2288 | 1.8 | 8.7 | 74.7 | 96.6 | 70.8 |
| 2291 | 1.9 | 25.8 | 80.2 | 92.8 | 74.0 |
| 3096 | 2.0 | 62.7 | 99.8 | 118.6 | 112.9 |
| 2240 | 2.2 | 28.4 | 79.6 | 102.8 | 75.9 |
| 2170 | 2.2 | 28.3 | 99.1 | 104.4 | 94.0 |
| 2199 | 2.3 | 33.3 | 80.5 | 103.1 | 89.3 |
| 3316 | 2.3 | 15.2 | 65.3 | 86.9 | 57.1 |
| 3314 | 2.3 | 7.7 | 63.6 | 82.8 | 58.1 |
| 1595 | 2.3 | 9.6 | 80.6 | 95.1 | 64.0 |
| 3331 | 2.3 | 4.7 | 75.7 | 95.6 | 76.8 |
| 2668 | 2.3 | 20.1 | 81.8 | 99.7 | 70.5 |
| 3279 | 2.4 | 14.0 | 60.6 | 78.7 | 63.9 |
| 2173 | 2.5 | 17.0 | 89.2 | 115.4 | 86.9 |
| 2551 | 2.5 | 46.5 | 100.6 | 124.9 | 108.5 |
| 1448 | 2.6 | 5.5 | 98.3 | 115.5 | 99.0 |
| 2287 | 2.6 | 53.6 | 104.5 | 116.7 | 102.6 |
| 3099 | 2.7 | 27.6 | 88.4 | 105.0 | 102.1 |
| 2209 | 2.7 | 25.9 | 95.3 | 116.9 | 98.1 |
| 2194 | 2.7 | 28.1 | 95.5 | 103.4 | 81.9 |
| 2544 | 2.8 | 59.4 | 81.8 | 126.7 | 122.8 |
| 1747 | 2.9 | 18.1 | 65.5 | 88.4 | 49.9 |
| 3333 | 3.1 | 28.4 | 81.3 | 96.3 | 78.6 |
| 2545 | 3.1 | 15.2 | 79.4 | 97.3 | 80.9 |
| 2226 | 3.2 | 41.9 | 88.6 | 107.4 | 95.9 |
| 2236 | 3.3 | 9.2 | 80.1 | 98.5 | 76.0 |
| 2682 | 3.3 | 25.7 | 87.0 | 101.6 | 86.6 |
| 3312 | 3.3 | 5.3 | 71.1 | 90.5 | 70.1 |
| 2747 | 3.3 | 46.0 | 124.6 | 136.7 | 128.1 |
| 2175 | 3.3 | 43.4 | 93.9 | 111.3 | 103.3 |
| 2206 | 3.4 | 24.0 | 91.9 | 98.5 | 88.0 |
| 2220 | 3.4 | 39.3 | 97.6 | 100.5 | 90.1 |
| 2271 | 3.4 | 24.6 | 82.8 | 98.0 | 94.3 |
| 2208 | 3.4 | 17.0 | 79.4 | 96.7 | 67.3 |
| 2187 | 3.5 | 19.4 | 84.8 | 94.5 | 72.8 |
| 2694 | 3.5 | 58.5 | 114.9 | 127.0 | 146.8 |
| 2683 | 3.7 | 17.1 | 95.2 | 142.9 | 108.4 |
| 2771 | 3.9 | 58.8 | 99.6 | 121.5 | 140.7 |
| 2214 | 3.9 | 35.8 | 106.6 | 115.5 | 100.0 |
| 2692 | 4.0 | 46.6 | 120.4 | 143.5 | 141.4 |
| 2516 | 4.1 | 26.8 | 81.1 | 91.3 | 102.4 |
| 2656 | 4.1 | 34.8 | 76.3 | 93.9 | 75.8 |
| 2184 | 4.1 | 23.9 | 79.0 | 95.9 | 78.6 |
| 2549 | 4.3 | 47.3 | 94.7 | 123.5 | 108.4 |
| 2644 | 4.3 | 34.5 | 85.7 | 101.0 | 107.5 |
| 2198 | 4.4 | 119.9 | 118.2 | 134.0 | 137.6 |
| 2241 | 4.4 | 7.0 | 64.9 | 82.9 | 49.4 |
| 2750 | 4.5 | 47.8 | 80.9 | 125.7 | 113.3 |
| 2268 | 4.5 | 27.4 | 96.4 | 132.5 | 107.6 |
| 2190 | 4.6 | 23.2 | 105.9 | 122.7 | 105.3 |
| 2507 | 4.7 | 47.8 | 112.9 | 132.4 | 130.6 |
| 2511 | 4.7 | 37.0 | 65.6 | 85.2 | 68.4 |
| 3327 | 4.8 | 33.2 | 78.4 | 113.4 | 97.1 |
| 3098 | 4.8 | 44.1 | 86.5 | 107.0 | 89.8 |
| 2195 | 4.9 | 39.7 | 97.0 | 120.1 | 103.9 |
| 2207 | 4.9 | 19.5 | 84.4 | 110.2 | 75.7 |
| 3322 | 5.0 | 18.2 | 82.7 | 101.3 | 89.6 |
| 2672 | 5.0 | 22.1 | 103.6 | 101.6 | 77.4 |
| 3482 | 5.1 | 28.4 | 101.4 | 112.8 | 106.0 |
| 2543 | 5.1 | 25.8 | 81.1 | 100.9 | 69.6 |
| 2233 | 5.3 | 8.9 | 85.1 | 93.8 | 79.7 |
| 3324 | 5.3 | 34.7 | 83.7 | 101.0 | 86.5 |
| 2681 | 5.3 | 26.5 | 120.0 | 129.6 | 94.3 |
| 3115 | 5.3 | 67.6 | 104.7 | 145.9 | 113.8 |
| 2204 | 5.3 | 35.0 | 83.6 | 120.4 | 104.1 |
| 2248 | 5.5 | 43.8 | 63.7 | 134.6 | 105.5 |
| 2197 | 5.6 | 28.4 | 63.4 | 90.5 | 73.5 |
| 2230 | 5.6 | 52.2 | 97.1 | 112.1 | 100.9 |
| 2261 | 5.7 | 55.7 | 101.4 | 126.7 | 124.5 |
| 2655 | 5.7 | 109.1 | 149.9 | 187.1 | 205.7 |
| 1201 | 5.7 | 24.2 | 67.9 | 110.6 | 89.5 |
| 2695 | 5.8 | 21.7 | 81.9 | 102.2 | 69.8 |
| 2686 | 5.9 | 17.4 | 74.1 | 109.9 | 89.5 |
| 3313 | 6.0 | 21.5 | 112.2 | 146.2 | 156.6 |
| 2176 | 6.2 | 32.0 | 75.5 | 102.3 | 78.1 |
| 2212 | 6.3 | 65.3 | 103.0 | 143.8 | 133.7 |
| 3286 | 6.3 | 26.5 | 75.3 | 88.2 | 79.6 |
| 3326 | 6.4 | 43.4 | 102.1 | 124.2 | 119.9 |
| 2764 | 6.4 | 57.4 | 116.9 | 127.1 | 108.1 |
| 3483 | 6.4 | 23.8 | 77.6 | 102.6 | 81.1 |
| 2242 | 6.5 | 11.1 | 80.9 | 104.6 | 62.2 |
| 322 | 6.5 | 7.6 | 71.8 | 85.0 | 64.9 |
| 2286 | 6.7 | 21.2 | 45.5 | 126.1 | 106.3 |
| 1551 | 6.8 | 56.8 | 71.2 | 85.5 | 65.5 |
| 2666 | 6.8 | 31.2 | 82.4 | 107.3 | 85.9 |
| 3308 | 6.9 | 14.0 | 67.6 | 100.5 | 73.1 |
| 2182 | 6.9 | 22.0 | 86.6 | 100.2 | 84.0 |
| 2254 | 7.0 | 35.0 | 86.1 | 106.7 | 92.9 |
| 2218 | 7.1 | 11.5 | 73.4 | 91.2 | 82.7 |
| 2769 | 7.1 | 53.6 | 107.3 | 128.3 | 115.8 |
| 2679 | 7.1 | 18.8 | 88.2 | 114.0 | 103.0 |
| 2275 | 7.2 | 39.1 | 107.9 | 117.1 | 104.2 |
| 2704 | 7.4 | 51.5 | 122.1 | 128.9 | 130.8 |
| 2189 | 7.5 | 37.5 | 104.4 | 98.0 | 98.1 |
| 2671 | 7.6 | 15.3 | 66.5 | 93.0 | 75.9 |
| 3285 | 7.6 | 26.1 | 102.3 | 110.2 | 105.7 |
| 3309 | 7.7 | 38.6 | 79.3 | 101.5 | 73.2 |
| 2669 | 7.7 | 68.4 | 128.1 | 145.3 | 130.8 |
| 3317 | 8.0 | 38.4 | 95.5 | 99.6 | 89.5 |
| 2181 | 8.1 | 40.6 | 113.8 | 112.5 | 101.3 |
| 3289 | 8.2 | 65.5 | 121.9 | 141.1 | 138.2 |
| 2274 | 8.3 | 69.7 | 108.9 | 141.7 | 136.9 |
| 2307 | 8.4 | 13.4 | 81.1 | 163.6 | 102.7 |
| 1299 | 8.5 | 26.9 | 74.2 | 108.0 | 85.6 |
| 2171 | 8.5 | 40.5 | 94.6 | 108.5 | 93.4 |
| 2306 | 8.5 | 24.5 | 85.9 | 103.6 | 79.8 |
| 2229 | 8.5 | 56.7 | 121.5 | 137.7 | 118.3 |
| 2774 | 8.6 | 11.9 | 81.3 | 96.4 | 78.2 |
| 3278 | 8.7 | 7.5 | 59.6 | 76.8 | 92.8 |
| 2200 | 8.7 | 27.5 | 97.9 | 123.0 | 107.1 |
| 2201 | 8.8 | 28.9 | 122.0 | 128.9 | 104.6 |
| 2490 | 8.8 | 37.6 | 99.8 | 125.6 | 120.1 |
| 1482 | 8.8 | 33.2 | 56.5 | 80.6 | 50.0 |
| 2535 | 9.0 | 43.7 | 99.3 | 113.7 | 104.5 |
| 2169 | 9.0 | 40.1 | 95.8 | 103.4 | 95.4 |
| 2748 | 9.2 | 24.2 | 76.9 | 85.9 | 73.0 |
| 2292 | 9.2 | 16.0 | 95.4 | 97.6 | 62.0 |
| 2687 | 9.3 | 40.5 | 97.3 | 131.1 | 105.4 |
| 2498 | 9.3 | 71.6 | 115.4 | 161.0 | 156.3 |
| 2749 | 9.4 | 36.9 | 95.4 | 128.6 | 104.7 |
| 2256 | 9.5 | 40.1 | 85.3 | 127.1 | 104.2 |
| 2301 | 9.5 | 27.6 | 73.5 | 106.2 | 97.4 |
| 1316 | 9.8 | 23.7 | 70.3 | 96.9 | 83.7 |
| 2688 | 9.9 | 95.4 | 106.7 | 163.8 | 197.6 |
| 2767 | 9.9 | 35.8 | 90.6 | 110.0 | 83.4 |
| 1401 | 10.1 | 27.3 | 84.8 | 103.8 | 89.1 |
| 2235 | 10.2 | 36.0 | 100.0 | 116.1 | 103.5 |
| 2757 | 10.3 | 14.2 | 94.3 | 113.6 | 107.2 |
| 3311 | 10.3 | 33.5 | 81.6 | 115.4 | 101.2 |
| 2192 | 10.4 | 30.5 | 93.8 | 116.3 | 102.1 |
| 2273 | 10.4 | 34.5 | 110.9 | 106.0 | 101.2 |
| 2250 | 10.6 | 22.1 | 64.3 | 70.3 | 69.6 |
| 3319 | 10.6 | 41.0 | 123.3 | 132.1 | 104.5 |
| 3100 | 10.8 | 38.9 | 81.7 | 120.5 | 142.2 |
| 1432 | 11.0 | 48.1 | 97.0 | 124.5 | 106.4 |
| 3486 | 11.0 | 76.2 | 132.0 | 137.9 | 120.5 |
| 2753 | 11.0 | 40.3 | 98.6 | 122.1 | 116.8 |
| 2177 | 11.2 | 35.8 | 97.2 | 122.7 | 102.2 |
| 2647 | 11.2 | 65.8 | 103.1 | 133.2 | 121.9 |
| 2196 | 11.2 | 93.2 | 174.8 | 211.0 | 226.8 |
| 2174 | 11.3 | 83.3 | 125.0 | 156.4 | 168.3 |
| 2249 | 11.4 | 190.1 | 237.9 | 404.4 | 485.6 |
| 2216 | 11.5 | 54.6 | 98.6 | 143.1 | 128.3 |
| 3320 | 11.6 | 12.4 | 55.4 | 73.4 | 50.5 |
| 3292 | 11.7 | 47.4 | 115.4 | 121.4 | 107.9 |
| 2224 | 11.7 | 27.5 | 68.3 | 106.6 | 74.4 |
| 2172 | 11.8 | 36.7 | 85.1 | 108.6 | 93.3 |
| 2480 | 11.8 | 21.3 | 77.7 | 83.7 | 70.2 |
| 2285 | 11.9 | 17.9 | 72.4 | 100.6 | 67.4 |
| 2219 | 12.0 | 23.9 | 61.0 | 72.3 | 57.7 |
| 2689 | 12.4 | 52.6 | 98.3 | 132.6 | 119.2 |
| 2186 | 12.5 | 19.5 | 82.0 | 107.7 | 91.0 |
| 2550 | 12.5 | 32.0 | 88.7 | 109.4 | 95.1 |
| 3293 | 12.6 | 42.1 | 104.7 | 137.5 | 105.2 |
| 2183 | 12.8 | 73.7 | 151.9 | 175.1 | 167.2 |
| 2509 | 12.8 | 12.9 | 93.2 | 125.2 | 108.1 |
| 3323 | 12.9 | 26.0 | 77.0 | 116.1 | 84.1 |
| 2245 | 12.9 | 17.3 | 56.8 | 95.1 | 72.9 |
| 2289 | 13.2 | 90.7 | 170.4 | 239.8 | 260.6 |
| 2234 | 13.2 | 44.3 | 105.9 | 152.4 | 128.4 |
| 2762 | 13.2 | 123.1 | 213.1 | 263.3 | 367.2 |
| 2661 | 13.3 | 39.7 | 89.5 | 102.9 | 82.7 |
| 3490 | 13.4 | 43.7 | 86.5 | 132.5 | 126.4 |
| 1188 | 13.7 | 6.6 | 83.6 | 90.9 | 84.0 |
| 2237 | 13.8 | 28.0 | 66.6 | 97.4 | 73.0 |
| 2536 | 13.8 | 62.4 | 113.7 | 151.4 | 135.1 |
| 2246 | 14.0 | 34.7 | 84.3 | 101.0 | 79.3 |
| 2281 | 14.1 | 28.1 | 101.8 | 115.7 | 102.3 |
| 3102 | 14.1 | 83.8 | 136.1 | 152.1 | 163.9 |
| 2524 | 14.1 | 26.6 | 114.7 | 137.0 | 107.3 |
| 2534 | 14.5 | 111.8 | 133.0 | 175.3 | 173.8 |
| 3489 | 14.7 | 29.7 | 90.3 | 112.6 | 89.4 |
| 2300 | 14.7 | 21.7 | 105.8 | 113.6 | 105.6 |
| 3295 | 14.8 | 45.0 | 76.7 | 116.2 | 84.0 |
| 2253 | 15.2 | 77.9 | 144.4 | 162.7 | 155.3 |
| 2227 | 15.3 | 39.0 | 167.9 | 162.1 | 200.5 |
| 3332 | 15.4 | 19.1 | 76.5 | 96.8 | 72.5 |
| 2678 | 15.4 | 174.1 | 279.1 | 299.8 | 373.0 |
| 3321 | 15.6 | 91.9 | 134.7 | 156.1 | 195.7 |
| 2217 | 16.0 | 56.8 | 113.0 | 126.1 | 109.9 |
| 2326 | 16.1 | 79.1 | 116.6 | 135.5 | 127.0 |
| 2696 | 16.8 | 47.0 | 127.5 | 147.1 | 136.2 |
| 2188 | 17.0 | 22.4 | 69.5 | 78.6 | 61.7 |
| 2265 | 17.3 | 33.9 | 98.9 | 111.0 | 104.7 |
| 2541 | 18.3 | 48.3 | 112.9 | 151.3 | 124.2 |
| 2244 | 18.4 | 121.5 | 185.1 | 203.8 | 278.3 |
| 1174 | 19.3 | 84.3 | 180.9 | 228.1 | 273.7 |
| 2231 | 19.3 | 53.8 | 115.1 | 139.3 | 126.8 |
| 2468 | 20.3 | 31.6 | 108.3 | 124.6 | 124.7 |
| 2210 | 21.0 | 91.0 | 152.7 | 200.3 | 216.9 |
| 1063 | 21.5 | 10.3 | 72.4 | 116.7 | 78.9 |
| 3485 | 21.9 | 41.3 | 141.8 | 177.7 | 190.1 |
| 2752 | 22.4 | 18.1 | 63.3 | 83.2 | 67.2 |
| 3329 | 22.9 | 46.9 | 113.1 | 126.8 | 125.3 |
| 2773 | 23.1 | 35.8 | 91.4 | 125.7 | 105.2 |
| 3481 | 23.3 | 27.3 | 114.2 | 170.8 | 133.8 |
| 3492 | 23.5 | 45.7 | 86.8 | 117.4 | 101.6 |
| 2243 | 23.5 | 31.7 | 109.1 | 115.6 | 100.4 |
| 2178 | 23.6 | 30.2 | 84.7 | 98.7 | 75.5 |
| 2185 | 23.6 | 59.4 | 126.7 | 175.4 | 154.9 |
| 2299 | 23.8 | 28.4 | 95.4 | 114.1 | 89.5 |
| 1766 | 24.0 | 49.2 | 108.2 | 125.5 | 106.9 |
| 2765 | 24.4 | 36.4 | 62.7 | 89.0 | 96.7 |
| 2667 | 24.6 | 51.0 | 114.9 | 139.1 | 161.9 |
| 2278 | 24.7 | 112.7 | 177.9 | 215.2 | 248.2 |
| 3334 | 24.7 | 59.0 | 94.0 | 118.4 | 102.7 |
| 2546 | 24.8 | 30.4 | 100.2 | 110.9 | 113.5 |
| 3315 | 25.1 | 124.1 | 207.9 | 272.0 | 370.7 |
| 2238 | 25.1 | 26.4 | 77.4 | 101.2 | 87.9 |
| 3097 | 25.1 | 56.1 | 103.3 | 116.6 | 110.0 |
| 2327 | 25.4 | 72.4 | 152.5 | 191.6 | 220.1 |
| 3491 | 25.8 | 22.4 | 80.0 | 98.9 | 76.3 |
| 3310 | 26.7 | 16.3 | 128.8 | 263.7 | 120.4 |
| 2282 | 27.1 | 165.3 | 222.7 | 267.6 | 298.9 |
| 3488 | 27.3 | 60.6 | 92.1 | 119.1 | 104.8 |
| 2239 | 28.0 | 24.5 | 79.3 | 93.6 | 82.4 |
| 2539 | 29.0 | 39.0 | 86.9 | 119.6 | 99.1 |
| 2203 | 29.9 | 52.2 | 129.5 | 135.0 | 145.2 |
| 2262 | 30.0 | 34.3 | 81.1 | 103.4 | 80.8 |
| 2290 | 30.6 | 48.6 | 109.3 | 137.9 | 127.1 |
| 2191 | 31.8 | 72.6 | 153.5 | 147.9 | 171.9 |
| 2260 | 32.0 | 279.5 | 388.8 | 459.6 | 966.4 |
| 2270 | 32.3 | 49.9 | 90.3 | 121.5 | 88.6 |
| 3318 | 33.0 | 45.7 | 114.5 | 134.5 | 130.8 |
| 2305 | 33.1 | 63.4 | 120.1 | 162.2 | 112.6 |
| 2263 | 33.3 | 22.7 | 68.9 | 98.3 | 75.0 |
| 2252 | 33.4 | 12.8 | 92.1 | 117.4 | 87.2 |
| 3304 | 33.5 | 96.2 | 164.9 | 203.0 | 238.5 |
| 2284 | 34.9 | 27.1 | 81.0 | 108.3 | 85.6 |
| 2685 | 35.8 | 103.4 | 130.6 | 184.5 | 197.9 |
| 2510 | 36.4 | 75.9 | 140.6 | 189.4 | 220.6 |
| 2467 | 39.6 | 88.5 | 176.6 | 197.5 | 293.5 |
| 3493 | 43.7 | 69.9 | 86.8 | 148.7 | 182.6 |
| 2247 | 44.0 | 34.9 | 91.6 | 107.0 | 99.0 |
| 1197 | 47.6 | 86.8 | 148.2 | 174.3 | 176.9 |
| 3325 | 56.2 | 55.6 | 98.5 | 121.4 | 106.0 |
| 2228 | 57.1 | 48.3 | 149.4 | 154.2 | 162.3 |
| 2466 | 59.4 | 23.4 | 93.1 | 117.4 | 87.3 |
| 2542 | 69.2 | 72.7 | 146.9 | 150.9 | 146.9 |
| 2296 | 69.3 | 125.7 | 261.2 | 323.2 | 478.4 |
| 2497 | 73.1 | 68.0 | 166.5 | 188.7 | 224.4 |
| 2525 | 78.6 | 60.3 | 161.3 | 154.5 | 166.0 |
| 2754 | 79.4 | 35.6 | 105.5 | 125.1 | 120.5 |
| 3330 | 80.8 | 95.0 | 171.2 | 180.6 | 211.5 |
| 3328 | 85.1 | 58.9 | 124.8 | 156.3 | 155.6 |
| 3301 | 94.1 | 124.1 | 155.6 | 169.9 | 184.0 |
| 2225 | 95.8 | 84.5 | 147.8 | 177.1 | 166.9 |
| 2222 | 106.4 | 76.0 | 121.4 | 141.3 | 145.7 |
| 2304 | 123.1 | 95.9 | 158.6 | 238.8 | 259.2 |
| 2302 | 130.8 | 21.5 | 98.1 | 140.7 | 102.2 |
| 2676 | 131.0 | 112.9 | 186.5 | 217.8 | 298.3 |
| 2202 | 135.9 | 330.6 | 436.3 | 585.7 | 886.4 |
| 3484 | 137.8 | 95.7 | 154.9 | 158.5 | 182.2 |
| 2215 | 153.9 | 299.2 | 392.5 | 538.3 | 1553.2 |
| 2259 | 164.2 | 150.8 | 264.5 | 282.9 | 390.5 |
| 2751 | 195.6 | 49.3 | 117.2 | 152.5 | 163.7 |
| 3487 | 239.4 | 164.2 | 214.0 | 260.8 | 265.0 |
| 2756 | 250.3 | 161.0 | 230.1 | 303.7 | 343.6 |
| 2272 | 261.9 | 123.6 | 224.5 | 235.9 | 345.0 |
| 2251 | 372.9 | 326.2 | 296.6 | 392.8 | 720.2 |
| 2665 | 602.5 | 33.4 | 72.7 | 88.9 | 91.6 |

## Supplementary Table 2G. Expression of *PRDM2*, probe set id’s 205277_at, 203057_s_at, 203056_s_at and 216433_s_at.

*n* = 270

|  | | **205277 _at** | | | | | **203057_s_at** | | | | |
| --- | --- | --- | --- | --- | --- | --- | --- | --- | --- | --- | --- |
| **Sample** | **PCR** | | **MAS** | **dChip** | **RMA** | **GCRMA** | | **MAS** | **dChip** | **RMA** | **GCRMA** |
| 3101 | 0.0 | | 54.2 | 116.2 | 100.3 | 62.1 | | 141.5 | 253.3 | 302.3 | 170.2 |
| 2200 | 0.0 | | 89.2 | 124.1 | 121.5 | 100.2 | | 249.0 | 331.3 | 344.1 | 329.9 |
| 2207 | 0.0 | | 79.3 | 108.1 | 118.8 | 88.5 | | 124.9 | 177.8 | 179.4 | 123.2 |
| 3482 | 0.0 | | 83.2 | 132.9 | 118.0 | 114.1 | | 259.9 | 386.9 | 450.0 | 383.1 |
| 2235 | 0.0 | | 83.4 | 156.9 | 149.9 | 125.3 | | 174.1 | 215.3 | 178.3 | 164.7 |
| 2225 | 0.0 | | 75.6 | 142.1 | 133.5 | 130.8 | | 278.3 | 381.2 | 297.4 | 270.7 |
| 2514 | 0.0 | | 81.0 | 120.7 | 107.2 | 64.5 | | 86.4 | 151.7 | 227.3 | 64.8 |
| 2766 | 0.1 | | 81.3 | 142.8 | 117.6 | 90.0 | | 106.3 | 168.3 | 175.8 | 122.0 |
| 3324 | 0.1 | | 87.3 | 155.5 | 137.2 | 140.3 | | 193.3 | 289.6 | 191.7 | 217.9 |
| 2267 | 0.1 | | 95.2 | 142.4 | 116.5 | 85.8 | | 159.7 | 248.0 | 284.8 | 187.2 |
| 3325 | 0.1 | | 113.1 | 183.4 | 161.5 | 161.9 | | 150.8 | 248.4 | 232.5 | 200.7 |
| 2704 | 0.2 | | 85.2 | 143.5 | 132.0 | 93.2 | | 232.8 | 254.6 | 357.1 | 245.5 |
| 1482 | 0.2 | | 57.4 | 164.7 | 146.5 | 155.4 | | 59.8 | 221.8 | 253.5 | 182.7 |
| 3316 | 0.3 | | 48.9 | 99.8 | 81.0 | 49.3 | | 83.1 | 169.4 | 169.4 | 144.2 |
| 2291 | 0.3 | | 59.9 | 119.8 | 109.5 | 74.0 | | 85.2 | 174.6 | 223.5 | 131.9 |
| 2216 | 0.4 | | 82.0 | 119.0 | 111.1 | 91.3 | | 135.9 | 239.4 | 206.4 | 156.5 |
| 3314 | 0.4 | | 97.9 | 163.9 | 159.0 | 145.3 | | 153.0 | 288.7 | 284.4 | 209.3 |
| 2270 | 0.4 | | 111.3 | 145.7 | 144.9 | 127.2 | | 370.9 | 482.4 | 518.0 | 528.5 |
| 2656 | 0.4 | | 51.7 | 105.2 | 91.8 | 63.5 | | 158.9 | 267.1 | 199.1 | 181.0 |
| 2285 | 0.4 | | 92.7 | 170.8 | 156.0 | 155.4 | | 101.0 | 175.4 | 256.6 | 122.9 |
| 3327 | 0.5 | | 112.1 | 156.6 | 147.3 | 171.0 | | 191.1 | 236.1 | 206.4 | 187.3 |
| 3485 | 0.5 | | 99.8 | 127.2 | 119.1 | 96.4 | | 119.6 | 115.3 | 245.7 | 96.4 |
| 1063 | 0.5 | | 107.1 | 158.2 | 160.5 | 127.2 | | 135.4 | 187.7 | 214.7 | 141.8 |
| 1595 | 0.5 | | 93.9 | 134.7 | 130.7 | 110.7 | | 146.8 | 233.1 | 220.4 | 159.9 |
| 2171 | 0.5 | | 85.1 | 154.5 | 137.5 | 140.6 | | 125.4 | 206.8 | 191.6 | 155.3 |
| 2662 | 0.5 | | 134.1 | 171.5 | 177.0 | 169.7 | | 130.1 | 183.1 | 335.7 | 154.4 |
| 2655 | 0.5 | | 57.1 | 112.8 | 103.4 | 67.1 | | 151.3 | 301.1 | 344.9 | 197.4 |
| 3318 | 0.5 | | 62.5 | 116.0 | 89.2 | 52.0 | | 151.7 | 217.1 | 248.6 | 184.4 |
| 3326 | 0.5 | | 55.9 | 97.7 | 81.0 | 52.9 | | 134.5 | 202.8 | 172.4 | 128.4 |
| 3279 | 0.5 | | 63.2 | 120.5 | 113.6 | 92.2 | | 134.0 | 242.3 | 164.3 | 165.6 |
| 3317 | 0.5 | | 88.8 | 140.7 | 138.9 | 104.2 | | 108.0 | 171.3 | 203.8 | 105.7 |
| 2233 | 0.5 | | 40.0 | 93.1 | 72.2 | 45.0 | | 109.9 | 196.3 | 137.1 | 131.8 |
| 2694 | 0.5 | | 102.1 | 124.2 | 122.2 | 90.9 | | 251.4 | 327.1 | 300.8 | 359.7 |
| 2749 | 0.5 | | 79.6 | 129.0 | 118.3 | 95.2 | | 150.6 | 215.7 | 251.9 | 190.4 |
| 2668 | 0.5 | | 70.7 | 100.9 | 97.8 | 59.6 | | 114.6 | 177.7 | 155.0 | 116.0 |
| 2256 | 0.6 | | 63.1 | 103.2 | 96.4 | 62.3 | | 211.2 | 313.5 | 281.7 | 284.8 |
| 2490 | 0.6 | | 45.2 | 95.6 | 94.5 | 56.0 | | 259.9 | 328.5 | 341.2 | 335.2 |
| 2220 | 0.6 | | 48.7 | 99.9 | 97.0 | 72.7 | | 164.3 | 285.6 | 278.6 | 215.5 |
| 3331 | 0.6 | | 101.1 | 161.7 | 146.2 | 128.7 | | 359.0 | 410.7 | 405.2 | 354.1 |
| 3322 | 0.6 | | 77.7 | 119.9 | 113.3 | 79.2 | | 91.0 | 177.1 | 182.3 | 96.4 |
| 2290 | 0.6 | | 68.5 | 106.9 | 98.9 | 66.4 | | 182.5 | 236.1 | 223.2 | 187.1 |
| 3315 | 0.6 | | 77.3 | 141.6 | 112.0 | 45.1 | | 151.2 | 158.4 | 231.3 | 86.5 |
| 2254 | 0.6 | | 47.3 | 101.3 | 94.0 | 63.4 | | 146.2 | 201.7 | 253.8 | 141.0 |
| 2224 | 0.6 | | 52.1 | 89.1 | 83.5 | 48.8 | | 297.1 | 384.9 | 360.7 | 359.0 |
| 2680 | 0.6 | | 77.5 | 121.4 | 108.6 | 74.1 | | 171.1 | 231.2 | 221.6 | 171.3 |
| 3285 | 0.6 | | 89.3 | 107.9 | 107.6 | 83.5 | | 125.6 | 191.4 | 161.2 | 119.3 |
| 2678 | 0.7 | | 64.9 | 125.6 | 104.6 | 67.4 | | 177.0 | 286.2 | 277.7 | 248.7 |
| 2672 | 0.7 | | 57.4 | 89.5 | 84.3 | 54.0 | | 127.6 | 196.2 | 220.9 | 160.0 |
| 2287 | 0.7 | | 73.1 | 142.1 | 119.7 | 105.9 | | 103.0 | 159.2 | 267.4 | 120.9 |
| 322 | 0.7 | | 83.7 | 134.9 | 132.2 | 118.0 | | 144.3 | 214.3 | 254.1 | 176.0 |
| 3115 | 0.7 | | 123.0 | 170.1 | 163.6 | 140.8 | | 185.8 | 207.5 | 296.4 | 184.3 |
| 2647 | 0.7 | | 64.0 | 121.2 | 113.8 | 80.1 | | 191.1 | 290.3 | 279.8 | 226.1 |
| 3309 | 0.7 | | 86.2 | 117.6 | 112.3 | 89.4 | | 155.7 | 229.9 | 251.7 | 145.1 |
| 3102 | 0.7 | | 53.6 | 103.7 | 85.0 | 56.9 | | 116.5 | 170.9 | 209.4 | 116.9 |
| 3334 | 0.7 | | 68.3 | 119.1 | 117.4 | 98.3 | | 158.3 | 262.4 | 352.4 | 196.8 |
| 3293 | 0.7 | | 78.8 | 147.6 | 136.2 | 127.5 | | 178.9 | 277.6 | 226.0 | 217.1 |
| 2240 | 0.7 | | 86.7 | 136.2 | 133.9 | 107.6 | | 127.0 | 157.8 | 195.0 | 103.4 |
| 3308 | 0.7 | | 71.6 | 120.1 | 108.5 | 84.3 | | 166.8 | 248.1 | 237.2 | 198.7 |
| 2539 | 0.7 | | 61.0 | 98.2 | 85.3 | 58.0 | | 158.2 | 241.2 | 226.0 | 171.7 |
| 2326 | 0.7 | | 75.7 | 137.7 | 107.0 | 88.5 | | 133.2 | 216.4 | 156.0 | 152.7 |
| 3329 | 0.7 | | 93.1 | 138.5 | 132.1 | 107.6 | | 120.5 | 182.8 | 172.7 | 134.5 |
| 3096 | 0.7 | | 49.8 | 105.3 | 83.0 | 58.5 | | 285.8 | 386.4 | 424.6 | 346.9 |
| 3277 | 0.8 | | 85.2 | 128.3 | 97.4 | 88.5 | | 143.5 | 187.8 | 235.7 | 138.0 |
| 2307 | 0.8 | | 109.6 | 108.0 | 100.7 | 71.8 | | 189.2 | 179.3 | 313.2 | 148.8 |
| 2243 | 0.8 | | 77.9 | 121.1 | 109.7 | 84.9 | | 170.3 | 238.6 | 242.2 | 188.0 |
| 2217 | 0.8 | | 51.6 | 110.2 | 97.2 | 75.4 | | 116.3 | 188.9 | 195.5 | 167.7 |
| 2222 | 0.8 | | 91.0 | 125.2 | 128.0 | 90.6 | | 126.7 | 223.0 | 210.7 | 153.1 |
| 2212 | 0.8 | | 52.1 | 82.5 | 74.3 | 58.0 | | 205.9 | 282.3 | 255.9 | 235.8 |
| 3486 | 0.8 | | 77.6 | 131.7 | 130.0 | 72.2 | | 67.7 | 114.6 | 192.3 | 77.0 |
| 2750 | 0.8 | | 55.6 | 108.1 | 89.6 | 60.7 | | 130.4 | 220.8 | 243.1 | 194.6 |
| 1201 | 0.8 | | 64.3 | 113.5 | 99.4 | 63.9 | | 193.4 | 307.6 | 242.1 | 237.8 |
| 2658 | 0.8 | | 58.4 | 94.3 | 92.9 | 56.8 | | 246.0 | 340.1 | 307.3 | 273.1 |
| 2246 | 0.8 | | 61.0 | 119.6 | 111.3 | 69.7 | | 208.4 | 314.8 | 355.4 | 271.7 |
| 2549 | 0.8 | | 63.6 | 101.6 | 100.1 | 69.8 | | 173.0 | 234.5 | 267.4 | 178.9 |
| 3321 | 0.8 | | 89.4 | 136.6 | 137.4 | 112.7 | | 142.9 | 251.6 | 222.5 | 166.2 |
| 2683 | 0.8 | | 121.5 | 134.6 | 129.8 | 94.5 | | 161.6 | 201.2 | 252.0 | 175.3 |
| 3330 | 0.8 | | 100.8 | 158.2 | 134.2 | 125.1 | | 146.5 | 216.3 | 141.3 | 175.4 |
| 2301 | 0.8 | | 66.5 | 97.2 | 91.3 | 60.5 | | 119.9 | 207.3 | 236.9 | 196.3 |
| 2757 | 0.8 | | 116.5 | 131.3 | 118.2 | 91.1 | | 144.2 | 191.8 | 231.5 | 152.9 |
| 3491 | 0.8 | | 73.6 | 112.7 | 107.9 | 77.2 | | 147.3 | 215.8 | 240.1 | 159.1 |
| 2176 | 0.8 | | 79.1 | 135.1 | 125.9 | 104.4 | | 134.1 | 217.7 | 254.1 | 149.4 |
| 2543 | 0.9 | | 43.5 | 83.7 | 70.9 | 42.6 | | 147.8 | 228.8 | 250.3 | 179.0 |
| 2507 | 0.9 | | 63.6 | 93.1 | 83.1 | 52.8 | | 214.0 | 277.6 | 312.3 | 226.2 |
| 2197 | 0.9 | | 82.4 | 129.3 | 113.7 | 89.7 | | 144.6 | 218.8 | 235.6 | 166.6 |
| 2686 | 0.9 | | 130.1 | 146.5 | 137.5 | 111.4 | | 100.6 | 147.3 | 196.0 | 135.1 |
| 2748 | 0.9 | | 104.2 | 176.2 | 137.8 | 123.1 | | 162.9 | 261.6 | 249.7 | 232.1 |
| 3289 | 0.9 | | 122.8 | 183.9 | 173.6 | 154.9 | | 523.6 | 578.2 | 770.6 | 712.7 |
| 3286 | 0.9 | | 68.5 | 128.3 | 112.8 | 79.4 | | 166.7 | 262.5 | 203.6 | 171.8 |
| 2187 | 0.9 | | 118.0 | 177.1 | 183.6 | 171.4 | | 161.8 | 212.8 | 211.5 | 154.3 |
| 2753 | 0.9 | | 73.2 | 126.3 | 110.6 | 78.1 | | 170.2 | 307.9 | 293.1 | 278.3 |
| 2541 | 1.0 | | 42.0 | 77.1 | 72.5 | 43.5 | | 124.5 | 215.8 | 279.7 | 182.8 |
| 2242 | 1.0 | | 78.6 | 125.2 | 114.1 | 94.1 | | 145.8 | 205.3 | 191.5 | 159.5 |
| 3481 | 1.0 | | 86.5 | 126.9 | 112.9 | 89.7 | | 124.7 | 191.6 | 307.1 | 108.2 |
| 1448 | 1.0 | | 102.5 | 121.3 | 117.7 | 69.8 | | 138.2 | 143.4 | 180.5 | 101.0 |
| 1197 | 1.0 | | 92.4 | 135.1 | 138.3 | 111.5 | | 145.2 | 191.0 | 184.1 | 136.1 |
| 2692 | 1.0 | | 156.5 | 205.3 | 176.4 | 176.7 | | 322.3 | 383.5 | 458.7 | 433.3 |
| 2300 | 1.0 | | 78.4 | 101.3 | 111.9 | 59.4 | | 164.9 | 194.3 | 262.9 | 137.6 |
| 2281 | 1.0 | | 58.7 | 101.1 | 94.1 | 56.3 | | 227.7 | 307.1 | 273.0 | 275.6 |
| 2199 | 1.0 | | 70.6 | 105.6 | 92.6 | 52.0 | | 209.6 | 301.9 | 228.3 | 266.8 |
| 2249 | 1.0 | | 53.0 | 86.9 | 79.4 | 44.9 | | 139.3 | 264.5 | 299.6 | 167.0 |
| 2305 | 1.0 | | 78.6 | 113.7 | 112.2 | 62.4 | | 83.6 | 103.5 | 185.8 | 62.5 |
| 2218 | 1.0 | | 53.2 | 96.5 | 86.5 | 60.3 | | 233.4 | 300.0 | 280.2 | 252.5 |
| 3311 | 1.0 | | 64.6 | 104.7 | 91.4 | 62.0 | | 155.7 | 246.8 | 271.8 | 195.8 |
| 3323 | 1.0 | | 92.7 | 145.8 | 139.8 | 85.9 | | 119.0 | 207.2 | 151.9 | 134.2 |
| 2189 | 1.0 | | 74.7 | 140.7 | 127.3 | 120.4 | | 146.1 | 267.6 | 235.2 | 184.9 |
| 3483 | 1.1 | | 66.9 | 97.1 | 105.6 | 78.9 | | 134.8 | 199.6 | 235.4 | 147.0 |
| 2644 | 1.1 | | 124.6 | 162.9 | 162.6 | 184.6 | | 546.0 | 747.6 | 513.5 | 876.6 |
| 2198 | 1.1 | | 90.6 | 155.3 | 132.2 | 106.5 | | 204.9 | 265.9 | 257.1 | 253.8 |
| 2747 | 1.1 | | 59.3 | 96.3 | 92.9 | 65.1 | | 237.5 | 337.8 | 323.6 | 265.8 |
| 2186 | 1.1 | | 85.8 | 134.3 | 145.2 | 110.7 | | 252.3 | 367.4 | 250.8 | 360.5 |
| 2666 | 1.1 | | 85.3 | 128.0 | 122.2 | 96.6 | | 164.4 | 237.8 | 205.4 | 165.1 |
| 1174 | 1.1 | | 71.7 | 116.7 | 123.7 | 66.4 | | 163.1 | 207.2 | 229.8 | 199.1 |
| 2245 | 1.2 | | 75.7 | 130.1 | 125.0 | 96.2 | | 181.4 | 233.9 | 319.0 | 198.0 |
| 2247 | 1.2 | | 59.1 | 112.1 | 95.1 | 60.1 | | 202.4 | 281.0 | 339.6 | 215.0 |
| 2174 | 1.2 | | 92.1 | 130.9 | 124.1 | 96.5 | | 98.1 | 164.5 | 251.1 | 92.7 |
| 3313 | 1.2 | | 114.0 | 142.8 | 131.4 | 91.5 | | 132.2 | 158.2 | 201.4 | 72.3 |
| 2230 | 1.2 | | 45.2 | 81.5 | 80.9 | 48.5 | | 256.9 | 347.2 | 285.2 | 330.7 |
| 1432 | 1.2 | | 62.5 | 100.9 | 85.1 | 57.1 | | 134.6 | 223.3 | 199.9 | 198.4 |
| 2238 | 1.2 | | 107.5 | 164.9 | 149.9 | 171.6 | | 108.3 | 228.0 | 219.8 | 140.3 |
| 2169 | 1.3 | | 83.8 | 120.2 | 118.3 | 95.0 | | 209.2 | 281.5 | 205.4 | 242.4 |
| 3493 | 1.3 | | 58.4 | 118.3 | 110.1 | 73.4 | | 76.3 | 140.9 | 252.5 | 97.5 |
| 2772 | 1.3 | | 83.1 | 94.9 | 111.0 | 67.8 | | 127.7 | 163.3 | 211.9 | 144.8 |
| 2769 | 1.3 | | 90.4 | 154.1 | 114.2 | 71.3 | | 232.0 | 274.7 | 188.3 | 299.4 |
| 2257 | 1.3 | | 59.2 | 106.1 | 97.2 | 72.4 | | 228.9 | 319.4 | 455.7 | 241.5 |
| 2226 | 1.3 | | 63.1 | 112.5 | 101.0 | 76.9 | | 292.7 | 363.6 | 312.9 | 347.7 |
| 2229 | 1.3 | | 67.9 | 122.6 | 102.4 | 84.9 | | 336.3 | 385.3 | 322.3 | 485.4 |
| 2170 | 1.3 | | 116.9 | 152.6 | 154.1 | 130.1 | | 257.6 | 331.8 | 287.8 | 296.0 |
| 2689 | 1.3 | | 102.9 | 140.4 | 132.4 | 124.1 | | 257.2 | 372.7 | 344.3 | 330.2 |
| 2510 | 1.4 | | 77.5 | 144.0 | 126.9 | 102.5 | | 167.7 | 251.6 | 293.3 | 211.3 |
| 2670 | 1.4 | | 90.8 | 164.3 | 150.2 | 157.7 | | 427.9 | 508.0 | 472.6 | 616.3 |
| 2669 | 1.4 | | 92.5 | 160.0 | 140.9 | 127.7 | | 147.5 | 250.4 | 268.3 | 185.4 |
| 2219 | 1.4 | | 70.8 | 111.5 | 104.0 | 73.1 | | 208.0 | 332.8 | 334.9 | 272.2 |
| 2667 | 1.4 | | 88.9 | 123.5 | 105.5 | 65.2 | | 150.3 | 221.9 | 260.5 | 171.3 |
| 1188 | 1.4 | | 46.1 | 93.5 | 79.6 | 58.5 | | 108.8 | 176.1 | 192.1 | 133.2 |
| 3489 | 1.4 | | 67.7 | 119.1 | 93.4 | 61.4 | | 206.3 | 301.0 | 367.9 | 247.4 |
| 2765 | 1.4 | | 88.9 | 149.1 | 141.1 | 110.0 | | 117.3 | 229.0 | 219.4 | 121.9 |
| 3295 | 1.4 | | 127.1 | 139.0 | 135.2 | 95.1 | | 263.1 | 279.0 | 309.9 | 243.6 |
| 2762 | 1.4 | | 76.1 | 118.7 | 107.2 | 61.4 | | 138.0 | 175.9 | 195.6 | 124.1 |
| 2682 | 1.4 | | 104.6 | 158.2 | 126.2 | 104.8 | | 211.8 | 278.8 | 292.6 | 234.7 |
| 2271 | 1.4 | | 71.9 | 120.2 | 111.0 | 83.3 | | 173.2 | 275.3 | 251.4 | 209.8 |
| 2497 | 1.5 | | 59.0 | 88.7 | 84.1 | 51.9 | | 129.8 | 186.0 | 210.2 | 100.8 |
| 3301 | 1.5 | | 101.9 | 179.7 | 167.6 | 165.4 | | 152.1 | 230.7 | 312.1 | 167.6 |
| 2545 | 1.5 | | 67.0 | 92.3 | 87.7 | 52.7 | | 235.5 | 317.3 | 378.8 | 293.5 |
| 2241 | 1.5 | | 105.5 | 171.9 | 175.3 | 156.2 | | 332.1 | 401.7 | 290.0 | 356.4 |
| 2764 | 1.5 | | 90.7 | 134.6 | 103.9 | 82.5 | | 316.3 | 335.9 | 264.4 | 424.8 |
| 2696 | 1.5 | | 66.0 | 125.9 | 111.1 | 43.9 | | 190.6 | 260.3 | 198.4 | 209.6 |
| 2184 | 1.5 | | 64.0 | 117.8 | 110.6 | 90.3 | | 182.4 | 330.4 | 214.8 | 284.5 |
| 3333 | 1.5 | | 95.9 | 140.0 | 111.7 | 104.3 | | 121.7 | 234.0 | 248.4 | 153.3 |
| 2252 | 1.5 | | 75.2 | 106.4 | 97.0 | 62.9 | | 225.4 | 296.8 | 336.7 | 306.7 |
| 2288 | 1.5 | | 50.3 | 89.5 | 69.2 | 49.6 | | 193.9 | 248.7 | 257.7 | 185.1 |
| 2551 | 1.5 | | 73.1 | 116.5 | 99.6 | 62.0 | | 224.0 | 258.1 | 405.2 | 248.7 |
| 3490 | 1.5 | | 99.7 | 172.4 | 136.5 | 102.6 | | 139.0 | 217.0 | 302.4 | 118.9 |
| 3292 | 1.5 | | 92.5 | 128.5 | 112.9 | 77.0 | | 231.7 | 271.4 | 403.9 | 255.8 |
| 3312 | 1.5 | | 137.2 | 190.9 | 198.3 | 218.2 | | 322.0 | 426.5 | 444.4 | 423.4 |
| 2194 | 1.5 | | 66.2 | 119.8 | 104.9 | 70.8 | | 129.9 | 211.4 | 205.3 | 142.8 |
| 2175 | 1.6 | | 81.8 | 140.3 | 126.9 | 120.4 | | 168.8 | 257.7 | 280.7 | 211.0 |
| 2534 | 1.6 | | 72.0 | 106.3 | 101.8 | 72.3 | | 237.0 | 312.6 | 457.6 | 275.4 |
| 2269 | 1.6 | | 84.9 | 122.5 | 110.9 | 77.2 | | 458.9 | 438.6 | 516.7 | 431.2 |
| 2190 | 1.6 | | 103.7 | 142.2 | 147.2 | 108.6 | | 114.3 | 163.7 | 202.8 | 64.2 |
| 2480 | 1.6 | | 63.0 | 96.1 | 88.6 | 52.9 | | 204.4 | 305.3 | 271.5 | 224.8 |
| 2178 | 1.6 | | 83.9 | 124.8 | 115.5 | 97.2 | | 229.8 | 270.0 | 264.0 | 222.0 |
| 2196 | 1.6 | | 57.8 | 108.5 | 96.7 | 55.0 | | 155.6 | 211.9 | 215.5 | 160.0 |
| 2272 | 1.6 | | 64.4 | 117.5 | 94.7 | 63.9 | | 261.4 | 347.5 | 377.8 | 355.2 |
| 3304 | 1.7 | | 109.5 | 149.3 | 140.5 | 124.8 | | 312.1 | 372.7 | 436.2 | 418.5 |
| 1316 | 1.7 | | 89.6 | 139.5 | 128.8 | 108.1 | | 150.1 | 243.4 | 248.8 | 169.1 |
| 1551 | 1.7 | | 104.4 | 124.4 | 107.6 | 138.3 | | 310.3 | 332.8 | 253.5 | 325.5 |
| 3099 | 1.7 | | 119.4 | 125.2 | 120.3 | 110.9 | | 226.6 | 297.3 | 327.3 | 249.2 |
| 2687 | 1.7 | | 197.6 | 262.3 | 245.8 | 313.0 | | 133.7 | 244.4 | 328.2 | 156.1 |
| 2296 | 1.7 | | 58.4 | 139.6 | 119.9 | 77.5 | | 174.0 | 230.4 | 279.7 | 231.9 |
| 2179 | 1.8 | | 93.6 | 160.1 | 134.8 | 129.2 | | 148.2 | 228.1 | 198.1 | 165.4 |
| 2274 | 1.8 | | 58.0 | 101.9 | 98.1 | 58.1 | | 178.8 | 236.5 | 312.5 | 191.0 |
| 2524 | 1.8 | | 46.2 | 90.3 | 80.5 | 43.7 | | 310.5 | 291.6 | 396.3 | 267.4 |
| 2511 | 1.8 | | 109.4 | 160.9 | 138.8 | 173.5 | | 167.6 | 257.5 | 243.9 | 212.4 |
| 2214 | 1.8 | | 87.4 | 143.5 | 126.4 | 107.0 | | 328.9 | 386.8 | 333.4 | 417.6 |
| 2260 | 1.8 | | 48.9 | 116.5 | 95.5 | 66.6 | | 256.9 | 365.6 | 361.1 | 311.1 |
| 2542 | 1.9 | | 72.8 | 152.8 | 129.6 | 113.8 | | 135.9 | 234.2 | 224.8 | 185.6 |
| 3332 | 1.9 | | 96.6 | 156.0 | 131.0 | 145.7 | | 210.0 | 286.4 | 322.8 | 222.6 |
| 2209 | 1.9 | | 46.3 | 105.0 | 92.0 | 54.3 | | 464.2 | 476.0 | 545.4 | 490.0 |
| 2535 | 1.9 | | 59.2 | 91.6 | 108.7 | 73.5 | | 215.9 | 306.1 | 359.8 | 258.5 |
| 2509 | 1.9 | | 57.3 | 105.9 | 83.3 | 47.0 | | 361.3 | 421.9 | 454.2 | 460.3 |
| 2467 | 1.9 | | 66.0 | 127.6 | 106.8 | 58.6 | | 208.2 | 325.9 | 288.7 | 250.1 |
| 2188 | 1.9 | | 80.9 | 146.7 | 128.5 | 116.2 | | 162.3 | 261.4 | 178.1 | 182.6 |
| 3492 | 1.9 | | 101.3 | 163.0 | 146.8 | 105.2 | | 160.5 | 257.5 | 286.4 | 140.5 |
| 2228 | 1.9 | | 56.0 | 101.3 | 109.3 | 65.9 | | 102.7 | 172.7 | 167.4 | 110.1 |
| 3098 | 2.0 | | 58.4 | 111.6 | 91.0 | 56.7 | | 296.3 | 384.0 | 423.0 | 399.5 |
| 2544 | 2.0 | | 75.1 | 112.0 | 101.1 | 69.6 | | 257.8 | 385.0 | 400.7 | 372.5 |
| 2676 | 2.0 | | 60.6 | 111.6 | 93.3 | 59.3 | | 165.0 | 319.4 | 254.4 | 251.3 |
| 2536 | 2.0 | | 77.4 | 92.1 | 80.0 | 40.4 | | 363.6 | 378.7 | 394.9 | 436.7 |
| 2299 | 2.0 | | 93.7 | 165.4 | 141.3 | 109.6 | | 108.3 | 168.5 | 267.4 | 109.6 |
| 2304 | 2.0 | | 189.2 | 230.4 | 256.3 | 290.8 | | 111.2 | 164.6 | 306.7 | 116.2 |
| 2751 | 2.0 | | 107.6 | 159.8 | 143.9 | 127.5 | | 177.2 | 248.3 | 287.3 | 210.2 |
| 2276 | 2.0 | | 69.1 | 101.0 | 91.5 | 66.9 | | 326.0 | 432.6 | 503.7 | 473.9 |
| 2681 | 2.1 | | 96.7 | 134.6 | 113.3 | 64.9 | | 188.4 | 187.0 | 378.1 | 131.1 |
| 2248 | 2.1 | | 93.9 | 141.3 | 116.3 | 87.5 | | 264.0 | 315.8 | 425.3 | 280.4 |
| 3278 | 2.1 | | 99.6 | 149.0 | 136.1 | 99.7 | | 104.3 | 183.2 | 276.9 | 152.2 |
| 1747 | 2.1 | | 102.8 | 171.4 | 174.8 | 155.6 | | 461.0 | 577.4 | 660.4 | 711.8 |
| 3320 | 2.1 | | 92.2 | 144.5 | 116.3 | 126.2 | | 187.8 | 276.6 | 218.2 | 218.4 |
| 2183 | 2.2 | | 86.1 | 149.6 | 162.6 | 133.9 | | 513.9 | 700.1 | 629.5 | 885.2 |
| 2767 | 2.2 | | 110.2 | 175.1 | 158.6 | 152.5 | | 206.2 | 237.3 | 263.3 | 212.0 |
| 2231 | 2.2 | | 78.3 | 123.6 | 111.9 | 88.7 | | 453.0 | 578.4 | 506.8 | 631.9 |
| 2182 | 2.2 | | 117.4 | 157.9 | 141.6 | 161.6 | | 120.7 | 193.6 | 175.5 | 131.2 |
| 1766 | 2.2 | | 116.9 | 179.0 | 182.1 | 177.8 | | 278.8 | 385.2 | 406.1 | 363.3 |
| 3319 | 2.2 | | 81.4 | 139.1 | 144.0 | 118.4 | | 545.4 | 506.9 | 657.2 | 657.3 |
| 1401 | 2.2 | | 97.1 | 141.7 | 148.5 | 168.0 | | 128.9 | 183.9 | 182.3 | 133.9 |
| 2302 | 2.2 | | 109.5 | 134.1 | 143.9 | 128.0 | | 101.4 | 154.5 | 214.5 | 106.5 |
| 2204 | 2.2 | | 81.0 | 137.0 | 126.2 | 110.3 | | 309.5 | 418.0 | 352.0 | 417.8 |
| 2259 | 2.2 | | 55.5 | 111.9 | 91.0 | 65.6 | | 176.3 | 284.1 | 187.6 | 218.1 |
| 2546 | 2.3 | | 63.9 | 109.7 | 97.3 | 82.1 | | 186.9 | 293.0 | 299.1 | 201.9 |
| 2468 | 2.3 | | 77.6 | 128.0 | 118.2 | 44.9 | | 194.1 | 222.7 | 323.6 | 270.2 |
| 2685 | 2.3 | | 97.7 | 168.2 | 118.7 | 114.0 | | 472.8 | 675.1 | 514.4 | 717.1 |
| 2195 | 2.3 | | 70.2 | 116.5 | 115.1 | 65.9 | | 290.7 | 367.1 | 340.5 | 345.7 |
| 2191 | 2.3 | | 71.0 | 136.7 | 144.1 | 100.1 | | 180.6 | 283.2 | 267.1 | 250.9 |
| 2282 | 2.3 | | 64.1 | 112.5 | 104.3 | 74.7 | | 164.1 | 210.4 | 273.2 | 176.5 |
| 3100 | 2.4 | | 74.1 | 115.7 | 104.6 | 76.3 | | 146.4 | 241.1 | 233.1 | 173.7 |
| 2516 | 2.4 | | 191.3 | 238.9 | 272.3 | 320.8 | | 160.1 | 239.9 | 277.6 | 164.7 |
| 2262 | 2.4 | | 61.6 | 103.0 | 105.9 | 64.4 | | 199.5 | 318.5 | 311.8 | 218.8 |
| 3310 | 2.4 | | 143.4 | 90.8 | 154.0 | 81.7 | | 195.8 | 118.9 | 226.7 | 98.2 |
| 2237 | 2.4 | | 97.8 | 158.4 | 159.7 | 145.4 | | 122.6 | 209.6 | 249.9 | 130.4 |
| 2234 | 2.4 | | 75.1 | 122.9 | 108.3 | 87.4 | | 218.0 | 289.1 | 241.7 | 251.2 |
| 2208 | 2.4 | | 68.5 | 113.8 | 103.6 | 77.0 | | 273.5 | 390.8 | 326.4 | 398.4 |
| 2286 | 2.5 | | 61.8 | 103.9 | 91.5 | 60.0 | | 521.6 | 504.0 | 599.8 | 601.5 |
| 2251 | 2.5 | | 108.2 | 103.3 | 127.2 | 96.8 | | 222.1 | 304.9 | 414.5 | 275.7 |
| 2192 | 2.6 | | 62.8 | 118.6 | 100.2 | 58.6 | | 217.7 | 300.4 | 333.4 | 279.6 |
| 2292 | 2.6 | | 100.8 | 150.4 | 133.0 | 116.4 | | 405.8 | 436.0 | 412.6 | 514.8 |
| 2244 | 2.6 | | 68.4 | 141.3 | 129.0 | 92.3 | | 118.1 | 194.9 | 333.4 | 157.7 |
| 2185 | 2.6 | | 120.6 | 207.5 | 186.8 | 189.4 | | 332.1 | 421.8 | 375.7 | 396.2 |
| 2671 | 2.6 | | 68.3 | 114.4 | 117.9 | 90.3 | | 227.7 | 309.8 | 337.9 | 262.1 |
| 2275 | 2.7 | | 86.6 | 161.0 | 135.5 | 123.5 | | 291.2 | 384.2 | 392.9 | 448.7 |
| 2265 | 2.7 | | 74.2 | 152.0 | 131.4 | 132.4 | | 266.0 | 307.1 | 330.1 | 251.2 |
| 1299 | 2.8 | | 112.6 | 182.7 | 145.3 | 201.4 | | 283.9 | 389.7 | 488.2 | 372.2 |
| 2306 | 2.8 | | 121.8 | 213.0 | 211.1 | 251.5 | | 420.7 | 508.8 | 493.6 | 574.2 |
| 2181 | 2.8 | | 96.7 | 161.9 | 164.5 | 150.7 | | 547.7 | 608.3 | 381.9 | 708.1 |
| 2679 | 2.9 | | 75.8 | 111.5 | 94.2 | 66.0 | | 239.8 | 281.2 | 296.7 | 305.5 |
| 2550 | 2.9 | | 72.5 | 114.2 | 103.8 | 84.2 | | 163.4 | 285.6 | 249.0 | 214.2 |
| 2278 | 3.0 | | 73.6 | 111.1 | 101.4 | 59.1 | | 676.6 | 740.5 | 844.9 | 893.0 |
| 2756 | 3.0 | | 100.7 | 140.9 | 127.4 | 101.4 | | 327.6 | 402.9 | 369.5 | 341.5 |
| 3487 | 3.0 | | 89.3 | 131.6 | 130.1 | 109.8 | | 121.8 | 180.1 | 269.8 | 102.3 |
| 2688 | 3.1 | | 230.5 | 290.2 | 291.5 | 420.0 | | 909.4 | 1040.3 | 1690.6 | 1534.9 |
| 2227 | 3.1 | | 99.6 | 146.4 | 138.1 | 127.1 | | 389.0 | 458.6 | 453.2 | 482.2 |
| 2525 | 3.2 | | 47.9 | 106.6 | 98.0 | 39.5 | | 306.9 | 369.8 | 443.1 | 430.6 |
| 2771 | 3.4 | | 153.3 | 252.3 | 240.1 | 261.8 | | 528.5 | 649.8 | 797.5 | 649.3 |
| 2752 | 3.6 | | 120.8 | 188.5 | 185.4 | 195.8 | | 165.7 | 235.8 | 322.8 | 171.0 |
| 2695 | 3.6 | | 78.8 | 146.9 | 118.2 | 97.1 | | 218.7 | 345.6 | 367.6 | 334.7 |
| 2263 | 3.6 | | 74.8 | 134.5 | 122.9 | 112.4 | | 196.4 | 287.0 | 327.7 | 207.4 |
| 2273 | 3.7 | | 58.0 | 109.0 | 88.4 | 51.0 | | 203.1 | 253.9 | 296.7 | 260.9 |
| 3488 | 3.7 | | 92.4 | 147.3 | 130.9 | 114.1 | | 169.4 | 296.7 | 274.0 | 223.8 |
| 2661 | 4.0 | | 91.2 | 130.2 | 112.8 | 89.2 | | 278.7 | 434.0 | 275.9 | 368.0 |
| 2201 | 4.0 | | 107.4 | 156.4 | 138.9 | 126.8 | | 532.7 | 517.1 | 438.9 | 507.3 |
| 2261 | 4.2 | | 87.7 | 149.8 | 133.7 | 138.7 | | 557.2 | 605.0 | 660.4 | 695.3 |
| 2203 | 4.2 | | 70.2 | 110.3 | 86.1 | 60.0 | | 479.5 | 534.8 | 499.4 | 573.5 |
| 2193 | 4.2 | | 104.4 | 153.3 | 150.7 | 129.3 | | 405.3 | 471.2 | 454.1 | 451.1 |
| 3328 | 4.3 | | 169.8 | 200.4 | 201.9 | 209.2 | | 139.3 | 230.8 | 260.3 | 185.9 |
| 3484 | 4.5 | | 120.5 | 221.9 | 185.6 | 219.5 | | 158.3 | 276.7 | 302.3 | 184.0 |
| 2202 | 4.6 | | 75.7 | 109.8 | 109.4 | 84.9 | | 109.1 | 180.2 | 242.6 | 131.1 |
| 2236 | 4.6 | | 77.2 | 126.9 | 109.1 | 88.8 | | 391.2 | 514.1 | 502.6 | 518.7 |
| 3097 | 4.8 | | 67.0 | 121.2 | 118.3 | 90.0 | | 106.9 | 191.0 | 343.5 | 129.6 |
| 2284 | 4.8 | | 131.6 | 222.5 | 239.5 | 258.7 | | 488.0 | 631.3 | 867.4 | 784.7 |
| 2253 | 4.9 | | 64.1 | 100.4 | 102.4 | 77.6 | | 376.4 | 423.8 | 562.0 | 426.6 |
| 2498 | 5.0 | | 113.0 | 147.2 | 132.1 | 144.6 | | 756.8 | 728.5 | 943.7 | 996.3 |
| 2466 | 5.8 | | 74.9 | 101.7 | 107.8 | 61.4 | | 285.6 | 345.1 | 383.4 | 376.2 |
| 2206 | 6.0 | | 146.4 | 233.0 | 244.7 | 290.4 | | 686.4 | 789.0 | 748.8 | 994.6 |
| 2327 | 6.0 | | 56.3 | 101.5 | 99.8 | 63.3 | | 378.1 | 463.0 | 423.3 | 542.1 |
| 2774 | 6.1 | | 142.5 | 244.7 | 269.7 | 286.6 | | 397.0 | 584.8 | 766.1 | 733.3 |
| 2210 | 6.3 | | 64.5 | 95.3 | 78.9 | 43.9 | | 345.8 | 473.9 | 338.6 | 482.5 |
| 2268 | 6.4 | | 84.7 | 125.8 | 126.8 | 91.4 | | 333.7 | 421.7 | 473.0 | 370.4 |
| 2239 | 7.9 | | 92.1 | 170.8 | 166.6 | 138.7 | | 389.6 | 509.5 | 397.7 | 615.2 |
| 2250 | 10.2 | | 63.0 | 132.5 | 124.1 | 101.2 | | 729.6 | 789.4 | 833.0 | 1075.2 |
| 2205 | 11.5 | | 116.4 | 192.2 | 169.1 | 189.5 | | 779.5 | 811.2 | 1003.7 | 1483.5 |
| 2754 | 28.6 | | 86.4 | 125.3 | 129.3 | 87.0 | | 1204.1 | 1162.3 | 1179.5 | 1930.9 |
| 2665 | 481.7 | | 115.6 | 184.2 | 188.7 | 170.8 | | 887.8 | 878.1 | 1022.7 | 1517.6 |

|  | **203056_s_at** | | | | **216433_s_at** | | | |
| --- | --- | --- | --- | --- | --- | --- | --- | --- |
| **Sample** | **MAS** | **dChip** | **RMA** | **GCRMA** | **MAS** | **dChip** | **RMA** | **GCRMA** |
| 3101 | 111.3 | 166.5 | 297.1 | 231.7 | 7.7 | 34.3 | 18.9 | 9.7 |
| 2200 | 173.3 | 170.3 | 463.2 | 295.9 | 21.5 | 37.8 | 28.0 | 27.5 |
| 2207 | 90.8 | 95.5 | 253.1 | 121.9 | 9.9 | 60.8 | 20.9 | 10.9 |
| 3482 | 157.6 | 235.7 | 545.5 | 361.2 | 12.1 | 30.4 | 22.8 | 11.3 |
| 2235 | 55.3 | 100.8 | 294.8 | 110.7 | 0.3 | 32.2 | 17.6 | 8.7 |
| 2225 | 102.4 | 170.4 | 462.7 | 216.6 | 9.1 | 32.2 | 20.1 | 9.3 |
| 2514 | 92.9 | 105.6 | 187.8 | 152.2 | 7.2 | 51.0 | 20.5 | 11.9 |
| 2766 | 64.4 | 88.2 | 227.9 | 120.1 | 1.1 | 14.8 | 18.5 | 8.4 |
| 3324 | 81.9 | 102.6 | 358.9 | 144.6 | 2.1 | 42.1 | 19.9 | 9.8 |
| 2267 | 116.5 | 158.6 | 289.9 | 227.9 | 8.2 | 20.3 | 27.6 | 13.1 |
| 3325 | 95.0 | 102.7 | 338.9 | 153.5 | 12.6 | 0.9 | 20.7 | 10.8 |
| 2704 | 183.3 | 197.9 | 373.7 | 315.8 | 1.0 | 16.5 | 19.4 | 11.0 |
| 1482 | 50.0 | 146.0 | 304.3 | 178.9 | 5.4 | 34.2 | 19.6 | 10.8 |
| 3316 | 60.1 | 95.4 | 271.8 | 102.5 | 2.2 | 44.6 | 17.9 | 10.3 |
| 2291 | 87.6 | 107.9 | 278.2 | 149.8 | 10.5 | 34.1 | 21.1 | 11.3 |
| 2216 | 71.8 | 100.4 | 305.3 | 136.1 | 9.2 | 38.5 | 19.2 | 10.7 |
| 3314 | 122.6 | 155.2 | 360.6 | 211.2 | 11.9 | 54.2 | 25.3 | 11.8 |
| 2270 | 178.3 | 242.1 | 700.7 | 429.1 | 27.4 | 72.7 | 36.9 | 53.8 |
| 2656 | 81.7 | 110.8 | 332.7 | 138.1 | 2.1 | 29.6 | 17.1 | 7.7 |
| 2285 | 85.8 | 120.2 | 236.3 | 173.7 | 5.7 | 50.5 | 21.1 | 10.3 |
| 3327 | 94.3 | 103.3 | 326.2 | 135.6 | 5.3 | 41.7 | 20.7 | 11.5 |
| 3485 | 103.5 | 130.2 | 191.0 | 168.5 | 3.1 | 24.4 | 18.7 | 11.7 |
| 1063 | 86.5 | 104.1 | 245.6 | 122.5 | 2.5 | 33.3 | 20.0 | 11.1 |
| 1595 | 85.1 | 106.4 | 295.0 | 141.8 | 6.1 | 9.4 | 17.6 | 7.2 |
| 2171 | 70.7 | 97.6 | 284.9 | 123.9 | 5.4 | 40.0 | 19.5 | 10.0 |
| 2662 | 147.7 | 190.0 | 267.2 | 267.7 | 5.6 | 19.1 | 18.7 | 11.6 |
| 2655 | 154.3 | 197.0 | 346.1 | 288.8 | 4.2 | 20.7 | 19.1 | 10.2 |
| 3318 | 112.4 | 125.0 | 320.7 | 204.5 | 6.2 | 15.0 | 18.6 | 10.0 |
| 3326 | 72.2 | 90.2 | 270.2 | 108.0 | 5.2 | 29.3 | 17.5 | 8.4 |
| 3279 | 52.7 | 83.8 | 314.3 | 101.7 | 0.6 | 20.2 | 16.0 | 7.0 |
| 3317 | 91.0 | 114.7 | 246.4 | 145.8 | 5.0 | 31.6 | 18.9 | 10.6 |
| 2233 | 53.8 | 85.1 | 243.7 | 76.4 | 2.3 | 21.7 | 18.1 | 6.5 |
| 2694 | 145.5 | 163.8 | 468.9 | 278.8 | 28.5 | 10.5 | 21.6 | 12.1 |
| 2749 | 95.6 | 129.1 | 343.7 | 190.8 | 2.8 | 28.3 | 20.8 | 10.7 |
| 2668 | 61.2 | 84.2 | 214.2 | 71.6 | 4.3 | 25.7 | 17.1 | 9.2 |
| 2256 | 112.2 | 159.1 | 403.1 | 233.3 | 2.4 | 35.3 | 19.6 | 10.4 |
| 2490 | 127.6 | 168.3 | 407.3 | 276.5 | 2.3 | 37.5 | 19.6 | 11.1 |
| 2220 | 93.3 | 150.1 | 349.1 | 197.9 | 2.0 | 26.5 | 18.3 | 10.3 |
| 3331 | 117.7 | 173.1 | 571.6 | 299.8 | 15.1 | 58.0 | 23.6 | 11.3 |
| 3322 | 66.2 | 84.0 | 231.8 | 116.7 | 10.4 | 17.9 | 19.5 | 11.1 |
| 2290 | 99.3 | 123.5 | 343.6 | 162.9 | 10.7 | 35.3 | 17.8 | 9.4 |
| 3315 | 67.3 | 89.0 | 221.5 | 115.2 | 4.1 | 37.0 | 22.8 | 13.7 |
| 2254 | 91.5 | 135.3 | 267.3 | 187.0 | 7.1 | 49.6 | 20.7 | 10.4 |
| 2224 | 102.4 | 185.7 | 531.4 | 278.8 | 5.5 | 18.8 | 17.9 | 9.9 |
| 2680 | 93.7 | 114.1 | 319.0 | 145.5 | 6.1 | 39.0 | 19.4 | 10.9 |
| 3285 | 60.0 | 83.1 | 232.7 | 95.6 | 10.0 | 17.1 | 19.2 | 11.4 |
| 2678 | 104.0 | 139.3 | 380.3 | 203.5 | 2.5 | 18.2 | 18.5 | 9.5 |
| 2672 | 75.3 | 105.1 | 280.8 | 134.9 | 1.2 | 11.4 | 18.2 | 10.1 |
| 2287 | 89.8 | 133.5 | 231.3 | 188.0 | 11.0 | 45.0 | 21.2 | 10.6 |
| 322 | 73.7 | 129.1 | 301.4 | 165.9 | 10.6 | 31.0 | 22.5 | 13.4 |
| 3115 | 134.6 | 164.1 | 305.6 | 236.5 | 1.9 | 31.9 | 19.4 | 11.2 |
| 2647 | 142.1 | 163.3 | 374.2 | 231.0 | 0.8 | 14.7 | 18.0 | 8.9 |
| 3309 | 95.0 | 111.5 | 283.6 | 180.8 | 9.8 | 14.4 | 16.3 | 7.3 |
| 3102 | 90.3 | 108.1 | 233.8 | 139.8 | 7.5 | 35.1 | 19.9 | 10.4 |
| 3334 | 146.5 | 197.8 | 331.9 | 299.9 | 5.1 | 32.3 | 18.0 | 7.2 |
| 3293 | 113.5 | 142.5 | 366.0 | 166.2 | 1.3 | 27.9 | 18.4 | 10.7 |
| 2240 | 79.6 | 102.6 | 215.0 | 122.2 | 2.8 | 26.3 | 21.4 | 10.4 |
| 3308 | 99.3 | 129.0 | 342.2 | 164.8 | 14.8 | 35.9 | 19.4 | 10.7 |
| 2539 | 90.8 | 113.2 | 304.7 | 148.1 | 1.1 | 14.8 | 18.4 | 8.4 |
| 2326 | 66.4 | 104.4 | 277.1 | 95.0 | 0.3 | 39.4 | 18.1 | 10.3 |
| 3329 | 73.0 | 97.3 | 278.3 | 120.3 | 2.8 | 18.0 | 18.8 | 10.2 |
| 3096 | 146.3 | 229.0 | 503.1 | 351.3 | 8.0 | 24.0 | 18.9 | 10.1 |
| 3277 | 94.2 | 134.7 | 253.6 | 177.0 | 8.0 | 31.2 | 20.8 | 10.5 |
| 2307 | 125.1 | 131.9 | 210.9 | 224.2 | 5.5 | 69.2 | 23.3 | 12.5 |
| 2243 | 73.1 | 141.2 | 330.5 | 180.5 | 2.4 | 11.6 | 17.4 | 10.4 |
| 2217 | 72.0 | 112.3 | 316.1 | 129.2 | 6.9 | 28.3 | 20.7 | 9.9 |
| 2222 | 96.8 | 117.0 | 289.3 | 147.2 | 1.4 | 33.6 | 19.4 | 11.2 |
| 2212 | 99.0 | 137.6 | 384.2 | 201.4 | 10.4 | 28.4 | 21.6 | 10.8 |
| 3486 | 77.2 | 108.2 | 143.6 | 136.7 | 7.2 | 27.5 | 24.2 | 11.4 |
| 2750 | 69.9 | 125.8 | 332.9 | 170.2 | 0.9 | 26.2 | 19.6 | 11.0 |
| 1201 | 104.9 | 161.7 | 396.2 | 186.6 | 13.9 | 45.5 | 24.0 | 13.7 |
| 2658 | 121.9 | 163.8 | 420.5 | 219.2 | 13.2 | 50.4 | 20.6 | 10.9 |
| 2246 | 164.5 | 202.3 | 423.6 | 297.2 | 6.1 | 35.5 | 22.0 | 9.4 |
| 2549 | 116.3 | 135.8 | 334.2 | 201.0 | 10.9 | 29.4 | 21.4 | 10.9 |
| 3321 | 89.5 | 117.5 | 312.0 | 144.8 | 2.2 | 37.5 | 20.4 | 10.5 |
| 2683 | 118.0 | 120.7 | 301.4 | 188.0 | 13.8 | 39.2 | 20.6 | 13.6 |
| 3330 | 70.1 | 59.9 | 299.4 | 73.5 | 11.1 | 21.9 | 19.6 | 10.7 |
| 2301 | 107.7 | 117.1 | 357.3 | 175.7 | 14.8 | 30.6 | 22.2 | 11.4 |
| 2757 | 98.8 | 113.9 | 243.6 | 139.9 | 1.2 | 22.6 | 18.1 | 11.2 |
| 3491 | 104.6 | 123.1 | 292.1 | 172.4 | 11.2 | 28.6 | 18.8 | 9.4 |
| 2176 | 83.1 | 126.5 | 296.7 | 180.3 | 12.7 | 26.6 | 20.7 | 9.6 |
| 2543 | 132.7 | 144.8 | 293.0 | 196.9 | 2.6 | 21.1 | 16.5 | 10.4 |
| 2507 | 114.0 | 156.2 | 359.3 | 237.1 | 5.8 | 25.6 | 20.7 | 10.5 |
| 2197 | 93.3 | 126.2 | 293.3 | 168.3 | 2.0 | 41.3 | 19.8 | 10.2 |
| 2686 | 88.3 | 97.1 | 225.6 | 128.1 | 4.6 | 0.9 | 18.0 | 11.0 |
| 2748 | 76.6 | 134.3 | 341.5 | 171.6 | 2.3 | 38.2 | 17.5 | 8.2 |
| 3289 | 292.0 | 364.6 | 908.0 | 784.1 | 14.0 | 47.2 | 20.7 | 11.2 |
| 3286 | 84.2 | 113.8 | 313.2 | 142.0 | 1.8 | 38.5 | 18.4 | 8.8 |
| 2187 | 86.2 | 103.5 | 280.6 | 119.1 | 2.6 | 12.6 | 19.4 | 9.3 |
| 2753 | 98.1 | 151.5 | 424.8 | 221.4 | 17.9 | 56.7 | 20.7 | 11.3 |
| 2541 | 53.7 | 125.3 | 321.1 | 216.3 | 14.5 | 25.8 | 21.5 | 11.8 |
| 2242 | 89.4 | 94.5 | 291.1 | 132.0 | 12.0 | 26.8 | 19.9 | 10.8 |
| 3481 | 143.7 | 153.9 | 208.9 | 253.3 | 1.0 | 36.1 | 18.2 | 7.1 |
| 1448 | 75.3 | 93.7 | 203.7 | 120.5 | 10.7 | 45.3 | 21.5 | 13.5 |
| 1197 | 86.9 | 91.9 | 260.8 | 123.7 | 14.1 | 47.5 | 22.5 | 12.2 |
| 2692 | 215.4 | 232.8 | 564.6 | 396.7 | 20.1 | 26.5 | 24.3 | 13.6 |
| 2300 | 103.9 | 119.1 | 266.8 | 165.9 | 4.3 | 30.1 | 19.9 | 13.0 |
| 2281 | 96.6 | 137.1 | 431.7 | 211.1 | 16.3 | 42.0 | 22.0 | 10.8 |
| 2199 | 106.7 | 113.3 | 404.7 | 166.9 | 6.5 | 13.9 | 21.4 | 11.6 |
| 2249 | 112.2 | 168.4 | 297.5 | 235.0 | 18.9 | 41.6 | 21.2 | 12.9 |
| 2305 | 55.9 | 76.8 | 182.7 | 104.0 | 1.3 | 39.3 | 20.4 | 11.5 |
| 2218 | 100.2 | 170.4 | 411.5 | 214.3 | 5.3 | 36.8 | 19.0 | 10.4 |
| 3311 | 84.2 | 142.9 | 326.6 | 205.1 | 0.6 | 19.3 | 18.9 | 6.5 |
| 3323 | 89.3 | 103.6 | 218.5 | 111.2 | 2.2 | 21.7 | 20.4 | 9.8 |
| 2189 | 94.5 | 136.1 | 316.2 | 167.5 | 1.8 | 36.9 | 19.4 | 7.5 |
| 3483 | 69.4 | 124.5 | 300.5 | 166.2 | 1.4 | 18.5 | 18.5 | 10.9 |
| 2644 | 282.0 | 242.4 | 977.7 | 468.5 | 30.0 | 48.2 | 25.1 | 31.0 |
| 2198 | 95.6 | 123.6 | 394.6 | 193.2 | 13.0 | 28.9 | 22.4 | 11.3 |
| 2747 | 135.4 | 164.5 | 446.4 | 242.0 | 9.1 | 27.9 | 19.5 | 10.5 |
| 2186 | 83.1 | 155.6 | 565.4 | 194.6 | 6.4 | 10.4 | 20.6 | 10.1 |
| 2666 | 93.1 | 105.5 | 299.9 | 121.8 | 7.7 | 19.3 | 17.3 | 8.4 |
| 1174 | 66.5 | 111.0 | 347.8 | 156.6 | 11.6 | 33.8 | 19.7 | 10.3 |
| 2245 | 123.4 | 156.6 | 338.2 | 255.4 | 8.4 | 26.0 | 19.4 | 10.6 |
| 2247 | 131.0 | 180.4 | 354.1 | 256.4 | 7.6 | 30.9 | 18.9 | 10.1 |
| 2174 | 79.9 | 111.8 | 271.0 | 161.2 | 18.0 | 20.9 | 22.3 | 11.7 |
| 3313 | 78.8 | 100.4 | 234.7 | 152.9 | 13.6 | 33.9 | 23.6 | 18.4 |
| 2230 | 103.6 | 181.4 | 484.9 | 220.6 | 2.8 | 29.0 | 20.1 | 10.6 |
| 1432 | 75.3 | 123.9 | 327.9 | 143.4 | 7.8 | 31.3 | 21.8 | 10.6 |
| 2238 | 87.4 | 117.2 | 270.6 | 138.2 | 8.1 | 27.4 | 19.0 | 10.5 |
| 2169 | 75.7 | 111.3 | 375.6 | 138.0 | 8.5 | 33.0 | 20.1 | 10.4 |
| 3493 | 87.2 | 119.0 | 216.8 | 177.9 | 2.9 | 16.5 | 19.0 | 9.0 |
| 2772 | 89.7 | 126.5 | 233.7 | 135.6 | 5.4 | 24.2 | 18.1 | 11.3 |
| 2769 | 94.0 | 118.5 | 442.3 | 139.3 | 11.2 | 18.9 | 20.5 | 11.9 |
| 2257 | 149.3 | 229.9 | 370.2 | 354.1 | 6.6 | 24.1 | 22.4 | 12.4 |
| 2226 | 104.8 | 149.3 | 488.1 | 227.6 | 14.9 | 29.7 | 22.6 | 17.5 |
| 2229 | 114.3 | 161.3 | 494.7 | 262.8 | 8.9 | 32.4 | 20.4 | 10.3 |
| 2170 | 127.1 | 145.9 | 463.1 | 239.0 | 6.7 | 18.8 | 18.3 | 10.6 |
| 2689 | 156.6 | 164.6 | 429.8 | 326.1 | 16.4 | 19.4 | 21.3 | 11.9 |
| 2510 | 107.6 | 162.3 | 344.3 | 246.1 | 2.3 | 20.9 | 18.3 | 10.6 |
| 2670 | 179.6 | 272.4 | 720.1 | 400.6 | 5.6 | 32.0 | 19.2 | 9.8 |
| 2669 | 135.2 | 166.3 | 308.0 | 190.8 | 2.0 | 3.7 | 18.9 | 10.5 |
| 2219 | 125.1 | 199.7 | 442.7 | 249.0 | 7.2 | -1.5 | 18.8 | 10.7 |
| 2667 | 115.1 | 144.7 | 320.8 | 210.9 | 6.5 | 30.0 | 19.8 | 11.4 |
| 1188 | 68.8 | 113.2 | 250.8 | 149.8 | 6.2 | 63.6 | 18.0 | 10.3 |
| 3489 | 134.5 | 191.7 | 394.4 | 281.2 | 14.9 | 44.5 | 21.3 | 10.7 |
| 2765 | 107.9 | 126.1 | 251.1 | 151.2 | 5.0 | 26.8 | 21.2 | 10.9 |
| 3295 | 166.7 | 193.0 | 349.1 | 242.0 | 0.7 | 36.2 | 19.4 | 11.4 |
| 2762 | 101.2 | 113.8 | 261.3 | 142.2 | 3.4 | 14.0 | 20.5 | 12.0 |
| 2682 | 135.6 | 151.6 | 392.7 | 236.6 | 3.6 | 27.5 | 19.4 | 11.2 |
| 2271 | 86.3 | 115.0 | 361.0 | 181.9 | 12.4 | 31.8 | 20.5 | 10.4 |
| 2497 | 112.1 | 115.4 | 219.5 | 157.6 | 2.6 | 45.3 | 19.1 | 11.7 |
| 3301 | 158.3 | 158.5 | 305.1 | 226.8 | 2.5 | 19.6 | 18.6 | 8.7 |
| 2545 | 119.6 | 173.6 | 445.2 | 304.4 | 6.9 | 25.6 | 21.7 | 11.8 |
| 2241 | 104.0 | 160.9 | 537.0 | 236.6 | 8.2 | 44.6 | 20.8 | 10.3 |
| 2764 | 124.4 | 144.4 | 546.7 | 192.4 | 3.3 | 22.7 | 18.4 | 12.0 |
| 2696 | 88.4 | 95.7 | 363.7 | 157.8 | 1.6 | 29.3 | 17.5 | 11.3 |
| 2184 | 80.3 | 122.0 | 405.3 | 152.2 | 4.3 | 15.8 | 19.9 | 9.8 |
| 3333 | 90.9 | 141.9 | 282.8 | 193.8 | 1.5 | 12.4 | 17.0 | 8.9 |
| 2252 | 158.4 | 189.4 | 440.9 | 299.1 | 2.1 | 23.2 | 21.2 | 10.9 |
| 2288 | 117.1 | 139.6 | 318.5 | 194.5 | 8.2 | 27.0 | 20.9 | 10.5 |
| 2551 | 191.9 | 219.2 | 392.1 | 340.6 | 2.3 | 5.1 | 21.6 | 12.2 |
| 3490 | 127.4 | 150.8 | 309.1 | 213.5 | 8.2 | 6.2 | 21.4 | 12.0 |
| 3292 | 207.4 | 189.4 | 437.7 | 377.8 | 13.3 | 37.9 | 21.4 | 12.2 |
| 3312 | 156.5 | 243.9 | 590.9 | 401.8 | 11.5 | 45.8 | 22.4 | 11.5 |
| 2194 | 81.7 | 102.9 | 284.3 | 142.8 | 12.6 | 26.0 | 21.6 | 13.6 |
| 2175 | 98.2 | 131.3 | 342.2 | 203.5 | 8.4 | 38.3 | 21.0 | 10.0 |
| 2534 | 164.2 | 210.1 | 408.5 | 392.6 | 10.0 | 39.3 | 23.0 | 12.3 |
| 2269 | 225.2 | 214.8 | 614.5 | 466.7 | 4.0 | 18.4 | 22.1 | 13.2 |
| 2190 | 76.3 | 115.4 | 211.0 | 142.5 | 8.9 | 42.9 | 22.1 | 11.6 |
| 2480 | 117.7 | 155.7 | 389.4 | 206.8 | 10.0 | 28.0 | 19.0 | 10.5 |
| 2178 | 112.5 | 141.4 | 376.2 | 195.2 | 0.9 | 32.5 | 19.3 | 10.8 |
| 2196 | 77.1 | 111.2 | 306.1 | 139.4 | 3.9 | 18.0 | 18.7 | 8.6 |
| 2272 | 168.7 | 197.5 | 489.9 | 380.8 | 35.0 | 44.4 | 39.0 | 41.8 |
| 3304 | 204.9 | 222.7 | 586.0 | 421.0 | 13.2 | 37.2 | 22.4 | 14.2 |
| 1316 | 80.1 | 140.6 | 302.3 | 182.6 | 14.2 | 23.6 | 22.3 | 11.1 |
| 1551 | 136.5 | 155.2 | 484.8 | 217.9 | 14.8 | 41.3 | 20.6 | 10.4 |
| 3099 | 153.9 | 136.5 | 444.9 | 275.0 | 11.8 | 15.4 | 20.6 | 12.7 |
| 2687 | 148.0 | 167.0 | 288.4 | 293.4 | 14.5 | 6.2 | 24.3 | 12.4 |
| 2296 | 107.5 | 124.1 | 358.2 | 215.9 | 8.8 | 40.0 | 21.6 | 12.6 |
| 2179 | 75.8 | 106.2 | 308.0 | 124.7 | 0.9 | 26.7 | 17.4 | 8.4 |
| 2274 | 139.4 | 137.9 | 353.0 | 250.6 | 15.5 | 48.7 | 24.0 | 11.3 |
| 2524 | 135.3 | 193.4 | 412.4 | 328.2 | 3.8 | 25.6 | 17.1 | 9.4 |
| 2511 | 106.4 | 141.3 | 357.5 | 181.8 | 20.7 | 31.6 | 21.8 | 13.4 |
| 2214 | 126.7 | 173.2 | 586.6 | 266.1 | 17.6 | 57.6 | 29.0 | 21.8 |
| 2260 | 128.1 | 195.2 | 478.7 | 293.2 | 16.9 | 51.9 | 26.3 | 20.5 |
| 2542 | 81.9 | 126.0 | 310.0 | 159.8 | 4.5 | 16.2 | 18.1 | 8.4 |
| 3332 | 111.4 | 171.2 | 350.8 | 261.3 | 4.9 | 23.2 | 21.9 | 9.0 |
| 2209 | 223.5 | 265.8 | 790.0 | 493.4 | 19.5 | 35.6 | 26.2 | 15.7 |
| 2535 | 128.3 | 165.7 | 381.9 | 278.3 | 10.6 | 38.9 | 20.8 | 10.9 |
| 2509 | 139.4 | 223.4 | 616.3 | 393.9 | 4.7 | 15.6 | 20.6 | 11.7 |
| 2467 | 138.5 | 152.6 | 389.3 | 235.3 | 7.8 | 21.8 | 19.9 | 11.0 |
| 2188 | 71.0 | 111.7 | 327.2 | 115.8 | 5.4 | 23.1 | 16.9 | 8.8 |
| 3492 | 115.6 | 155.5 | 304.4 | 230.1 | 6.0 | 7.4 | 21.4 | 11.4 |
| 2228 | 61.1 | 95.7 | 198.7 | 111.8 | 6.0 | 23.2 | 19.2 | 9.7 |
| 3098 | 152.8 | 212.2 | 564.2 | 371.3 | 11.9 | 41.8 | 20.7 | 13.2 |
| 2544 | 129.8 | 199.2 | 476.8 | 370.4 | 17.7 | 19.7 | 21.4 | 11.5 |
| 2676 | 114.8 | 127.7 | 385.3 | 178.3 | 5.7 | 13.9 | 19.1 | 11.3 |
| 2536 | 210.2 | 208.8 | 570.8 | 356.2 | 4.8 | 23.9 | 21.8 | 12.5 |
| 2299 | 118.9 | 153.0 | 223.0 | 210.8 | 16.6 | 33.6 | 22.3 | 17.8 |
| 2304 | 153.8 | 144.7 | 261.2 | 223.9 | 15.2 | 21.5 | 21.6 | 12.4 |
| 2751 | 123.1 | 145.8 | 341.2 | 204.6 | 7.1 | 44.2 | 20.7 | 11.3 |
| 2276 | 173.8 | 260.6 | 657.3 | 478.7 | 30.3 | 51.8 | 32.7 | 28.0 |
| 2681 | 194.0 | 227.3 | 313.2 | 356.3 | 2.9 | 23.8 | 20.1 | 14.8 |
| 2248 | 132.9 | 194.2 | 422.9 | 356.6 | 20.1 | 27.7 | 23.3 | 20.8 |
| 3278 | 77.9 | 135.8 | 261.5 | 214.0 | 5.4 | 29.9 | 18.1 | 10.7 |
| 1747 | 234.2 | 296.2 | 907.7 | 563.4 | 40.2 | 47.9 | 37.4 | 28.6 |
| 3320 | 81.1 | 109.6 | 311.0 | 161.2 | 6.0 | 3.7 | 20.3 | 11.1 |
| 2183 | 245.8 | 331.2 | 1111.0 | 586.6 | 20.3 | 39.3 | 25.4 | 14.0 |
| 2767 | 112.8 | 147.9 | 389.1 | 181.5 | 5.4 | 26.5 | 21.2 | 12.8 |
| 2231 | 186.9 | 272.0 | 775.5 | 479.9 | 9.6 | 17.2 | 21.6 | 12.7 |
| 2182 | 67.7 | 105.3 | 262.2 | 122.1 | 3.5 | 13.6 | 18.6 | 10.3 |
| 1766 | 141.1 | 205.2 | 525.1 | 370.7 | 2.4 | 14.3 | 19.3 | 10.4 |
| 3319 | 264.9 | 274.4 | 879.6 | 635.4 | 31.1 | 66.1 | 25.8 | 36.6 |
| 1401 | 62.0 | 95.8 | 251.4 | 117.4 | 8.9 | 28.4 | 19.1 | 10.2 |
| 2302 | 91.5 | 111.6 | 271.2 | 139.9 | 2.1 | 18.6 | 19.8 | 10.9 |
| 2204 | 140.3 | 186.8 | 597.3 | 300.2 | 22.4 | 45.3 | 25.1 | 14.6 |
| 2259 | 72.2 | 112.2 | 336.9 | 134.2 | 0.4 | 28.9 | 18.9 | 10.1 |
| 2546 | 135.1 | 162.5 | 359.2 | 246.6 | 9.9 | 40.8 | 22.9 | 12.7 |
| 2468 | 122.1 | 179.7 | 380.3 | 291.3 | 4.3 | 25.6 | 19.0 | 12.6 |
| 2685 | 204.7 | 265.9 | 811.7 | 544.1 | 15.7 | 37.2 | 21.9 | 12.6 |
| 2195 | 147.8 | 176.3 | 516.9 | 295.3 | 14.0 | 30.7 | 25.5 | 15.5 |
| 2191 | 118.5 | 133.7 | 383.7 | 212.9 | 6.6 | 30.6 | 22.0 | 11.2 |
| 2282 | 111.1 | 130.5 | 337.0 | 220.6 | 16.3 | 25.4 | 23.1 | 12.1 |
| 3100 | 68.8 | 127.7 | 314.7 | 159.0 | 2.9 | 24.1 | 19.1 | 9.8 |
| 2516 | 152.5 | 162.0 | 274.7 | 210.9 | 3.0 | 23.3 | 22.8 | 11.9 |
| 2262 | 122.8 | 156.6 | 367.1 | 232.1 | 9.6 | 35.0 | 21.7 | 10.8 |
| 3310 | 121.6 | 65.9 | 158.6 | 110.9 | 7.9 | -0.9 | 23.2 | 13.5 |
| 2237 | 97.0 | 145.9 | 247.7 | 192.0 | 11.0 | 33.5 | 19.0 | 10.0 |
| 2234 | 91.9 | 132.6 | 404.4 | 189.0 | 13.3 | 27.4 | 20.4 | 10.4 |
| 2208 | 133.6 | 192.0 | 518.2 | 265.3 | 9.5 | 57.9 | 21.0 | 10.7 |
| 2286 | 316.6 | 317.9 | 735.2 | 628.4 | 58.7 | 73.3 | 41.7 | 98.2 |
| 2251 | 192.6 | 215.0 | 394.8 | 409.2 | 10.1 | 41.8 | 20.9 | 14.0 |
| 2192 | 118.9 | 176.5 | 385.9 | 282.4 | 7.9 | 43.5 | 22.6 | 14.2 |
| 2292 | 166.0 | 198.3 | 740.0 | 328.0 | 33.4 | 45.5 | 31.6 | 29.7 |
| 2244 | 102.0 | 182.6 | 280.3 | 261.5 | 2.0 | 12.8 | 21.0 | 10.7 |
| 2185 | 144.7 | 195.0 | 534.2 | 284.5 | 12.7 | 20.5 | 22.0 | 9.6 |
| 2671 | 121.6 | 173.9 | 424.4 | 267.7 | 2.1 | 42.7 | 19.4 | 10.1 |
| 2275 | 167.9 | 187.7 | 598.9 | 331.2 | 24.5 | 60.9 | 31.1 | 27.7 |
| 2265 | 124.7 | 155.3 | 384.7 | 245.9 | 6.5 | 19.9 | 21.1 | 11.0 |
| 1299 | 192.0 | 262.5 | 527.9 | 403.5 | 11.6 | 48.4 | 23.7 | 14.9 |
| 2306 | 160.5 | 251.6 | 773.1 | 402.6 | 19.5 | 50.5 | 26.8 | 18.8 |
| 2181 | 142.0 | 217.9 | 886.1 | 328.7 | 14.1 | 25.1 | 24.6 | 9.9 |
| 2679 | 141.1 | 130.5 | 481.5 | 246.5 | 18.6 | 26.8 | 21.4 | 12.5 |
| 2550 | 118.2 | 118.8 | 375.7 | 174.2 | 7.7 | 20.7 | 19.8 | 10.6 |
| 2278 | 305.0 | 360.8 | 1086.7 | 916.8 | 46.9 | 47.4 | 40.6 | 47.0 |
| 2756 | 163.4 | 213.2 | 499.0 | 319.8 | 5.3 | 26.2 | 20.7 | 11.0 |
| 3487 | 109.9 | 116.7 | 255.4 | 169.0 | 7.2 | 17.9 | 22.5 | 10.9 |
| 2688 | 837.6 | 877.9 | 1636.4 | 2258.5 | 38.4 | 41.3 | 30.2 | 30.7 |
| 2227 | 162.7 | 231.4 | 655.3 | 461.5 | 6.1 | 32.1 | 29.1 | 19.2 |
| 2525 | 174.1 | 225.6 | 545.6 | 404.3 | 10.6 | 28.5 | 21.2 | 11.2 |
| 2771 | 304.2 | 412.0 | 762.1 | 756.2 | 17.9 | 63.3 | 23.7 | 22.0 |
| 2752 | 91.5 | 153.6 | 312.4 | 233.3 | 3.2 | 29.8 | 19.9 | 9.9 |
| 2695 | 177.6 | 173.3 | 477.1 | 306.0 | 13.6 | 44.4 | 25.2 | 21.0 |
| 2263 | 118.0 | 189.4 | 347.4 | 260.6 | 3.6 | 30.7 | 19.9 | 10.2 |
| 2273 | 117.9 | 150.1 | 427.9 | 230.7 | 17.6 | 37.9 | 26.7 | 17.2 |
| 3488 | 123.7 | 148.8 | 380.0 | 222.1 | 9.8 | 20.6 | 20.7 | 10.5 |
| 2661 | 131.5 | 141.4 | 545.1 | 212.0 | 8.0 | 34.6 | 18.8 | 10.0 |
| 2201 | 193.5 | 216.6 | 670.4 | 401.8 | 36.7 | 55.1 | 37.3 | 29.9 |
| 2261 | 241.5 | 326.3 | 899.6 | 664.1 | 22.6 | 57.9 | 33.3 | 25.7 |
| 2203 | 181.7 | 249.3 | 677.4 | 461.3 | 2.8 | 30.8 | 19.4 | 11.6 |
| 2193 | 190.3 | 234.9 | 615.2 | 378.3 | 22.0 | 51.3 | 25.4 | 26.3 |
| 3328 | 112.0 | 126.8 | 370.3 | 201.2 | 9.7 | 23.9 | 25.1 | 15.5 |
| 3484 | 90.2 | 166.9 | 319.8 | 257.9 | 7.3 | 31.8 | 21.8 | 10.3 |
| 2202 | 113.8 | 122.5 | 226.0 | 185.5 | 3.7 | 38.2 | 22.1 | 11.4 |
| 2236 | 164.6 | 239.7 | 660.7 | 409.3 | 16.2 | 39.5 | 23.5 | 14.3 |
| 3097 | 109.3 | 176.9 | 245.0 | 313.2 | 2.1 | 26.4 | 18.4 | 8.9 |
| 2284 | 340.7 | 339.4 | 973.2 | 781.6 | 34.7 | 56.7 | 35.4 | 17.8 |
| 2253 | 210.1 | 287.6 | 617.1 | 538.1 | 15.0 | 35.2 | 23.7 | 14.9 |
| 2498 | 365.5 | 405.7 | 1081.8 | 986.3 | 40.3 | 39.6 | 25.4 | 13.5 |
| 2466 | 159.8 | 198.4 | 509.3 | 358.1 | 3.8 | 10.3 | 17.1 | 12.3 |
| 2206 | 308.4 | 361.6 | 1267.9 | 739.2 | 16.6 | 51.1 | 25.9 | 20.9 |
| 2327 | 172.2 | 205.1 | 703.3 | 340.5 | 12.0 | 43.4 | 24.1 | 13.0 |
| 2774 | 281.0 | 370.8 | 920.6 | 706.5 | 24.0 | 54.7 | 27.1 | 15.2 |
| 2210 | 130.7 | 184.1 | 695.3 | 263.5 | 2.2 | 7.8 | 18.5 | 11.5 |
| 2268 | 176.7 | 236.5 | 541.2 | 468.9 | 17.0 | 19.2 | 26.3 | 13.8 |
| 2239 | 171.9 | 226.8 | 814.9 | 320.3 | 11.7 | 23.6 | 23.8 | 10.5 |
| 2250 | 347.8 | 402.7 | 1299.3 | 947.0 | 27.1 | 39.6 | 31.7 | 25.7 |
| 2205 | 383.5 | 423.9 | 1670.5 | 967.3 | 30.9 | 61.4 | 29.4 | 30.2 |
| 2754 | 531.3 | 533.5 | 2003.1 | 1349.0 | 58.8 | 82.8 | 40.6 | 88.4 |
| 2665 | 458.4 | 520.8 | 1761.2 | 1082.2 | 18.0 | 38.4 | 27.4 | 17.2 |

## Supplementary Table 2H. Expression of *GMCSF*, probe set id 210229_s_at.

*n* = 277

| **Sample** | **PCR** | **MAS** | **dChip** | **RMA** | **GCRMA** |
| --- | --- | --- | --- | --- | --- |
| 322 | 0.0 | 2.1 | 64.2 | 69.5 | 7.0 |
| 2291 | 0.0 | 3.4 | 62.0 | 71.6 | 8.1 |
| 2285 | 0.0 | 2.0 | 53.2 | 66.6 | 7.6 |
| 2243 | 0.0 | 3.3 | 58.4 | 77.8 | 8.5 |
| 1551 | 0.0 | 4.6 | 49.2 | 61.6 | 8.0 |
| 1595 | 0.0 | 4.0 | 35.5 | 68.4 | 7.4 |
| 2301 | 0.0 | 5.2 | 43.7 | 76.7 | 8.6 |
| 3279 | 0.0 | 1.7 | 43.4 | 58.0 | 7.3 |
| 1747 | 0.0 | 2.6 | 76.1 | 61.5 | 7.2 |
| 2287 | 0.0 | 3.3 | 65.2 | 75.3 | 10.5 |
| 2326 | 0.0 | 1.7 | 63.4 | 62.1 | 7.4 |
| 1197 | 0.0 | 4.7 | 40.8 | 88.1 | 8.5 |
| 1482 | 0.0 | 3.8 | 81.6 | 73.0 | 11.3 |
| 2511 | 0.0 | 5.9 | 58.8 | 63.0 | 7.9 |
| 2669 | 0.0 | 2.6 | 79.3 | 70.8 | 8.2 |
| 2305 | 0.0 | 2.5 | 54.4 | 71.5 | 8.3 |
| 3331 | 0.0 | 3.0 | 45.4 | 64.0 | 7.7 |
| 2216 | 0.0 | 3.9 | 40.7 | 69.8 | 8.0 |
| 2248 | 0.0 | 2.2 | 59.1 | 66.1 | 8.0 |
| 2220 | 0.0 | 11.4 | 43.2 | 65.7 | 7.9 |
| 2752 | 0.0 | 2.7 | 46.0 | 68.0 | 7.4 |
| 2170 | 0.0 | 3.5 | 70.3 | 69.6 | 8.1 |
| 2543 | 0.0 | 3.1 | 46.3 | 58.9 | 7.9 |
| 2171 | 0.0 | 2.7 | 54.0 | 70.5 | 8.0 |
| 2247 | 0.0 | 2.0 | 45.1 | 65.5 | 7.6 |
| 2299 | 0.0 | 3.9 | 52.4 | 80.8 | 11.1 |
| 1063 | 0.0 | 3.2 | 78.3 | 83.9 | 9.1 |
| 3289 | 0.0 | 4.5 | 72.6 | 82.3 | 8.2 |
| 2176 | 0.0 | 7.3 | 63.4 | 70.1 | 7.8 |
| 2240 | 0.0 | 2.5 | 45.8 | 75.5 | 7.8 |
| 2198 | 0.0 | 4.2 | 70.0 | 75.6 | 9.5 |
| 1448 | 0.0 | 3.8 | 50.3 | 74.4 | 12.0 |
| 2252 | 0.0 | 5.0 | 61.5 | 68.4 | 9.6 |
| 2177 | 0.0 | 2.0 | 54.6 | 61.4 | 6.9 |
| 2644 | 0.0 | 28.4 | 74.1 | 62.6 | 8.4 |
| 3482 | 0.0 | 2.7 | 58.6 | 74.2 | 8.2 |
| 2766 | 0.0 | 3.6 | 34.1 | 79.8 | 9.4 |
| 2300 | 0.0 | 2.9 | 78.7 | 82.9 | 8.2 |
| 2751 | 0.0 | 4.2 | 102.4 | 70.4 | 8.2 |
| 3317 | 0.0 | 2.5 | 35.7 | 68.5 | 7.9 |
| 2290 | 0.0 | 4.7 | 38.5 | 68.6 | 8.1 |
| 1766 | 0.0 | 3.4 | 65.8 | 70.0 | 8.0 |
| 2185 | 0.0 | 2.2 | 73.2 | 65.5 | 7.8 |
| 2307 | 0.0 | 4.5 | 63.9 | 92.4 | 8.5 |
| 2212 | 0.0 | 2.5 | 33.9 | 70.9 | 7.9 |
| 3285 | 0.0 | 2.9 | 81.3 | 73.4 | 8.2 |
| 3315 | 0.0 | 20.0 | 65.1 | 103.7 | 17.2 |
| 2468 | 0.0 | 3.6 | 59.6 | 73.2 | 8.0 |
| 2184 | 0.0 | 7.0 | 70.1 | 73.2 | 10.4 |
| 2199 | 0.0 | 3.1 | 55.4 | 76.4 | 8.2 |
| 3115 | 0.0 | 4.1 | 97.2 | 77.1 | 8.8 |
| 2197 | 0.0 | 2.3 | 69.1 | 66.8 | 8.1 |
| 2181 | 0.0 | 2.6 | 45.4 | 59.6 | 7.3 |
| 3333 | 0.0 | 3.4 | 62.0 | 66.5 | 8.3 |
| 2670 | 0.0 | 2.4 | 60.7 | 61.8 | 7.4 |
| 2771 | 0.0 | 1.0 | 67.1 | 67.3 | 7.9 |
| 2681 | 0.0 | 8.9 | 52.0 | 97.9 | 8.6 |
| 2214 | 0.0 | 2.4 | 79.1 | 66.9 | 7.9 |
| 2480 | 0.0 | 4.0 | 47.0 | 93.5 | 10.2 |
| 3326 | 0.0 | 2.2 | 71.8 | 62.9 | 7.4 |
| 2306 | 0.0 | 5.1 | 54.4 | 87.5 | 9.4 |
| 2182 | 0.0 | 3.2 | 59.5 | 66.6 | 7.5 |
| 2187 | 0.0 | 2.8 | 55.8 | 68.7 | 7.5 |
| 2193 | 0.0 | 2.6 | 56.7 | 75.7 | 8.2 |
| 2218 | 0.0 | 2.0 | 54.4 | 57.8 | 7.2 |
| 2281 | 0.0 | 2.9 | 53.3 | 68.1 | 7.4 |
| 2749 | 0.0 | 3.0 | 52.4 | 70.0 | 7.8 |
| 2284 | 0.0 | 3.7 | 54.1 | 68.3 | 7.7 |
| 1188 | 0.0 | 4.2 | 52.1 | 66.5 | 8.0 |
| 1316 | 0.0 | 3.4 | 41.4 | 68.0 | 8.2 |
| 2242 | 0.1 | 4.7 | 48.1 | 80.3 | 8.1 |
| 2278 | 0.1 | 2.8 | 63.9 | 77.9 | 8.2 |
| 2496 | 0.1 | 5.0 | 75.4 | 88.7 | 9.4 |
| 2208 | 0.1 | 3.3 | 58.9 | 77.7 | 8.1 |
| 2546 | 0.1 | 3.4 | 89.0 | 67.8 | 8.1 |
| 2764 | 0.1 | 3.5 | 92.0 | 83.6 | 8.5 |
| 2748 | 0.1 | 3.1 | 50.2 | 70.0 | 8.0 |
| 1299 | 0.1 | 2.2 | 26.1 | 65.8 | 7.6 |
| 2238 | 0.1 | 3.3 | 90.9 | 53.3 | 6.9 |
| 2246 | 0.1 | 2.0 | 49.0 | 66.6 | 6.7 |
| 2510 | 0.1 | 3.3 | 52.7 | 73.4 | 8.1 |
| 3097 | 0.1 | 4.1 | 61.5 | 71.0 | 8.0 |
| 2172 | 0.1 | 1.9 | 53.7 | 69.7 | 7.9 |
| 2262 | 0.1 | 3.2 | 55.9 | 70.0 | 8.1 |
| 2687 | 0.1 | 5.4 | 78.8 | 80.0 | 8.3 |
| 2245 | 0.1 | 3.1 | 79.7 | 76.5 | 8.0 |
| 1401 | 0.1 | 3.6 | 42.5 | 74.3 | 7.7 |
| 2270 | 0.1 | 4.8 | 83.6 | 69.8 | 8.3 |
| 2192 | 0.1 | 3.2 | 42.3 | 68.2 | 8.1 |
| 2174 | 0.1 | 5.1 | 46.9 | 75.2 | 8.7 |
| 3277 | 0.1 | 3.1 | 59.3 | 62.3 | 7.6 |
| 2665 | 0.1 | 2.9 | 75.2 | 58.1 | 7.9 |
| 2690 | 0.1 | 16.5 | 92.3 | 85.6 | 8.6 |
| 2774 | 0.1 | 3.5 | 56.9 | 69.6 | 7.6 |
| 2173 | 0.1 | 6.6 | 71.9 | 77.5 | 8.3 |
| 2524 | 0.1 | 3.2 | 52.5 | 78.3 | 8.3 |
| 2550 | 0.1 | 3.5 | 69.4 | 70.2 | 8.0 |
| 2656 | 0.1 | 3.3 | 77.7 | 66.4 | 8.0 |
| 2188 | 0.1 | 1.9 | 52.9 | 62.5 | 6.3 |
| 3486 | 0.1 | 4.8 | 75.1 | 97.4 | 9.9 |
| 2200 | 0.1 | 4.2 | 46.7 | 76.9 | 7.8 |
| 2179 | 0.1 | 2.5 | 75.1 | 71.0 | 8.0 |
| 2237 | 0.1 | 2.0 | 42.2 | 61.7 | 6.7 |
| 3309 | 0.1 | 2.9 | 48.0 | 58.3 | 6.9 |
| 2516 | 0.1 | 3.2 | 98.0 | 69.6 | 8.3 |
| 2183 | 0.1 | 2.4 | 53.9 | 56.8 | 7.6 |
| 2466 | 0.1 | 3.3 | 76.7 | 73.8 | 9.3 |
| 2169 | 0.1 | 18.6 | 49.9 | 68.4 | 8.3 |
| 2241 | 0.1 | 3.1 | 55.0 | 62.7 | 7.8 |
| 2194 | 0.1 | 4.3 | 49.3 | 70.8 | 8.0 |
| 2302 | 0.1 | 5.4 | 76.1 | 74.1 | 8.2 |
| 2229 | 0.1 | 3.2 | 67.3 | 69.4 | 8.9 |
| 2239 | 0.1 | 2.4 | 48.1 | 58.1 | 7.6 |
| 2231 | 0.1 | 4.0 | 43.5 | 76.9 | 7.6 |
| 3490 | 0.1 | 6.8 | 91.7 | 76.7 | 8.9 |
| 3098 | 0.1 | 2.9 | 84.3 | 72.6 | 8.6 |
| 2757 | 0.1 | 3.6 | 53.3 | 85.1 | 8.3 |
| 2289 | 0.1 | 2.9 | 64.9 | 68.6 | 8.0 |
| 3311 | 0.1 | 2.7 | 56.4 | 68.8 | 8.0 |
| 2175 | 0.1 | 2.9 | 50.6 | 70.0 | 7.4 |
| 2204 | 0.1 | 3.3 | 71.9 | 70.5 | 8.2 |
| 3099 | 0.1 | 6.0 | 49.6 | 74.5 | 8.4 |
| 2647 | 0.1 | 4.5 | 67.7 | 70.3 | 8.2 |
| 2186 | 0.1 | 2.9 | 67.1 | 67.1 | 7.9 |
| 2694 | 0.1 | 4.8 | 64.0 | 65.4 | 8.2 |
| 2767 | 0.1 | 2.2 | 59.1 | 72.0 | 8.5 |
| 2688 | 0.2 | 8.2 | 80.6 | 89.3 | 9.9 |
| 3319 | 0.2 | 4.3 | 66.8 | 83.8 | 9.7 |
| 2271 | 0.2 | 10.3 | 63.2 | 78.6 | 9.5 |
| 3314 | 0.2 | 3.0 | 49.1 | 62.1 | 7.9 |
| 3493 | 0.2 | 3.7 | 63.0 | 86.1 | 11.2 |
| 3321 | 0.2 | 3.3 | 60.5 | 66.4 | 7.4 |
| 3322 | 0.2 | 2.8 | 69.0 | 72.9 | 8.1 |
| 2509 | 0.2 | 3.4 | 77.3 | 76.0 | 10.5 |
| 2206 | 0.2 | 3.1 | 76.1 | 74.2 | 8.2 |
| 3492 | 0.2 | 3.1 | 50.1 | 68.4 | 8.2 |
| 2224 | 0.2 | 4.5 | 30.9 | 68.0 | 8.1 |
| 2286 | 0.2 | 3.6 | 61.3 | 74.0 | 8.3 |
| 2753 | 0.2 | 3.2 | 44.9 | 61.1 | 7.7 |
| 2225 | 0.2 | 1.4 | 56.6 | 54.1 | 6.3 |
| 1174 | 0.2 | 5.4 | 46.7 | 75.5 | 8.1 |
| 2201 | 0.2 | 4.5 | 61.2 | 82.7 | 8.1 |
| 2750 | 0.2 | 3.4 | 69.9 | 72.9 | 8.2 |
| 3324 | 0.2 | 2.5 | 59.1 | 61.3 | 7.0 |
| 2772 | 0.2 | 6.1 | 81.6 | 86.0 | 10.2 |
| 3316 | 0.2 | 1.7 | 52.7 | 65.9 | 7.8 |
| 3312 | 0.2 | 3.5 | 43.6 | 67.7 | 7.9 |
| 2680 | 0.2 | 2.5 | 67.2 | 62.7 | 7.8 |
| 2671 | 0.2 | 2.5 | 62.3 | 71.2 | 8.3 |
| 2541 | 0.2 | 4.2 | 62.5 | 75.7 | 8.2 |
| 2773 | 0.2 | 4.4 | 43.1 | 73.6 | 8.1 |
| 3327 | 0.2 | 3.1 | 61.2 | 68.0 | 8.3 |
| 1432 | 0.2 | 2.1 | 56.2 | 67.3 | 8.0 |
| 2692 | 0.2 | 3.1 | 15.1 | 69.8 | 8.4 |
| 2514 | 0.2 | 6.6 | 76.1 | 100.1 | 12.3 |
| 3329 | 0.2 | 3.4 | 81.2 | 72.3 | 8.2 |
| 2250 | 0.2 | 1.9 | 79.3 | 57.0 | 7.3 |
| 3293 | 0.2 | 5.5 | 70.5 | 76.7 | 10.1 |
| 2265 | 0.2 | 2.0 | 70.3 | 69.1 | 8.0 |
| 2189 | 0.2 | 1.7 | 48.2 | 57.1 | 7.6 |
| 2233 | 0.2 | 2.1 | 51.9 | 62.5 | 6.4 |
| 3308 | 0.2 | 7.8 | 59.2 | 62.2 | 7.9 |
| 3330 | 0.2 | 3.4 | 61.6 | 67.7 | 7.8 |
| 3096 | 0.3 | 2.2 | 67.1 | 62.4 | 7.9 |
| 2704 | 0.3 | 5.6 | 62.0 | 98.6 | 9.0 |
| 2549 | 0.3 | 3.9 | 66.3 | 69.6 | 7.9 |
| 1201 | 0.3 | 2.5 | 20.8 | 59.3 | 6.9 |
| 3484 | 0.3 | 3.1 | 73.4 | 69.6 | 7.6 |
| 2539 | 0.3 | 4.1 | 57.1 | 66.0 | 8.1 |
| 2272 | 0.3 | 3.2 | 66.4 | 67.8 | 10.9 |
| 2178 | 0.3 | 3.3 | 39.6 | 72.0 | 7.6 |
| 2254 | 0.3 | 3.1 | 52.6 | 73.3 | 8.8 |
| 2525 | 0.3 | 4.2 | 53.7 | 77.9 | 8.3 |
| 2683 | 0.3 | 7.8 | 93.3 | 83.7 | 9.4 |
| 2292 | 0.3 | 2.4 | 92.0 | 73.8 | 8.7 |
| 2282 | 0.3 | 3.4 | 54.9 | 79.3 | 8.1 |
| 3488 | 0.3 | 7.4 | 95.8 | 67.4 | 7.4 |
| 2230 | 0.4 | 5.5 | 62.2 | 72.4 | 8.2 |
| 3318 | 0.4 | 4.4 | 64.5 | 78.1 | 7.6 |
| 2765 | 0.4 | 4.3 | 76.6 | 80.8 | 8.0 |
| 2497 | 0.4 | 4.0 | 84.2 | 77.6 | 10.2 |
| 3286 | 0.4 | 1.9 | 81.1 | 65.6 | 7.9 |
| 2275 | 0.4 | 3.0 | 89.7 | 73.3 | 8.1 |
| 2227 | 0.4 | 6.0 | 70.8 | 85.5 | 8.9 |
| 3323 | 0.4 | 3.2 | 90.6 | 66.2 | 8.2 |
| 2551 | 0.4 | 6.9 | 59.9 | 83.7 | 8.5 |
| 2536 | 0.4 | 5.5 | 47.7 | 84.3 | 8.6 |
| 2682 | 0.4 | 5.8 | 76.0 | 71.0 | 8.2 |
| 2769 | 0.4 | 5.6 | 73.7 | 83.8 | 10.1 |
| 2754 | 0.4 | 4.2 | 77.5 | 73.1 | 8.3 |
| 2304 | 0.4 | 5.6 | 65.3 | 83.6 | 9.4 |
| 2507 | 0.4 | 4.5 | 61.2 | 72.0 | 8.1 |
| 3483 | 0.4 | 4.6 | 41.4 | 74.7 | 8.3 |
| 2273 | 0.5 | 3.4 | 61.0 | 74.8 | 8.1 |
| 2534 | 0.5 | 5.2 | 56.4 | 83.0 | 9.7 |
| 2696 | 0.5 | 9.9 | 91.2 | 73.8 | 9.8 |
| 2219 | 0.5 | 2.6 | 53.0 | 60.0 | 8.0 |
| 3320 | 0.5 | 5.3 | 67.6 | 65.8 | 8.1 |
| 2249 | 0.5 | 4.3 | 52.0 | 73.5 | 8.0 |
| 2686 | 0.5 | 5.9 | 80.8 | 80.9 | 8.9 |
| 3304 | 0.6 | 10.8 | 46.5 | 78.0 | 8.3 |
| 2467 | 0.6 | 7.1 | 85.6 | 94.5 | 16.9 |
| 3328 | 0.6 | 4.6 | 73.6 | 83.0 | 10.0 |
| 2236 | 0.6 | 2.0 | 48.9 | 66.0 | 8.0 |
| 2256 | 0.6 | 4.1 | 68.6 | 75.8 | 9.4 |
| 2658 | 0.6 | 2.5 | 64.5 | 63.0 | 8.0 |
| 2207 | 0.6 | 3.5 | 61.4 | 65.1 | 8.1 |
| 3295 | 0.6 | 2.5 | 66.4 | 76.8 | 8.3 |
| 3334 | 0.7 | 3.1 | 27.5 | 63.0 | 6.9 |
| 2235 | 0.7 | 2.4 | 68.7 | 74.9 | 8.4 |
| 2191 | 0.7 | 2.4 | 58.2 | 70.4 | 7.5 |
| 2195 | 0.7 | 3.2 | 59.9 | 65.9 | 7.7 |
| 3491 | 0.7 | 2.3 | 40.4 | 63.3 | 7.0 |
| 3278 | 0.7 | 5.4 | 63.2 | 73.9 | 9.2 |
| 2203 | 0.8 | 3.3 | 65.6 | 63.3 | 7.8 |
| 2263 | 0.8 | 2.1 | 59.9 | 62.8 | 7.7 |
| 2535 | 0.8 | 17.5 | 41.8 | 76.9 | 8.4 |
| 3481 | 0.8 | 5.0 | 59.7 | 88.6 | 11.3 |
| 2196 | 0.8 | 2.0 | 66.0 | 65.2 | 7.7 |
| 3485 | 0.8 | 8.0 | 93.3 | 105.1 | 8.6 |
| 2689 | 0.8 | 5.1 | 58.9 | 66.5 | 8.3 |
| 2261 | 0.8 | 2.7 | 54.8 | 62.3 | 7.2 |
| 2668 | 0.9 | 3.6 | 49.4 | 63.7 | 7.5 |
| 2498 | 0.9 | 5.9 | 29.2 | 73.4 | 8.4 |
| 3101 | 0.9 | 2.6 | 42.5 | 71.1 | 7.3 |
| 2679 | 1.0 | 2.9 | 59.2 | 67.6 | 8.2 |
| 3313 | 1.0 | 4.4 | 74.6 | 90.2 | 8.9 |
| 2545 | 1.0 | 14.2 | 68.0 | 73.8 | 9.3 |
| 2542 | 1.0 | 4.9 | 54.7 | 62.6 | 7.8 |
| 3310 | 1.0 | 15.8 | 57.3 | 77.1 | 15.9 |
| 2695 | 1.1 | 3.8 | 79.5 | 61.9 | 8.2 |
| 3489 | 1.1 | 2.7 | 45.8 | 62.9 | 7.5 |
| 3292 | 1.1 | 5.2 | 61.3 | 99.2 | 9.4 |
| 3332 | 1.1 | 14.0 | 67.4 | 66.9 | 8.5 |
| 2762 | 1.2 | 4.9 | 39.0 | 84.2 | 8.5 |
| 2228 | 1.2 | 3.9 | 68.8 | 68.0 | 10.7 |
| 2222 | 1.2 | 3.6 | 46.9 | 83.4 | 8.3 |
| 3325 | 1.4 | 3.2 | 62.8 | 62.0 | 8.0 |
| 2490 | 1.4 | 2.6 | 64.8 | 61.3 | 7.7 |
| 2251 | 1.4 | 7.2 | 93.9 | 94.2 | 18.0 |
| 2226 | 1.6 | 2.0 | 51.2 | 63.3 | 7.5 |
| 2234 | 1.6 | 2.9 | 31.6 | 78.5 | 8.8 |
| 2190 | 1.7 | 5.1 | 85.3 | 86.4 | 11.4 |
| 2268 | 1.7 | 5.3 | 76.5 | 78.9 | 13.8 |
| 3301 | 2.1 | 4.4 | 73.3 | 68.6 | 8.0 |
| 2756 | 2.2 | 2.4 | 75.0 | 64.3 | 8.1 |
| 2664 | 2.3 | 4.6 | 41.0 | 78.3 | 8.3 |
| 2327 | 2.4 | 2.6 | 94.4 | 63.0 | 7.8 |
| 2244 | 2.5 | 4.2 | 83.1 | 86.4 | 16.8 |
| 2257 | 2.9 | 3.0 | 16.3 | 81.3 | 8.7 |
| 2267 | 2.9 | 3.7 | 49.4 | 78.0 | 8.9 |
| 2296 | 3.0 | 4.0 | 89.4 | 72.1 | 8.8 |
| 2667 | 3.0 | 3.5 | 62.8 | 77.6 | 8.2 |
| 2217 | 3.2 | 1.9 | 66.0 | 73.8 | 7.6 |
| 2205 | 3.2 | 4.2 | 39.3 | 67.4 | 8.0 |
| 2260 | 5.4 | 5.7 | 69.0 | 75.3 | 9.1 |
| 3102 | 5.6 | 2.5 | 69.3 | 70.8 | 8.0 |
| 2666 | 6.0 | 2.4 | 61.3 | 58.5 | 7.8 |
| 2661 | 6.2 | 15.2 | 73.9 | 66.8 | 7.4 |
| 2655 | 6.3 | 2.6 | 75.8 | 71.8 | 8.0 |
| 3487 | 6.3 | 2.2 | 62.1 | 67.4 | 7.7 |
| 2288 | 7.7 | 3.2 | 79.4 | 72.9 | 8.3 |
| 2276 | 8.2 | 3.0 | 79.7 | 68.5 | 8.2 |
| 2327 | 8.7 | 8.9 | 39.7 | 85.0 | 12.8 |
| 2672 | 9.1 | 3.9 | 52.6 | 74.1 | 8.1 |
| 2685 | 9.2 | 5.4 | 78.9 | 64.0 | 8.2 |
| 2544 | 10.7 | 3.3 | 42.5 | 76.8 | 8.4 |
| 2209 | 11.4 | 5.5 | 62.1 | 76.7 | 8.1 |
| 2259 | 14.5 | 2.0 | 110.1 | 69.9 | 8.0 |
| 2202 | 18.6 | 3.2 | 35.8 | 72.9 | 8.4 |
| 3100 | 24.5 | 3.3 | 67.4 | 66.4 | 8.8 |
| 2274 | 30.3 | 4.4 | 84.6 | 87.1 | 10.2 |
| 2210 | 30.8 | 4.6 | 67.7 | 74.1 | 8.3 |
| 2678 | 31.3 | 2.3 | 71.5 | 71.6 | 8.0 |
| 2747 | 54.1 | 3.8 | 53.8 | 67.1 | 7.4 |
| 2676 | 153.5 | 16.4 | 96.4 | 78.6 | 16.1 |
| 2253 | 568.1 | 58.4 | 122.3 | 98.5 | 31.6 |

## Supplementary Table 2I. Expression of *TRKA*, probe set id 208605_s_at.

*n* = 276

| **Sample** | **PCR** | **MAS** | **dChip** | **RMA** | **GCRMA** |
| --- | --- | --- | --- | --- | --- |
| 2467 | 0.0 | 1.9 | -13.8 | 31.1 | 5.0 |
| 1482 | 0.0 | 2.3 | 7.9 | 34.3 | 5.0 |
| 2514 | 0.0 | 1.8 | 1.9 | 35.5 | 5.5 |
| 2220 | 0.0 | 0.8 | 2.7 | 25.2 | 4.8 |
| 2269 | 0.0 | 38.2 | 31.6 | 42.9 | 9.1 |
| 3316 | 0.0 | 1.5 | -6.6 | 34.2 | 5.0 |
| 2291 | 0.0 | 3.9 | -2.8 | 25.2 | 4.9 |
| 2184 | 0.0 | 0.9 | -0.9 | 27.8 | 4.9 |
| 2217 | 0.0 | 0.8 | 4.0 | 28.9 | 4.4 |
| 2260 | 0.0 | 0.8 | -2.7 | 27.9 | 4.9 |
| 2285 | 0.0 | 3.2 | -3.9 | 26.8 | 4.8 |
| 2749 | 0.0 | 1.2 | 1.1 | 28.6 | 5.0 |
| 3278 | 0.0 | 3.0 | 1.0 | 30.5 | 5.1 |
| 2539 | 0.0 | 1.6 | -9.6 | 28.6 | 5.0 |
| 3096 | 0.0 | 2.2 | 0.6 | 29.3 | 4.9 |
| 3322 | 0.0 | 2.6 | -6.4 | 32.4 | 5.0 |
| 2244 | 0.0 | 1.1 | -3.2 | 33.6 | 5.1 |
| 2301 | 0.0 | 1.5 | -10.2 | 24.1 | 4.6 |
| 2171 | 0.0 | 0.6 | -7.8 | 24.3 | 4.7 |
| 2304 | 0.0 | 11.5 | 1.2 | 41.0 | 5.7 |
| 2175 | 0.0 | 2.4 | 2.0 | 25.2 | 4.7 |
| 2534 | 0.0 | 1.3 | -3.9 | 27.6 | 5.2 |
| 2305 | 0.0 | 1.5 | 2.7 | 31.9 | 5.3 |
| 2300 | 0.0 | 1.9 | -7.3 | 32.0 | 5.1 |
| 2771 | 0.0 | 4.1 | -2.2 | 29.6 | 4.9 |
| 1197 | 0.0 | 5.0 | -2.9 | 40.7 | 5.0 |
| 2185 | 0.0 | 1.5 | -4.9 | 26.3 | 4.7 |
| 3325 | 0.0 | 0.8 | 2.2 | 26.3 | 5.0 |
| 2694 | 0.0 | 1.4 | -16.7 | 30.3 | 5.3 |
| 2231 | 0.0 | 1.3 | -6.0 | 27.0 | 4.7 |
| 2671 | 0.0 | 1.3 | -4.4 | 25.5 | 5.0 |
| 1432 | 0.0 | 0.6 | 0.2 | 26.6 | 4.9 |
| 2682 | 0.0 | 4.5 | -2.2 | 29.0 | 5.2 |
| 2766 | 0.0 | 6.0 | 11.7 | 31.1 | 5.0 |
| 2655 | 0.0 | 3.5 | 0.5 | 32.9 | 4.8 |
| 2670 | 0.0 | 1.3 | -1.3 | 26.7 | 4.4 |
| 2227 | 0.0 | 1.9 | -0.9 | 29.5 | 5.5 |
| 3289 | 0.0 | 1.3 | -17.4 | 23.9 | 5.0 |
| 2289 | 0.0 | 0.8 | 0.2 | 23.9 | 5.0 |
| 2216 | 0.0 | 0.9 | 7.1 | 24.5 | 5.0 |
| 2272 | 0.0 | 1.6 | -2.0 | 25.8 | 4.9 |
| 2535 | 0.0 | 1.2 | -14.1 | 27.9 | 5.0 |
| 2497 | 0.0 | 1.6 | 3.5 | 35.1 | 5.4 |
| 1188 | 0.0 | 0.9 | 5.1 | 31.0 | 5.0 |
| 2550 | 0.0 | 2.3 | -8.3 | 22.4 | 4.8 |
| 3279 | 0.0 | 2.1 | 2.2 | 25.9 | 4.3 |
| 2689 | 0.0 | 12.4 | 7.5 | 27.4 | 5.2 |
| 2545 | 0.0 | 1.2 | -13.0 | 27.2 | 5.1 |
| 2224 | 0.0 | 0.9 | -2.6 | 31.8 | 4.8 |
| 3097 | 0.0 | 5.2 | -0.7 | 34.6 | 4.9 |
| 2490 | 0.0 | 1.1 | 10.1 | 36.0 | 5.5 |
| 3317 | 0.1 | 0.8 | -1.6 | 26.1 | 5.0 |
| 2692 | 0.1 | 2.3 | -9.5 | 31.9 | 5.4 |
| 2198 | 0.1 | 2.6 | 2.3 | 31.4 | 5.0 |
| 1174 | 0.1 | 2.2 | -5.3 | 26.3 | 4.9 |
| 2278 | 0.1 | 3.7 | 1.1 | 29.3 | 5.1 |
| 2176 | 0.1 | 1.3 | 0.6 | 24.1 | 4.5 |
| 2541 | 0.1 | 0.9 | -2.5 | 27.6 | 5.1 |
| 2767 | 0.1 | 2.1 | -2.2 | 23.0 | 5.1 |
| 1595 | 0.1 | 2.0 | 4.6 | 25.8 | 4.8 |
| 3484 | 0.1 | 1.4 | -2.9 | 26.9 | 4.8 |
| 2690 | 0.1 | 1.1 | -9.2 | 28.7 | 5.5 |
| 2246 | 0.1 | 6.4 | 6.8 | 32.9 | 4.7 |
| 3488 | 0.1 | 1.7 | -4.6 | 27.1 | 4.9 |
| 2299 | 0.1 | 3.6 | 1.5 | 24.3 | 5.1 |
| 2199 | 0.1 | 1.0 | -6.6 | 27.2 | 5.2 |
| 2186 | 0.1 | 2.5 | -6.1 | 26.3 | 4.8 |
| 2222 | 0.1 | 1.9 | 17.1 | 32.2 | 5.6 |
| 2765 | 0.1 | 0.8 | -0.5 | 28.9 | 5.0 |
| 2247 | 0.1 | 2.2 | 0.9 | 27.0 | 4.7 |
| 2525 | 0.1 | 4.1 | -3.2 | 32.8 | 5.2 |
| 2282 | 0.1 | 1.1 | -10.3 | 27.7 | 4.9 |
| 1201 | 0.1 | 0.9 | -3.3 | 29.4 | 4.7 |
| 2687 | 0.1 | 2.0 | -17.0 | 25.8 | 5.4 |
| 2290 | 0.1 | 3.5 | 3.6 | 28.0 | 4.9 |
| 2769 | 0.1 | 4.5 | -12.9 | 30.1 | 5.4 |
| 2209 | 0.1 | 2.6 | -0.5 | 29.6 | 4.9 |
| 3490 | 0.1 | 1.2 | -7.9 | 27.0 | 5.4 |
| 2256 | 0.1 | 2.2 | 3.3 | 37.0 | 5.0 |
| 2265 | 0.1 | 1.9 | -10.8 | 25.4 | 5.0 |
| 2194 | 0.1 | 3.9 | -1.0 | 30.2 | 4.8 |
| 1766 | 0.1 | 1.2 | -3.1 | 26.6 | 5.0 |
| 2543 | 0.1 | 0.9 | 4.1 | 30.1 | 5.0 |
| 3493 | 0.1 | 1.9 | -1.5 | 26.3 | 5.1 |
| 2679 | 0.1 | 3.1 | -2.3 | 33.1 | 5.1 |
| 2191 | 0.1 | 3.2 | -0.3 | 29.5 | 4.8 |
| 3491 | 0.1 | 1.0 | -2.7 | 27.5 | 4.8 |
| 3310 | 0.1 | 7.3 | -2.7 | 82.6 | 5.9 |
| 2207 | 0.1 | 1.7 | -2.2 | 42.2 | 5.0 |
| 2507 | 0.1 | 1.2 | 1.7 | 28.6 | 5.0 |
| 2666 | 0.1 | 4.1 | -4.1 | 29.4 | 4.3 |
| 2669 | 0.1 | 5.1 | 3.0 | 28.7 | 5.1 |
| 2704 | 0.2 | 4.1 | -9.1 | 30.3 | 5.5 |
| 2248 | 0.2 | 5.1 | 6.5 | 26.8 | 5.2 |
| 2551 | 0.2 | 3.5 | -10.9 | 24.9 | 5.4 |
| 2510 | 0.2 | 1.4 | 3.1 | 32.9 | 5.0 |
| 2228 | 0.2 | 1.1 | -4.4 | 29.5 | 4.8 |
| 3333 | 0.2 | 3.1 | 0.2 | 30.5 | 5.2 |
| 2296 | 0.2 | 0.6 | -0.5 | 26.6 | 5.0 |
| 2658 | 0.2 | 0.9 | 4.8 | 28.1 | 4.8 |
| 2252 | 0.2 | 3.6 | 8.6 | 33.7 | 5.1 |
| 2773 | 0.2 | 3.0 | -5.3 | 29.9 | 5.1 |
| 2241 | 0.2 | 0.8 | 2.7 | 27.0 | 4.4 |
| 2644 | 0.2 | 4.5 | 8.0 | 32.8 | 5.4 |
| 2762 | 0.2 | 4.8 | 0.5 | 27.8 | 5.4 |
| 2680 | 0.2 | 8.4 | 9.6 | 36.9 | 5.1 |
| 2270 | 0.2 | 2.3 | 7.6 | 34.9 | 5.2 |
| 1063 | 0.2 | 1.9 | -1.4 | 41.5 | 5.2 |
| 1401 | 0.2 | 1.8 | -5.9 | 25.9 | 4.6 |
| 3323 | 0.2 | 3.5 | -3.1 | 30.4 | 5.1 |
| 2536 | 0.2 | 4.5 | -5.1 | 35.8 | 5.6 |
| 322 | 0.2 | 2.3 | 7.6 | 30.3 | 4.8 |
| 2756 | 0.2 | 1.1 | 1.4 | 29.2 | 5.0 |
| 2212 | 0.3 | 1.3 | 4.8 | 32.1 | 5.0 |
| 3321 | 0.3 | 1.0 | -4.0 | 27.2 | 4.9 |
| 2302 | 0.3 | 2.0 | -10.0 | 25.4 | 5.1 |
| 2206 | 0.3 | 2.8 | -4.0 | 27.1 | 5.0 |
| 1747 | 0.3 | 1.5 | 5.5 | 25.9 | 4.2 |
| 2772 | 0.3 | 3.3 | -2.2 | 37.4 | 5.3 |
| 2251 | 0.3 | 3.2 | 10.1 | 39.1 | 6.8 |
| 2229 | 0.3 | 1.9 | 5.7 | 32.2 | 4.8 |
| 2181 | 0.3 | 0.9 | 10.2 | 32.2 | 4.6 |
| 3489 | 0.3 | 2.6 | 9.6 | 28.9 | 4.9 |
| 2174 | 0.3 | 2.8 | -1.8 | 25.1 | 5.1 |
| 2546 | 0.3 | 2.4 | 0.1 | 28.4 | 5.1 |
| 2238 | 0.3 | 2.4 | -2.3 | 23.3 | 4.8 |
| 2188 | 0.3 | 1.7 | 4.9 | 27.9 | 4.5 |
| 2688 | 0.3 | 3.9 | -11.9 | 33.7 | 5.9 |
| 3486 | 0.3 | 7.5 | -3.2 | 28.1 | 5.2 |
| 2195 | 0.3 | 9.4 | 9.1 | 34.3 | 4.9 |
| 2187 | 0.3 | 1.3 | 10.0 | 34.8 | 4.9 |
| 3334 | 0.3 | 3.1 | 0.6 | 27.6 | 4.7 |
| 2219 | 0.4 | 1.2 | -4.2 | 32.3 | 4.9 |
| 2656 | 0.4 | 3.0 | 20.0 | 34.3 | 4.8 |
| 3098 | 0.4 | 3.3 | -1.0 | 27.5 | 5.1 |
| 3331 | 0.4 | 7.0 | 22.9 | 34.4 | 5.1 |
| 2647 | 0.4 | 2.8 | 9.8 | 40.3 | 5.1 |
| 2240 | 0.4 | 2.7 | 16.8 | 29.5 | 5.0 |
| 2667 | 0.4 | 5.4 | 16.7 | 34.8 | 5.3 |
| 1551 | 0.4 | 2.1 | 9.2 | 25.6 | 4.8 |
| 2249 | 0.4 | 2.2 | 3.7 | 34.0 | 5.0 |
| 2275 | 0.4 | 2.6 | 2.9 | 29.0 | 5.0 |
| 2683 | 0.4 | 3.7 | -9.3 | 54.1 | 5.5 |
| 2262 | 0.4 | 2.0 | -0.7 | 34.9 | 5.0 |
| 2661 | 0.4 | 1.0 | -8.1 | 25.5 | 4.5 |
| 2243 | 0.4 | 1.6 | 3.9 | 35.1 | 5.0 |
| 2327 | 0.4 | 1.7 | -0.5 | 26.3 | 4.9 |
| 3482 | 0.4 | 1.8 | 5.1 | 33.9 | 5.0 |
| 1316 | 0.4 | 2.1 | 3.7 | 33.2 | 5.1 |
| 2271 | 0.4 | 8.7 | 14.1 | 37.4 | 4.9 |
| 2261 | 0.5 | 3.0 | 5.1 | 29.6 | 4.7 |
| 2214 | 0.5 | 8.3 | 16.6 | 36.6 | 4.8 |
| 2257 | 0.5 | 8.0 | 13.6 | 38.9 | 5.1 |
| 2170 | 0.5 | 1.8 | 1.2 | 32.1 | 5.1 |
| 3293 | 0.5 | 18.4 | 28.4 | 46.9 | 6.1 |
| 2287 | 0.5 | 4.4 | 22.4 | 33.4 | 4.9 |
| 3314 | 0.5 | 4.8 | 22.0 | 41.1 | 4.9 |
| 3315 | 0.5 | 8.5 | -5.1 | 35.1 | 5.8 |
| 2281 | 0.5 | 1.0 | 23.3 | 37.0 | 4.9 |
| 2681 | 0.5 | 16.3 | -4.6 | 62.0 | 6.1 |
| 2234 | 0.5 | 4.2 | 10.8 | 35.4 | 4.9 |
| 3312 | 0.5 | 5.6 | 12.6 | 33.1 | 5.2 |
| 2686 | 0.5 | 18.8 | 14.3 | 36.6 | 5.3 |
| 3330 | 0.5 | 2.9 | 6.9 | 29.3 | 5.0 |
| 3481 | 0.5 | 4.5 | 4.6 | 39.6 | 5.6 |
| 1299 | 0.5 | 0.8 | 3.1 | 26.8 | 5.0 |
| 3115 | 0.5 | 4.1 | -4.3 | 37.2 | 5.4 |
| 3318 | 0.5 | 13.3 | 11.2 | 33.5 | 4.9 |
| 3492 | 0.5 | 2.9 | 2.7 | 30.4 | 5.2 |
| 2259 | 0.5 | 0.9 | 1.1 | 31.4 | 4.8 |
| 2511 | 0.5 | 1.1 | 5.5 | 28.8 | 4.8 |
| 2242 | 0.5 | 9.1 | 21.9 | 44.4 | 7.7 |
| 2169 | 0.5 | 3.0 | 12.8 | 31.4 | 4.9 |
| 2208 | 0.6 | 2.0 | 0.6 | 34.2 | 4.9 |
| 3301 | 0.6 | 2.0 | 1.1 | 34.2 | 5.0 |
| 2292 | 0.6 | 1.0 | 15.7 | 33.6 | 5.2 |
| 2750 | 0.6 | 2.5 | 17.0 | 32.2 | 5.3 |
| 2226 | 0.6 | 13.5 | 18.9 | 34.9 | 4.9 |
| 2230 | 0.6 | 12.5 | 18.0 | 38.5 | 5.5 |
| 2237 | 0.7 | 1.9 | 10.1 | 27.1 | 4.8 |
| 2284 | 0.7 | 12.3 | 23.9 | 39.5 | 4.9 |
| 2544 | 0.7 | 19.0 | 17.7 | 43.3 | 10.2 |
| 3485 | 0.7 | 4.9 | 9.0 | 48.8 | 5.9 |
| 3324 | 0.7 | 12.9 | 24.9 | 48.9 | 6.0 |
| 2751 | 0.7 | 3.1 | 16.6 | 33.4 | 5.3 |
| 1448 | 0.8 | 1.1 | 4.4 | 31.8 | 5.1 |
| 2182 | 0.8 | 1.6 | 0.0 | 28.4 | 4.8 |
| 2239 | 0.8 | 0.7 | -3.1 | 31.0 | 4.8 |
| 2218 | 0.8 | 3.0 | 14.3 | 41.4 | 5.3 |
| 3319 | 0.8 | 6.2 | 15.2 | 30.4 | 5.3 |
| 2172 | 0.9 | 13.9 | 26.4 | 35.2 | 5.9 |
| 2748 | 0.9 | 10.5 | 30.6 | 45.1 | 6.4 |
| 2524 | 0.9 | 16.8 | 28.6 | 57.5 | 13.4 |
| 3304 | 0.9 | 2.2 | 1.7 | 35.0 | 5.3 |
| 2747 | 0.9 | 16.4 | 54.2 | 49.4 | 11.8 |
| 2753 | 1.0 | 2.6 | 17.2 | 38.3 | 5.0 |
| 2235 | 1.0 | 14.5 | 36.9 | 44.6 | 5.1 |
| 2201 | 1.0 | 9.5 | 1.2 | 29.9 | 5.1 |
| 3326 | 1.0 | 20.4 | 42.1 | 40.0 | 10.6 |
| 2263 | 1.0 | 1.9 | 12.1 | 29.9 | 4.9 |
| 3332 | 1.0 | 1.8 | 6.7 | 34.3 | 4.9 |
| 2192 | 1.1 | 1.3 | -0.3 | 37.0 | 5.1 |
| 2197 | 1.1 | 4.8 | 11.0 | 33.0 | 5.0 |
| 3101 | 1.1 | 3.3 | 12.2 | 35.9 | 5.2 |
| 2307 | 1.1 | 11.8 | 7.9 | 86.5 | 7.1 |
| 2774 | 1.1 | 7.1 | 31.2 | 41.4 | 4.8 |
| 2286 | 1.2 | 1.3 | 24.4 | 36.6 | 5.6 |
| 2233 | 1.2 | 12.2 | 39.5 | 50.2 | 6.3 |
| 2196 | 1.2 | 6.9 | 7.3 | 31.2 | 4.9 |
| 3313 | 1.2 | 13.6 | 5.1 | 37.9 | 5.5 |
| 2752 | 1.2 | 2.2 | 16.1 | 27.0 | 4.8 |
| 3100 | 1.3 | 4.3 | 13.4 | 31.0 | 4.8 |
| 3327 | 1.4 | 33.5 | 42.7 | 57.7 | 15.7 |
| 2696 | 1.4 | 13.4 | 23.5 | 33.9 | 5.7 |
| 2193 | 1.4 | 3.2 | 4.9 | 35.1 | 5.1 |
| 2225 | 1.4 | 7.6 | 25.9 | 35.2 | 5.4 |
| 3308 | 1.4 | 13.0 | 38.1 | 41.2 | 9.6 |
| 2189 | 1.4 | 11.0 | 14.2 | 31.3 | 4.9 |
| 2685 | 1.4 | 15.9 | -6.2 | 31.4 | 5.2 |
| 3311 | 1.4 | 4.5 | 4.4 | 37.1 | 5.0 |
| 2203 | 1.6 | 2.6 | -3.8 | 34.9 | 5.3 |
| 3295 | 1.6 | 3.0 | 9.3 | 52.3 | 5.7 |
| 2542 | 1.7 | 11.1 | 15.7 | 42.2 | 9.6 |
| 2273 | 1.8 | 6.0 | 19.1 | 36.4 | 5.1 |
| 3099 | 1.8 | 1.4 | -1.7 | 27.7 | 5.5 |
| 2306 | 1.9 | 18.2 | 49.3 | 41.2 | 6.5 |
| 2678 | 2.0 | 17.5 | 54.0 | 55.3 | 8.9 |
| 3483 | 2.0 | 2.6 | 12.1 | 34.2 | 5.1 |
| 2757 | 2.0 | 28.2 | 40.0 | 52.6 | 7.6 |
| 2200 | 2.1 | 2.4 | -2.7 | 28.5 | 5.1 |
| 2177 | 2.1 | 26.7 | 59.1 | 56.5 | 16.9 |
| 2668 | 2.2 | 32.5 | 46.5 | 57.4 | 7.1 |
| 3285 | 2.3 | 14.3 | 33.3 | 45.5 | 7.5 |
| 2183 | 2.3 | 6.7 | 44.9 | 47.8 | 17.9 |
| 2236 | 2.4 | 1.9 | 9.8 | 30.6 | 5.1 |
| 3277 | 2.5 | 4.2 | 29.2 | 43.5 | 7.5 |
| 2288 | 2.5 | 42.3 | 110.6 | 102.4 | 64.1 |
| 2173 | 2.6 | 9.3 | 30.5 | 46.9 | 7.6 |
| 2254 | 2.7 | 20.3 | 70.2 | 43.9 | 6.1 |
| 2498 | 2.7 | 24.6 | 23.0 | 51.4 | 6.4 |
| 2672 | 2.9 | 17.6 | 55.5 | 45.7 | 5.5 |
| 2468 | 2.9 | 29.3 | 24.5 | 42.6 | 7.3 |
| 2480 | 3.1 | 48.2 | 109.7 | 97.6 | 62.8 |
| 3286 | 3.1 | 26.4 | 58.1 | 51.7 | 17.8 |
| 2178 | 3.2 | 12.3 | 39.1 | 53.9 | 7.7 |
| 2190 | 3.2 | 19.4 | 36.4 | 40.2 | 5.5 |
| 2250 | 3.2 | 17.6 | 54.1 | 58.2 | 12.4 |
| 2179 | 3.3 | 15.0 | 48.5 | 45.1 | 11.4 |
| 2676 | 3.3 | 37.6 | 66.5 | 93.3 | 45.8 |
| 2268 | 3.5 | 5.4 | 28.7 | 46.0 | 6.1 |
| 2326 | 3.5 | 92.3 | 208.6 | 150.5 | 133.6 |
| 2664 | 3.9 | 15.3 | 29.3 | 42.7 | 6.6 |
| 2205 | 4.0 | 6.3 | 28.6 | 34.3 | 6.0 |
| 2695 | 4.2 | 16.8 | 36.5 | 50.5 | 8.9 |
| 2274 | 4.3 | 35.0 | 98.0 | 88.6 | 45.9 |
| 3102 | 5.0 | 22.7 | 43.2 | 36.5 | 10.2 |
| 2549 | 5.7 | 63.5 | 155.8 | 108.3 | 79.1 |
| 2516 | 5.8 | 35.0 | 78.0 | 121.9 | 63.8 |
| 2245 | 6.0 | 56.9 | 138.8 | 114.8 | 68.1 |
| 3329 | 6.6 | 77.7 | 138.7 | 126.3 | 92.4 |
| 3320 | 6.9 | 36.6 | 69.4 | 79.4 | 35.3 |
| 3309 | 7.5 | 40.9 | 66.4 | 56.6 | 12.1 |
| 2267 | 7.9 | 2.1 | -4.6 | 41.3 | 5.4 |
| 2253 | 7.9 | 41.0 | 132.7 | 109.2 | 97.9 |
| 2210 | 10.7 | 46.2 | 82.9 | 101.1 | 9.6 |
| 2764 | 12.1 | 102.2 | 203.6 | 188.1 | 182.1 |
| 2202 | 12.4 | 50.1 | 73.1 | 119.8 | 25.2 |
| 2509 | 12.9 | 65.1 | 158.7 | 153.5 | 76.3 |
| 2276 | 15.6 | 122.0 | 269.1 | 208.9 | 208.1 |
| 3292 | 16.5 | 96.5 | 172.5 | 118.3 | 103.9 |
| 2204 | 17.1 | 194.8 | 363.1 | 349.2 | 444.7 |
| 3328 | 17.2 | 51.4 | 156.6 | 89.3 | 52.5 |
| 3487 | 17.3 | 80.5 | 130.7 | 139.9 | 106.9 |
| 2466 | 21.2 | 88.3 | 183.4 | 148.9 | 98.8 |
| 2754 | 23.8 | 36.8 | 52.7 | 62.9 | 37.5 |
| 2665 | 109.0 | 2.4 | 12.4 | 34.1 | 4.9 |

## Supplementary Table 2J. Expression of *P8*, probe set id 209230_s_at.

*n* = 243

| **Sample** | **PCR** | **MAS** | **dChip** | **RMA** | **GCRMA** |
| --- | --- | --- | --- | --- | --- |
| 2541 | 0.0 | 27.0 | 89.2 | 84.0 | 6.1 |
| 2220 | 0.0 | 11.1 | 82.5 | 63.0 | 5.4 |
| 2292 | 0.0 | 4.2 | 108.9 | 67.1 | 5.8 |
| 2524 | 0.0 | 23.6 | 96.0 | 79.8 | 6.1 |
| 2296 | 0.0 | 23.8 | 97.2 | 67.2 | 5.9 |
| 2241 | 0.0 | 14.9 | 78.4 | 62.2 | 4.6 |
| 2546 | 0.0 | 4.1 | 93.6 | 75.5 | 5.9 |
| 2230 | 0.0 | 13.7 | 86.6 | 86.2 | 5.8 |
| 2762 | 0.0 | 27.8 | 120.7 | 68.7 | 6.4 |
| 2217 | 0.0 | 10.3 | 67.8 | 70.0 | 5.0 |
| 2686 | 0.0 | 40.8 | 101.3 | 85.0 | 7.1 |
| 2540 | 0.0 | 25.7 | 93.3 | 80.6 | 5.9 |
| 2306 | 0.0 | 16.3 | 73.8 | 60.2 | 5.3 |
| 2692 | 0.0 | 28.2 | 100.1 | 92.7 | 7.3 |
| 3493 | 0.0 | 11.2 | 71.2 | 69.8 | 5.6 |
| 2226 | 0.0 | 29.7 | 93.7 | 70.6 | 5.7 |
| 2182 | 0.0 | 26.8 | 100.5 | 82.1 | 5.8 |
| 2185 | 0.0 | 20.6 | 93.1 | 77.3 | 5.6 |
| 2509 | 0.0 | 35.5 | 89.4 | 86.5 | 6.5 |
| 2772 | 0.0 | 21.7 | 82.1 | 85.0 | 6.3 |
| 2525 | 0.1 | 12.0 | 119.9 | 82.5 | 6.1 |
| 2271 | 0.1 | 21.7 | 72.0 | 71.7 | 5.3 |
| 2480 | 0.1 | 25.7 | 89.1 | 67.4 | 5.5 |
| 3313 | 0.1 | 44.4 | 104.3 | 67.1 | 6.8 |
| 2179 | 0.1 | 24.7 | 83.6 | 78.9 | 5.3 |
| 2187 | 0.1 | 28.5 | 92.7 | 81.2 | 5.8 |
| 2291 | 0.1 | 21.5 | 87.8 | 71.1 | 5.7 |
| 2694 | 0.1 | 34.1 | 139.8 | 97.7 | 6.2 |
| 2282 | 0.1 | 32.2 | 80.8 | 74.2 | 5.6 |
| 3490 | 0.1 | 5.5 | 56.7 | 74.9 | 6.1 |
| 2695 | 0.1 | 5.7 | 107.8 | 93.8 | 6.2 |
| 3330 | 0.1 | 22.2 | 61.7 | 71.2 | 5.7 |
| 2200 | 0.1 | 17.8 | 93.8 | 80.2 | 6.2 |
| 2274 | 0.1 | 20.5 | 92.1 | 71.6 | 5.8 |
| 2757 | 0.1 | 34.8 | 114.4 | 90.0 | 6.8 |
| 2305 | 0.1 | 27.2 | 79.6 | 96.8 | 6.0 |
| 2766 | 0.1 | 25.8 | 94.9 | 83.2 | 5.8 |
| 3304 | 0.1 | 17.7 | 133.5 | 70.5 | 6.0 |
| 2196 | 0.1 | 25.6 | 91.9 | 71.9 | 5.8 |
| 3102 | 0.1 | 19.8 | 88.5 | 66.3 | 5.5 |
| 2252 | 0.1 | 30.7 | 77.9 | 90.9 | 5.9 |
| 2767 | 0.1 | 17.8 | 74.6 | 62.7 | 5.6 |
| 3484 | 0.1 | 19.9 | 82.6 | 78.3 | 5.3 |
| 3327 | 0.1 | 8.6 | 97.0 | 78.3 | 6.5 |
| 3285 | 0.1 | 18.0 | 92.8 | 78.2 | 6.0 |
| 2299 | 0.1 | 19.3 | 98.8 | 87.8 | 6.8 |
| 3325 | 0.2 | 23.7 | 84.8 | 73.0 | 5.7 |
| 322 | 0.2 | 17.2 | 61.6 | 67.4 | 5.4 |
| 2683 | 0.2 | 38.2 | 95.8 | 95.0 | 7.7 |
| 1766 | 0.2 | 32.7 | 93.3 | 78.0 | 5.9 |
| 2680 | 0.2 | 21.8 | 94.7 | 74.8 | 5.8 |
| 2671 | 0.2 | 25.2 | 93.9 | 84.1 | 6.0 |
| 1448 | 0.2 | 20.6 | 101.0 | 92.0 | 6.5 |
| 2687 | 0.2 | 36.7 | 120.8 | 85.9 | 8.0 |
| 3333 | 0.2 | 5.2 | 49.4 | 71.3 | 6.0 |
| 3099 | 0.2 | 10.5 | 125.3 | 103.1 | 6.4 |
| 3312 | 0.2 | 19.0 | 87.2 | 63.5 | 5.4 |
| 3309 | 0.2 | 23.2 | 107.0 | 84.4 | 6.4 |
| 2535 | 0.2 | 11.8 | 105.7 | 65.0 | 5.9 |
| 2678 | 0.2 | 21.7 | 93.1 | 73.9 | 5.4 |
| 3492 | 0.2 | 26.3 | 63.9 | 64.0 | 6.0 |
| 2236 | 0.2 | 28.8 | 81.8 | 78.9 | 6.3 |
| 2747 | 0.2 | 21.7 | 80.4 | 70.5 | 5.5 |
| 2267 | 0.2 | 32.7 | 70.5 | 66.8 | 6.1 |
| 2231 | 0.2 | 7.2 | 88.9 | 84.7 | 5.4 |
| 3098 | 0.3 | 31.4 | 113.3 | 81.3 | 6.2 |
| 2756 | 0.3 | 31.9 | 98.1 | 87.5 | 6.3 |
| 2278 | 0.3 | 29.8 | 103.0 | 88.5 | 6.1 |
| 2240 | 0.3 | 19.3 | 72.9 | 65.5 | 5.3 |
| 1432 | 0.3 | 9.9 | 67.0 | 79.7 | 5.6 |
| 2204 | 0.3 | 28.6 | 103.1 | 83.8 | 6.5 |
| 3487 | 0.3 | 29.7 | 97.8 | 70.3 | 5.8 |
| 2261 | 0.3 | 14.8 | 75.1 | 67.4 | 5.0 |
| 3331 | 0.3 | 11.4 | 62.2 | 62.9 | 5.3 |
| 3308 | 0.3 | 14.4 | 94.1 | 97.8 | 5.7 |
| 1551 | 0.3 | 14.9 | 44.6 | 62.2 | 5.7 |
| 2218 | 0.4 | 20.2 | 90.6 | 85.3 | 5.5 |
| 3319 | 0.4 | 12.8 | 106.1 | 62.4 | 5.8 |
| 2235 | 0.4 | 16.8 | 76.1 | 64.8 | 5.3 |
| 2281 | 0.4 | 14.7 | 84.8 | 74.2 | 5.6 |
| 3279 | 0.4 | 20.0 | 88.8 | 82.8 | 5.2 |
| 2468 | 0.4 | 28.7 | 129.1 | 81.7 | 6.5 |
| 2497 | 0.4 | 6.6 | 107.8 | 84.1 | 6.6 |
| 3096 | 0.4 | 22.1 | 107.7 | 76.8 | 5.5 |
| 3329 | 0.4 | 43.2 | 86.8 | 73.3 | 6.1 |
| 2679 | 0.4 | 31.5 | 101.6 | 80.0 | 6.1 |
| 2268 | 0.5 | 15.6 | 144.9 | 83.7 | 6.2 |
| 2190 | 0.5 | 16.6 | 97.7 | 66.7 | 5.9 |
| 2242 | 0.5 | 37.3 | 91.0 | 84.3 | 6.4 |
| 3115 | 0.5 | 15.4 | 144.8 | 79.6 | 6.4 |
| 2655 | 0.5 | 17.1 | 96.1 | 82.3 | 5.7 |
| 2685 | 0.5 | 17.0 | 130.9 | 90.1 | 6.1 |
| 3314 | 0.5 | 13.2 | 72.2 | 64.8 | 5.2 |
| 3326 | 0.5 | 22.6 | 87.5 | 67.3 | 5.3 |
| 2672 | 0.5 | 17.4 | 72.1 | 72.0 | 5.4 |
| 3315 | 0.5 | 49.4 | 98.5 | 66.5 | 7.3 |
| 3311 | 0.6 | 17.0 | 92.8 | 75.3 | 5.8 |
| 3295 | 0.6 | 38.5 | 159.6 | 133.5 | 9.6 |
| 2260 | 0.6 | 21.3 | 74.6 | 70.7 | 5.5 |
| 2191 | 0.6 | 17.9 | 78.8 | 70.6 | 5.3 |
| 3301 | 0.6 | 30.7 | 101.7 | 86.5 | 5.8 |
| 2254 | 0.6 | 15.6 | 72.6 | 63.1 | 5.4 |
| 2205 | 0.6 | 12.8 | 95.4 | 71.8 | 5.6 |
| 2753 | 0.6 | 4.7 | 97.4 | 68.4 | 5.6 |
| 2173 | 0.6 | 47.1 | 96.2 | 87.2 | 8.3 |
| 3334 | 0.6 | 32.6 | 83.1 | 73.6 | 5.9 |
| 3328 | 0.6 | 24.9 | 106.3 | 65.8 | 6.0 |
| 2273 | 0.7 | 12.6 | 86.9 | 73.3 | 5.8 |
| 3485 | 0.7 | 46.9 | 88.0 | 103.2 | 7.2 |
| 2257 | 0.7 | 14.4 | 84.9 | 81.8 | 6.0 |
| 2764 | 0.7 | 22.6 | 187.5 | 71.2 | 6.5 |
| 2249 | 0.7 | 20.8 | 70.3 | 73.8 | 5.7 |
| 2253 | 0.8 | 22.0 | 102.4 | 92.8 | 6.0 |
| 2300 | 0.8 | 23.0 | 105.9 | 66.4 | 6.0 |
| 2239 | 0.8 | 25.4 | 81.5 | 82.8 | 5.5 |
| 2219 | 0.8 | 13.8 | 77.3 | 72.0 | 5.5 |
| 2536 | 0.8 | 21.1 | 115.8 | 99.5 | 7.5 |
| 2248 | 0.8 | 23.3 | 80.9 | 61.6 | 5.9 |
| 2206 | 0.8 | 17.4 | 107.0 | 68.0 | 5.7 |
| 2276 | 0.8 | 20.8 | 110.7 | 75.5 | 6.1 |
| 2644 | 0.8 | 23.7 | 162.0 | 73.1 | 6.1 |
| 2222 | 0.8 | 40.6 | 63.9 | 83.2 | 6.6 |
| 2263 | 0.8 | 28.5 | 84.5 | 81.6 | 5.5 |
| 2668 | 0.8 | 11.2 | 120.1 | 80.5 | 5.8 |
| 2195 | 0.8 | 19.9 | 78.3 | 64.1 | 5.5 |
| 2184 | 0.9 | 13.8 | 89.6 | 81.9 | 5.5 |
| 1316 | 0.9 | 24.3 | 88.2 | 89.7 | 6.3 |
| 3323 | 0.9 | 6.2 | 77.3 | 71.8 | 5.8 |
| 2550 | 1.0 | 21.1 | 108.8 | 66.2 | 5.5 |
| 3293 | 1.0 | 31.4 | 99.2 | 83.8 | 6.0 |
| 1299 | 1.0 | 30.7 | 108.6 | 88.1 | 6.6 |
| 2245 | 1.1 | 2.0 | 66.6 | 71.7 | 5.4 |
| 2198 | 1.1 | 23.0 | 77.2 | 85.4 | 5.8 |
| 1747 | 1.1 | 23.6 | 74.4 | 64.8 | 5.2 |
| 1188 | 1.1 | 23.0 | 58.5 | 58.3 | 5.8 |
| 2238 | 1.1 | 10.2 | 71.9 | 63.7 | 5.3 |
| 2212 | 1.1 | 7.2 | 97.3 | 84.0 | 5.9 |
| 3310 | 1.1 | 38.3 | 49.7 | 110.2 | 7.4 |
| 3322 | 1.2 | 24.5 | 72.4 | 70.8 | 5.6 |
| 2647 | 1.2 | 24.6 | 134.9 | 95.6 | 7.3 |
| 2543 | 1.2 | 26.4 | 116.5 | 85.1 | 6.1 |
| 2490 | 1.2 | 10.5 | 79.6 | 77.5 | 6.0 |
| 2545 | 1.2 | 25.5 | 104.1 | 81.9 | 6.1 |
| 2210 | 1.2 | 28.1 | 110.8 | 87.3 | 6.5 |
| 2748 | 1.3 | 4.3 | 97.0 | 73.8 | 5.5 |
| 3318 | 1.3 | 17.7 | 81.5 | 66.8 | 5.4 |
| 2202 | 1.3 | 7.8 | 113.8 | 107.4 | 6.4 |
| 3489 | 1.3 | 13.9 | 93.8 | 77.6 | 5.5 |
| 3277 | 1.3 | 25.9 | 80.4 | 76.1 | 6.2 |
| 2516 | 1.4 | 6.3 | 116.1 | 81.6 | 6.1 |
| 2327 | 1.4 | 25.5 | 76.6 | 70.9 | 5.7 |
| 3324 | 1.4 | 21.1 | 71.9 | 69.1 | 5.6 |
| 2175 | 1.4 | 20.5 | 91.1 | 63.8 | 5.0 |
| 2246 | 1.4 | 9.5 | 63.2 | 71.0 | 5.0 |
| 2507 | 1.4 | 27.6 | 74.3 | 75.4 | 5.7 |
| 2688 | 1.4 | 47.1 | 167.3 | 153.3 | 8.7 |
| 2244 | 1.5 | 12.5 | 102.5 | 85.6 | 5.9 |
| 2544 | 1.5 | 34.2 | 90.7 | 86.8 | 6.3 |
| 2676 | 1.5 | 21.0 | 126.8 | 87.4 | 6.2 |
| 2225 | 1.6 | 9.2 | 77.4 | 64.6 | 4.9 |
| 1201 | 1.6 | 12.1 | 53.9 | 90.8 | 5.7 |
| 2689 | 1.7 | 25.3 | 139.7 | 78.6 | 6.0 |
| 2201 | 1.7 | 14.9 | 100.6 | 77.2 | 5.8 |
| 3100 | 1.7 | 10.9 | 71.0 | 71.7 | 5.3 |
| 2228 | 1.7 | 25.4 | 86.4 | 77.7 | 5.7 |
| 2178 | 1.7 | 17.3 | 80.4 | 76.2 | 6.0 |
| 2237 | 1.7 | 29.2 | 75.1 | 72.7 | 6.5 |
| 3483 | 1.8 | 30.5 | 79.6 | 83.8 | 5.9 |
| 2207 | 1.8 | 25.9 | 75.5 | 83.6 | 5.9 |
| 2510 | 1.8 | 17.7 | 72.5 | 77.1 | 5.5 |
| 2192 | 1.8 | 23.0 | 72.0 | 69.0 | 5.7 |
| 3491 | 1.8 | 18.9 | 81.2 | 65.1 | 5.3 |
| 2172 | 1.8 | 5.7 | 90.1 | 68.5 | 5.7 |
| 2286 | 1.9 | 2.8 | 95.9 | 74.4 | 6.2 |
| 2209 | 1.9 | 10.9 | 121.6 | 84.2 | 6.9 |
| 2682 | 1.9 | 13.4 | 136.9 | 73.1 | 5.9 |
| 2696 | 1.9 | 28.8 | 95.2 | 78.0 | 6.1 |
| 2251 | 1.9 | 50.6 | 88.9 | 141.8 | 9.6 |
| 2259 | 2.0 | 22.1 | 87.0 | 84.8 | 5.7 |
| 2275 | 2.0 | 29.6 | 95.5 | 75.8 | 5.8 |
| 1197 | 2.0 | 19.6 | 90.8 | 91.6 | 6.0 |
| 3101 | 2.0 | 9.2 | 78.3 | 75.7 | 5.2 |
| 2208 | 2.0 | 20.8 | 93.7 | 81.1 | 5.8 |
| 2551 | 2.1 | 24.4 | 118.4 | 83.6 | 7.0 |
| 2534 | 2.2 | 29.6 | 120.6 | 93.6 | 6.1 |
| 2216 | 2.2 | 21.8 | 99.9 | 68.7 | 5.7 |
| 2658 | 2.3 | 21.7 | 83.1 | 68.8 | 5.4 |
| 2189 | 2.3 | 9.7 | 92.6 | 73.2 | 5.4 |
| 2769 | 2.3 | 36.4 | 125.5 | 78.3 | 6.7 |
| 2285 | 2.5 | 24.6 | 67.1 | 60.4 | 5.4 |
| 2754 | 2.6 | 21.8 | 106.1 | 83.1 | 6.1 |
| 2214 | 2.7 | 8.5 | 85.7 | 66.7 | 5.2 |
| 2203 | 2.7 | 20.1 | 137.4 | 107.5 | 7.0 |
| 2171 | 2.9 | 13.0 | 85.1 | 64.3 | 5.3 |
| 2234 | 3.1 | 26.7 | 79.2 | 84.7 | 5.8 |
| 2250 | 3.1 | 21.1 | 69.9 | 72.9 | 5.2 |
| 2176 | 3.3 | 15.5 | 83.3 | 63.5 | 5.0 |
| 2188 | 3.3 | 27.6 | 86.1 | 73.5 | 5.3 |
| 1595 | 3.4 | 21.2 | 65.0 | 76.8 | 5.3 |
| 2183 | 3.5 | 10.1 | 88.5 | 80.4 | 5.0 |
| 2170 | 3.5 | 25.6 | 123.5 | 78.8 | 5.9 |
| 2194 | 3.6 | 24.3 | 85.4 | 73.0 | 5.5 |
| 2186 | 3.8 | 18.3 | 79.3 | 66.2 | 5.3 |
| 3278 | 3.9 | 4.7 | 82.1 | 78.7 | 5.8 |
| 2669 | 4.0 | 34.3 | 81.8 | 87.5 | 6.0 |
| 2749 | 4.0 | 28.5 | 72.4 | 76.7 | 5.8 |
| 2656 | 4.0 | 12.8 | 73.0 | 67.2 | 5.4 |
| 3292 | 4.1 | 24.9 | 171.8 | 72.0 | 6.5 |
| 2197 | 4.1 | 27.0 | 91.9 | 68.4 | 5.8 |
| 2666 | 4.3 | 8.5 | 99.3 | 69.4 | 5.1 |
| 2233 | 4.4 | 27.8 | 61.6 | 79.1 | 5.6 |
| 2262 | 4.5 | 3.9 | 81.7 | 68.3 | 5.7 |
| 2771 | 5.0 | 18.0 | 96.3 | 74.1 | 5.8 |
| 2174 | 5.5 | 14.9 | 99.1 | 73.5 | 6.2 |
| 3286 | 5.5 | 24.6 | 100.8 | 89.7 | 6.3 |
| 3486 | 5.6 | 16.0 | 87.9 | 69.2 | 6.0 |
| 3481 | 5.7 | 39.3 | 74.4 | 104.6 | 6.3 |
| 2193 | 6.0 | 5.7 | 105.9 | 71.2 | 5.8 |
| 2265 | 6.1 | 16.4 | 93.5 | 73.4 | 5.9 |
| 3320 | 6.4 | 8.0 | 88.0 | 104.8 | 5.9 |
| 2466 | 7.2 | 26.7 | 130.3 | 83.5 | 7.3 |
| 2750 | 7.7 | 27.3 | 90.2 | 73.9 | 6.4 |
| 3332 | 7.8 | 17.7 | 74.8 | 77.2 | 5.5 |
| 3316 | 9.1 | 32.3 | 103.5 | 93.9 | 7.0 |
| 2177 | 9.7 | 19.1 | 77.4 | 69.5 | 5.6 |
| 2247 | 9.8 | 12.9 | 80.1 | 80.3 | 5.5 |
| 2199 | 9.8 | 25.9 | 94.1 | 72.5 | 6.0 |
| 3097 | 10.1 | 18.6 | 88.8 | 81.9 | 5.5 |
| 2256 | 11.4 | 35.5 | 72.2 | 88.7 | 5.8 |
| 2224 | 13.3 | 33.7 | 63.9 | 80.7 | 5.6 |
| 3488 | 13.6 | 18.3 | 86.2 | 67.6 | 5.5 |
| 2307 | 14.7 | 45.0 | 49.4 | 83.6 | 7.3 |
| 3482 | 16.0 | 35.6 | 80.5 | 94.8 | 6.4 |
| 3321 | 17.5 | 26.4 | 90.1 | 82.4 | 5.7 |
| 2243 | 18.4 | 7.5 | 96.8 | 87.7 | 6.6 |
| 1174 | 19.4 | 37.4 | 75.7 | 81.7 | 5.6 |
| 3289 | 20.1 | 34.1 | 121.0 | 83.5 | 6.0 |
| 1401 | 24.5 | 25.7 | 82.1 | 94.3 | 6.1 |
| 2498 | 25.0 | 20.4 | 127.9 | 83.5 | 7.4 |
| 2670 | 30.5 | 42.0 | 102.8 | 104.0 | 6.5 |
| 2181 | 43.4 | 30.9 | 91.8 | 78.9 | 8.8 |
| 2667 | 57.1 | 41.9 | 122.4 | 110.7 | 10.1 |
| 2665 | 230.7 | 19.3 | 113.7 | 84.3 | 6.1 |

## Supplementary Table 3A. Overlap between sets of genes marked as differentially expressed after pre-processing with different methods. SAM *q*-values and *t*-test and Wilcoxon test *p*-values were calculated for the significance of difference in expression between samples from the AML dataset with inversion(16) and samples without inversion(16). Overlap is represented as the result of twice the overlap divided by the number of probe sets in both sets. The number on the diagonal represents the number of probe sets marked as differentially expressed, for that method.

|  |  | MAS | dChip | RMA | GCRMA |
| --- | --- | --- | --- | --- | --- |
| **SAM *q*-values** | MAS | 4458 |  |  |  |
|  | dChip | 0.66 | 3185 |  |  |
|  | RMA | 0.69 | 0.65 | 4769 |  |
|  | GCRMA | 0.69 | 0.67 | 0.83 | 4457 |
| *t*-test*p*-values | MAS | 1731 |  |  |  |
|  | dChip | 0.72 | 1736 |  |  |
|  | RMA | 0.73 | 0.70 | 2059 |  |
|  | GCRMA | 0.71 | 0.70 | 0.84 | 2290 |
| Wilcoxon test*p*-values | MAS | 487 |  |  |  |
|  | dChip | 0.74 | 478 |  |  |
|  | RMA | 0.80 | 0.80 | 549 |  |
|  | GCRMA | 0.81 | 0.81 | 0.87 | 556 |

**Supplementary Table 3B.** AML dataset, samples with translocation (15;17) versus samples without translocation (15;17).

|  |  | MAS | dChip | RMA | GCRMA |
| --- | --- | --- | --- | --- | --- |
| **SAM *q*-values** | MAS | 5100 |  |  |  |
|  | dChip | 0.69 | 5097 |  |  |
|  | RMA | 0.77 | 0.70 | 5457 |  |
|  | GCRMA | 0.77 | 0.69 | 0.86 | 5097 |
| *t*-test*p*-values | MAS | 1474 |  |  |  |
|  | dChip | 0.71 | 1467 |  |  |
|  | RMA | 0.70 | 0.71 | 1715 |  |
|  | GCRMA | 0.73 | 0.71 | 0.85 | 1755 |
| Wilcoxon test*p*-values | MAS | 767 |  |  |  |
|  | dChip | 0.79 | 757 |  |  |
|  | RMA | 0.83 | 0.81 | 835 |  |
|  | GCRMA | 0.84 | 0.80 | 0.89 | 853 |

**Supplementary Table 3C.** AML dataset, samples with translocation (8;21) versus samples without translocation (8;21).

|  |  | MAS | dChip | RMA | GCRMA |
| --- | --- | --- | --- | --- | --- |
| **SAM *q*-values** | MAS | 4175 |  |  |  |
|  | dChip | 0.72 | 4176 |  |  |
|  | RMA | 0.77 | 0.75 | 3902 |  |
|  | GCRMA | 0.76 | 0.75 | 0.88 | 3653 |
| *t*-test*p*-values | MAS | 1361 |  |  |  |
|  | dChip | 0.56 | 918 |  |  |
|  | RMA | 0.57 | 0.67 | 1016 |  |
|  | GCRMA | 0.60 | 0.68 | 0.78 | 1010 |
| Wilcoxon test*p*-values | MAS | 710 |  |  |  |
|  | dChip | 0.63 | 552 |  |  |
|  | RMA | 0.67 | 0.75 | 537 |  |
|  | GCRMA | 0.70 | 0.75 | 0.85 | 571 |

**Supplementary Table 3D.** CNS dataset, PNET samples versus others.

|  |  | MAS | dChip | RMA | GCRMA |
| --- | --- | --- | --- | --- | --- |
| **SAM *q*-values** | MAS | 1442 |  |  |  |
|  | dChip | 0.06 | 661 |  |  |
|  | RMA | 0.44 | 0.16 | 1169 |  |
|  | GCRMA | 0.10 | 0.18 | 0.33 | 347 |
| *t*-test*p*-values | MAS | 33 |  |  |  |
|  | dChip | 0.08 | 18 |  |  |
|  | RMA | 0.05 | 0.08 | 6 |  |
|  | GCRMA | 0.0 | 0.0 | 0.0 | 1 |
| Wilcoxon test*p*-values | MAS | 0 |  |  |  |
|  | dChip | 0.0 | 0 |  |  |
|  | RMA | 0.0 | 0.0 | 0 |  |
|  | GCRMA | 0.0 | 0.0 | 0.0 | 0 |

**Supplementary Table 3E.** CNS dataset, MED samples versus others.

|  |  | MAS | dChip | RMA | GCRMA |
| --- | --- | --- | --- | --- | --- |
| **SAM *q*-values** | MAS | 364 |  |  |  |
|  | dChip | 0.39 | 370 |  |  |
|  | RMA | 0.50 | 0.54 | 613 |  |
|  | GCRMA | 0.52 | 0.39 | 0.58 | 438 |
| *t*-test*p*-values | MAS | 132 |  |  |  |
|  | dChip | 0.26 | 82 |  |  |
|  | RMA | 0.27 | 0.31 | 36 |  |
|  | GCRMA | 0.28 | 0.28 | 0.41 | 56 |
| Wilcoxon test*p*-values | MAS | 7 |  |  |  |
|  | dChip | 0.17 | 5 |  |  |
|  | RMA | 0.17 | 0.6 | 5 |  |
|  | GCRMA | 0.18 | 0.67 | 0.68 | 4 |

**Supplementary Table 3F.** CNS dataset, RHAB samples versus others.

|  |  | MAS | dChip | RMA | GCRMA |
| --- | --- | --- | --- | --- | --- |
| **SAM *q*-values** | MAS | 4446 |  |  |  |
|  | dChip | 0.55 | 2855 |  |  |
|  | RMA | 0.79 | 0.63 | 4780 |  |
|  | GCRMA | 0.45 | 0.56 | 0.56 | 2012 |
| *t*-test*p*-values | MAS | 858 |  |  |  |
|  | dChip | 0.21 | 613 |  |  |
|  | RMA | 0.19 | 0.44 | 289 |  |
|  | GCRMA | 0.20 | 0.40 | 0.68 | 267 |
| Wilcoxon test*p*-values | MAS | 47 |  |  |  |
|  | dChip | 0.27 | 184 |  |  |
|  | RMA | 0.34 | 0.52 | 160 |  |
|  | GCRMA | 0.44 | 0.48 | 0.67 | 113 |

## Supplementary Table 4: Performance of different classification algorithms. Mean test set error (and standard deviation) over 100 random splits of the original data into a training set (90%) and a test set (10%). Error is defined as average error per class, i.e. corresponding to assuming a prior probability of occurrence of a class of 50%. Classifiers were trained for 10, 20, 50, 100, 250, 500 and 1000 probe sets selected by the variation filter; results shown here are for the number of probe sets resulting in the smallest average test set error over the four methods, indicated between brackets after the classifier name.

1. CNS dataset, PNET problem.
2. CNS dataset, GLIO problem.
3. CNS dataset, RHAB problem.

A.

|  |  | MAS | dChip | RMA | GCRMA |
| --- | --- | --- | --- | --- | --- |
| Classifier  (number of probe sets used) | NC (100) | 0.05 (0.13) | 0.14 (0.20) | 0.17 (0.22) | 0.06 (0.14) |
|  | PAM (10) | 0.04 (0.12) | 0.06 (0.13) | 0.06 (0.13) | 0.02 (0.09) |
|  | LIKNON (1000) | 0.11 (0.18) | 0.01 (0.05) | 0.04 (0.11) | 0.13 (0.18) |
|  | k-NN (500) | 0.04 (0.12) | 0.02 (0.08) | 0.02 (0.08) | 0.05 (0.13) |
|  | SVC/P (1000) | 0.03 (0.10) | 0.01 (0.07) | 0.03 (0.09) | 0.10 (0.18) |
|  | SVC/RBF (1000) | 0.00 (0.00) | 0.00 (0.00) | 0.00 (0.00) | 0.00 (0.00) |

B.

|  |  | MAS | dChip | RMA | GCRMA |
| --- | --- | --- | --- | --- | --- |
| Classifier  (number of probe sets used) | **NC (50)** | 0.07 (0.13) | 0.09 (0.14) | 0.05 (0.11) | 0.12 (0.14) |
|  | **PAM (20)** | 0.03 (0.08) | 0.04 (0.09) | 0.03 (0.08) | 0.11 (0.15) |
|  | **LIKNON (200)** | 0.02 (0.07) | 0.05 (0.10) | 0.04 (0.09) | 0.07 (0.12) |
|  | **k-NN (50)** | 0.03 (0.08) | 0.03 (0.08) | 0.03 (0.08) | 0.08 (0.12) |
|  | **SVC/P (1000)** | 0.03 (0.08) | 0.03 (0.08) | 0.03 (0.08) | 0.06 (0.10) |
|  | **SVC/RBF (20)** | 0.07 (0.11) | 0.06 (0.11) | 0.09 (0.12) | 0.15 (0.12) |

C.

|  |  | MAS | dChip | RMA | GCRMA |
| --- | --- | --- | --- | --- | --- |
| Classifier  (number of probe sets used) | NC (10) | 0.03 (0.08) | 0.06 (0.12) | 0.14 (0.16) | 0.05 (0.10) |
|  | PAM (20) | 0.04 (0.10) | 0.08 (0.15) | 0.12 (0.15) | 0.08 (0.13) |
|  | LIKNON (50) | 0.03 (0.08) | 0.12 (0.17) | 0.08 (0.13) | 0.07 (0.12) |
|  | k-NN (50) | 0.06 (0.12) | 0.07 (0.12) | 0.08 (0.12) | 0.07 (0.12) |
|  | SVC/P (100) | 0.06 (0.11) | 0.05 (0.11) | 0.07 (0.12) | 0.03 (0.08) |
|  | SVC/RBF (50) | 0.12 (0.13) | 0.09 (0.12) | 0.18 (0.11) | 0.13 (0.13) |

## Supplementary Figure 1A. Jaccard indices of clustering results.

Pairwise Jaccard indices between clusterings of differently pre-processed versions of the AML dataset, into *k* = 2 clusters obtained using correlation distance on 3000 probe sets.

Supplementary Figure 1B.

Pairwise Jaccard indices between clusterings of differently pre-processed versions of the AML dataset, into *k* = 20 clusters, obtained using correlation distance on 3000 probe sets.

Supplementary Figure 1C.

Pairwise Jaccard indices between clusterings of differently pre-processed versions of the CNS dataset, into *k* = 2 clusters, obtained using correlation distance on 1000 probe sets.

Supplementary Figure 1D.

Pairwise Jaccard indices between clusterings of differently pre-processed versions of the CNS dataset, into *k* = 10 clusters, obtained using correlation distance on 1000 probe sets.

Supplementary Figure 1E.

Pairwise Jaccard indices between clusterings of differently pre-processed versions of the AML dataset, into *k* = 2 clusters, obtained using Euclidean distance on 3000 probe sets.

Supplementary Figure 1F.

Pairwise Jaccard indices between clusterings of differently pre-processed versions of the AML dataset, into *k* = 12 clusters, obtained using Euclidean distance on 3000 probe sets.

Supplementary Figure 1G.

Pairwise Jaccard indices between clusterings of differently pre-processed versions of the AML dataset, into *k* = 20 clusters, obtained using Euclidean distance on 3000 probe sets.

Supplementary Figure 1H.

Pairwise Jaccard indices between clusterings of differently pre-processed versions of the CNS dataset, into *k* = 2 clusters, obtained using Euclidean distance on 1000 probe sets.

Supplementary Figure 1I.

Pairwise Jaccard indices between clusterings of differently pre-processed versions of the CNS dataset, into *k* = 5 clusters, obtained using Euclidean distance on 1000 probe sets.

Supplementary Figure 1J.

Pairwise Jaccard indices between clusterings of differently pre-processed versions of the CNS dataset, into *k* = 10 clusters, obtained using Euclidean distance on 1000 probe sets.


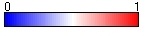


Supplementary Figure 2A: AML dataset: stability-normalized pairwise Jaccard indices of cluster labels assigned by the various methods. Clusterings into *k* = 2 clusters obtained using correlation distance on 3000 probe sets. Legend is shown in Supp. Figure 2K. For *k*-means, the grey bars indicate standard deviation over 10 repeated experiments.

Supplementary Figure 2B: AML dataset: stability-normalized pairwise Jaccard indices of cluster labels assigned by the various methods. Clusterings into *k* = 20 clusters obtained using correlation distance on expression data of 3000 probe sets. Legend is shown in Supp. Figure 2K. For *k*-means, the grey bars indicate standard deviation over 10 repeated experiments.

Supplementary Figure 2C: CNS dataset: stability-normalized pairwise Jaccard indices of cluster labels assigned by the various methods. Clusterings into *k* = 2 clusters obtained using correlation distance on expression data of 1000 probe sets. Legend is shown in Supp. Figure 2K. For *k*-means, the grey bars indicate standard deviation over 10 repeated experiments.

Supplementary Figure 2D: CNS dataset: stability-normalized pairwise Jaccard indices of cluster labels assigned by the various methods. Clusterings into *k* = 10 clusters obtained using correlation distance on expression data of 1000 probe sets. Legend is shown in Supp. Figure 2K. For *k*-means, the grey bars indicate standard deviation over 10 repeated experiments.

Supplementary Figure 2E: AML dataset: stability-normalized pairwise Jaccard indices of cluster labels assigned by the various methods. Clusterings into *k* = 2 clusters obtained using Euclidean distance on 3000 probe sets. Legend is shown in Supp. Figure 2K. For *k*-means, the grey bars indicate standard deviation over 10 repeated experiments.

Supplementary Figure 2F: AML dataset: stability-normalized pairwise Jaccard indices of cluster labels assigned by the various methods. Clusterings into *k* = 12 clusters obtained using Euclidean distance on 3000 probe sets. Legend is shown in Supp. Figure 2K. For *k*-means, the grey bars indicate standard deviation over 10 repeated experiments.

Supplementary Figure 2G: AML dataset: stability-normalized pairwise Jaccard indices of cluster labels assigned by the various methods. Clusterings into *k* = 20 clusters obtained using Euclidean distance on 3000 probe sets. Legend is shown in Supp. Figure 2K. For *k*-means, the grey bars indicate standard deviation over 10 repeated experiments.

Supplementary Figure 2H: CNS dataset: stability-normalized pairwise Jaccard indices of cluster labels assigned by the various methods. Clusterings into *k* = 2 clusters obtained using Euclidean distance on 1000 probe sets. Legend is shown in Supp. Figure 2K. For *k*-means, the grey bars indicate standard deviation over 10 repeated experiments.

Supplementary Figure 2I: CNS dataset: stability-normalized pairwise Jaccard indices of cluster labels assigned by the various methods. Clusterings into *k* = 5 clusters obtained using Euclidean distance on 1000 probe sets. Legend is shown in Supp. Figure 2K. For *k*-means, the grey bars indicate standard deviation over 10 repeated experiments.

Supplementary Figure 2J: CNS dataset: stability-normalized pairwise Jaccard indices of cluster labels assigned by the various methods. Clusterings into *k* = 10 clusters obtained using Euclidean distance on 1000 probe sets. Legend is shown in Supp. Figure 2K. For *k*-means, the grey bars indicate standard deviation over 10 repeated experiments.

Supplementary Figure 2K: Legend to markers in Supp. Figures 2A-J.

MAS (1) vs dChip (2) (2)

MAS (1) vs RMA (2)

MAS (1) vs GCRMA (2)

dChip (1) vs RMA (2)

dChip (1) vs GCRMA (2)

RMA (1) vs GCRMA (2)
